# Endosomal escape of delivered mRNA from endosomal recycling tubules visualized at the nanoscale

**DOI:** 10.1083/jcb.202110137

**Published:** 2021-12-09

**Authors:** Prasath Paramasivam, Christian Franke, Martin Stöter, Andreas Höijer, Stefano Bartesaghi, Alan Sabirsh, Lennart Lindfors, Marianna Yanez Arteta, Anders Dahlén, Annette Bak, Shalini Andersson, Yannis Kalaidzidis, Marc Bickle, Marino Zerial

**Affiliations:** 1 Max Planck Institute of Molecular Cell Biology and Genetics, Dresden, Germany; 2 Advanced Drug Delivery, Pharmaceutical Science Research and Development, AstraZeneca, Gothenburg, Sweden; 3 Bioscience Metabolism, Research and Early Development Cardiovascular, Renal and Metabolism, BioPharmaceuticals Research and Development, AstraZeneca, Gothenburg, Sweden; 4 Oligonucleotide Discovery, Discovery Sciences Research and Development, AstraZeneca, Gothenburg, Sweden; 5 Advanced Drug Delivery, Pharmaceutical Science Research and Development, AstraZeneca, Boston, MA

## Abstract

Delivery of exogenous mRNA using lipid nanoparticles (LNPs) is a promising strategy for therapeutics. However, a bottleneck remains in the poor understanding of the parameters that correlate with endosomal escape versus cytotoxicity. To address this problem, we compared the endosomal distribution of six LNP-mRNA formulations of diverse chemical composition and efficacy, similar to those used in mRNA-based vaccines, in primary human adipocytes, fibroblasts, and HeLa cells. Surprisingly, we found that total uptake is not a sufficient predictor of delivery, and different LNPs vary considerably in endosomal distributions. Prolonged uptake impaired endosomal acidification, a sign of cytotoxicity, and caused mRNA to accumulate in compartments defective in cargo transport and unproductive for delivery. In contrast, early endocytic/recycling compartments have the highest probability for mRNA escape. By using super-resolution microscopy, we could resolve a single LNP-mRNA within subendosomal compartments and capture events of mRNA escape from endosomal recycling tubules. Our results change the view of the mechanisms of endosomal escape and define quantitative parameters to guide the development of mRNA formulations toward higher efficacy and lower cytotoxicity.

## Teaser

Paramasivam et al. compared the endosomal trafficking of six LNP-mRNA formulations. LNP-mRNA accumulates in large endosomes impaired in acidification and unproductive for delivery. Super-resolution microscopy allowed visualization of single LNP-mRNA within endosomes and escape of mRNA from endosomal recycling tubules.

## Introduction

In recent years, RNAs have emerged as potentially powerful therapeutics (Coutinho et al., 2019) to inhibit gene expression (splicing modulators, siRNAs, and antisense oligonucleotides) and express proteins (mRNA) or in gene editing (CRISPR/Cas9 system). An increasing number of RNA-based therapeutics have proven effective for clinical treatment (Akinc et al., 2019; Dammes and Peer, 2020; Sahin et al., 2020). More recently, optimization of chemical and physical properties have focused attention on mRNA-based therapeutics, including vaccines (Feldman et al., 2019; Kose et al., 2019). Major improvements toward clinical application have come from chemical modifications of RNAs that increase stability and reduce immunogenicity. Nevertheless, efficacy remains a crucial challenge due to limited or poor delivery (Kowalski et al., 2019).

Lipid nanoparticles (LNPs) are currently the nonviral RNA delivery platform of choice (Kowalski et al., 2019; Reichmuth et al., 2016). LNPs have different chemical compositions and show vastly different delivery efficiency, toxicity, and immunological liability. The mechanistic basis for such differences is unclear. Since delivery is a multistep process (Wittrup and Lieberman, 2015), attempting structure/activity relation without understanding the underlying mechanisms can yield complex and contradicting results, as the outcome of experiments is influenced by the nonlinear combination of the various steps. Besides endocytosis, a major challenge remains the ability of RNA to cross the endosomal membrane (Pei and Buyanova, 2019). Ineffective escape from endosomes imposes higher dosage, thus causing toxicity. Toxicity is due to both cell-autonomous, i.e., cytotoxicity, and non–cell-autonomous, e.g., inflammation, effects (Reichmuth et al., 2016; Sato et al., 2017). The reasons for cytotoxicity are diverse, comprising oxidative stress and apoptosis (Ahmad et al., 2020). Whether they include alterations of the endosomal system is unknown.

The precise sites and mechanisms whereby LNPs help mRNA to escape from the endosomal lumen are, to date, mysterious. Escape efficiency arguably depends on the distribution of LNPs in various subcellular compartments, of which only a selected few are poised to macromolecule escape. Previous studies have yielded contradictory results on LNP internalization, endosomal distribution, and escape of RNA (siRNA). Whereas we and others (Gilleron et al., 2013; Wittrup et al., 2015) reported that escape is restricted to an early endosomal compartment before conversion into late endosomes (Rink et al., 2005), another study claimed that escape occurs mainly from late endosomes where LNPs accumulate (Sahay et al., 2013). However, this conclusion was based on perturbations (drugs and gene downregulation) that induce pleiotropic effects, making the data difficult to interpret. Furthermore, the complexity of the endosomal network cannot be underestimated. It consists of populations of organelles that are subcompartmentalized (Franke et al., 2019; Sönnichsen et al., 2000), dynamically exchange cargo and trafficking machinery, and change in size and position over time, calling for a thorough quantitative and high-resolution analysis to interpret their precise identities. Besides the endosomal compartments granting RNA escape, the underlying mechanisms remain mysterious. Live cell imaging has shown that lipoplexes deliver siRNAs by endosome bursting, but this mechanism does not apply to LNPs (Gilleron et al., 2013; Wittrup et al., 2015). Despite claims of membrane fusion and imaging of mRNA escape into the cytoplasm (Miao et al., 2020), the sensitivity and resolution of conventional microscopic techniques are insufficient to visualize escape of a small number of mRNAs. For this, single-molecule techniques are essential.

To address this critical problem, we aimed to determine whether differences in LNP-mediated mRNA (LNP-mRNA) delivery efficacy may originate from variations in (1) uptake and/or (2) transport to endosomal subcompartments with higher probability to escape than others. As yet, LNPs have predominantly been investigated for intravenous or intramuscular administration. Subcutaneous administration in adipose tissue opens the possibility of patient self-administration and, hence, long-term treatment that could enable mRNA as a novel modality for protein replacement or regenerative therapies. Therefore, we performed a systematic comparison of six mRNA-containing LNPs with different cationic lipids, including MOD5, an analogue of the SM-102 lipid used in the Moderna “mRNA-1273” SARS-CoV-2 vaccine (Sabnis et al., 2018), in primary human adipocytes, fibroblasts, and HeLa cells to identify candidate parameters diagnostic for efficacy of delivery.

## Results

### LNP-mRNA internalization is necessary but not sufficient to predict delivery efficacy

**Figure 1.**
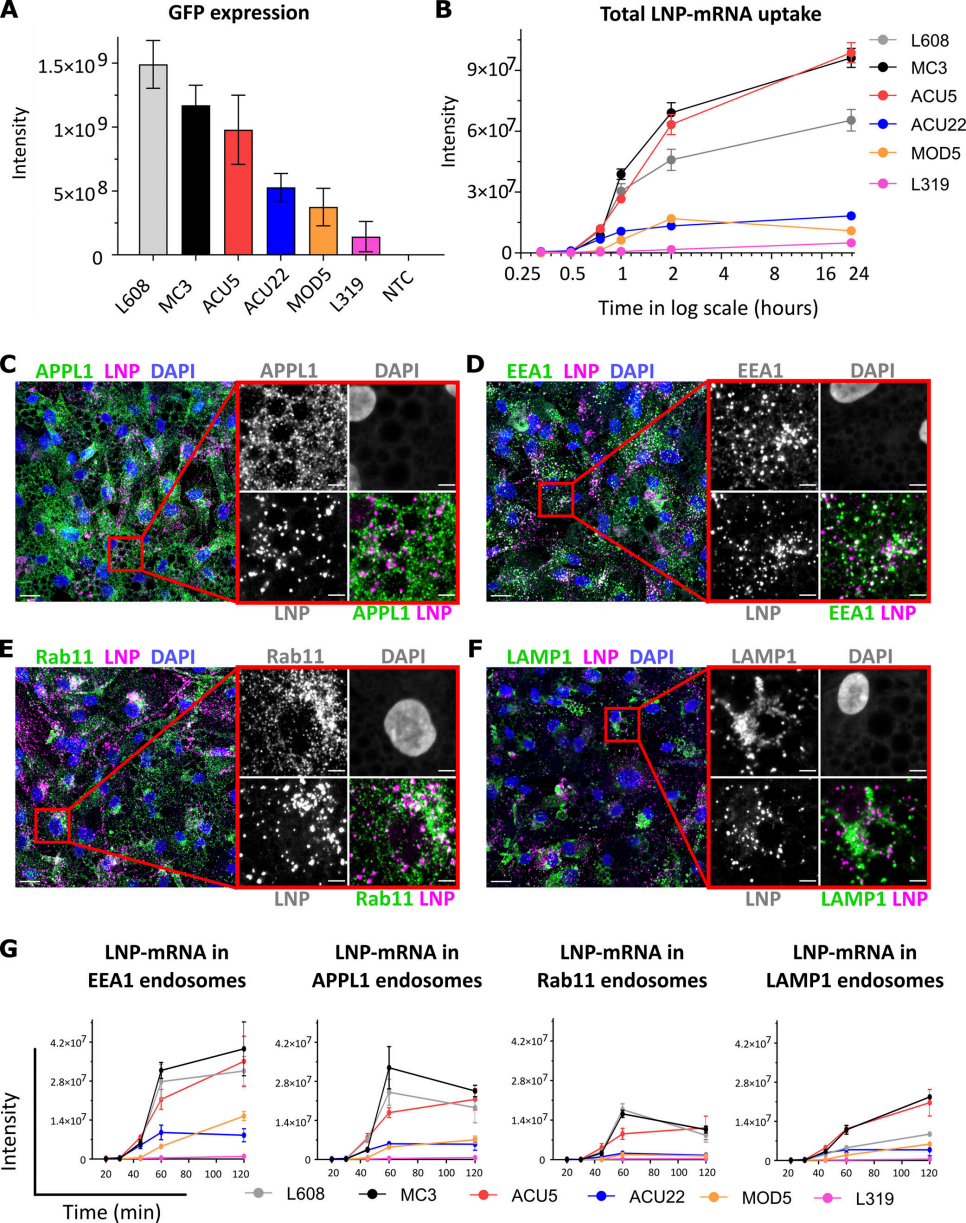
**Comparative analysis of activity, endocytic uptake, and endosomal distribution of six LNP-mRNAs in primary human adipocytes.** **(A)** Cells were incubated with various LNPs (1.25 ng/μl) formulated with eGFP-mRNA, fixed, and imaged after 24 h. The graph illustrates GFP expression for the indicated LNP-mRNA. *n* = 3 independent experiments (mean ± SEM). **(B)** Representative LNP-mRNA uptake kinetics curve. Cells incubated with LNP-mRNA as described above were fixed at the given time point, processed for smFISH to fluorescently label mRNA, and imaged by fluorescence microscopy. The quantification shows that LNP-mRNA uptake generally correlates to the GFP expression efficacy of LNP formulations displayed in A (e.g., MC3 versus L319). **(C–F)** Representative images of human primary adipocytes incubated with L608 LNP-mRNA for 2 h and immunostained with antibodies against endosomal markers (in green) as follows: APPL1 (C), EEA1 (D), Rab11 (E), and LAMP1 (F). Exogenous mRNA was detected by smFISH (labeled as LNP) and nuclei by DAPI. The magnified area is presented with split and merged color images. Scale bars are 20 μm in the overview and 5 μm in the magnified images. **(G)** Representative kinetics and endosomal distribution of the different LNP-mRNAs incubated with cells as described in C–F. *n* = 3 replicates (mean ± SEM). Significant P values for panels A, B, and G and for all LNP combinations are listed in Table S4, Table S5, Table S6, Table S7, Table S8, and Table S9.

We performed a comparative analysis on uptake and endosomal distribution of mRNA encoding eGFP formulated in six distinct LNPs with similar size distribution (54–73 nm) and mRNA content (Table S1) but chosen on the basis of various chemical structures of the cationic lipid (Supplemental figures, Fig. S1); efficacy, as evaluated by intensity of eGFP expression (Fig. 1 A and Supplemental figures, Fig. S2); and toxicity (e.g., O-(Z,Z,Z,Z-heptatriaconta-6,9,26,29-tetraem-19-yl)-4-(N,N-dimethylamino)butanoate [MC3] versus MOD5 or L319; Maier et al., 2013; Sabnis et al., 2018). We used primary human adipocytes, as a relevant cell model for mRNA administered by subcutaneous injection, and detected the mRNA with single-molecule FISH (smFISH), unless otherwise mentioned. We applied image analysis methods to discriminate between adipocytes and fibroblasts in the same culture. Measurement of uptake kinetics indeed showed that internalization and trafficking vary between LNPs and mRNA (Fig. 1 B). Interestingly, although in most cases uptake correlated with transfection efficacy (e.g., MC3 versus L319), this was not a strict rule. L608 demonstrated the highest eGFP expression, despite moderate LNP-mRNA uptake, compared with, e.g., MC3 and ACU5 (Fig. 1 B). Besides uptake efficiency, efficacy may depend on qualitative and/or quantitative differences in endosomal distributions.

Internalized cargo molecules are transported to early endosomes where they are sorted either to recycling endosomes and returned to the plasma membrane or to late endosomes/lysosomes where they are degraded. For LNPs to be effective and nontoxic, they should carry mRNA to compartments where it can be released into the cytoplasm or at least degraded without interfering with endosomal function. Therefore, the average residence time of LNP-mRNA in compartments that are favourable for escape must be rate-limiting for delivery (Gilleron et al., 2013) and proportional to the accumulation of LNP-mRNA in the compartment. To gain insight into these properties, we measured the distribution of endocytosed mRNA in endosomal compartments immunostained against the Rab5 effectors EEA1 and APPL1 to label different types of early endosomes (Kalaidzidis et al., 2015a), Rab11 for early/recycling endosomes (Sönnichsen et al., 2000) and LAMP1 for late endosomes/lysosomes (Fig. 1, C–F, representative images from 2-h time point). Interestingly, the proportion of LNP-mRNA differed quantitatively between compartments. First, LNP-mRNA distribution in EEA1^+^ and APPL1^+^ early endosomes is significantly higher than other compartments (Fig. 1 G). Second, the fraction of LNP-mRNA in early (EEA1, APPL1) and recycling (Rab11) endosomes is higher for the more efficient LNPs (L608, MC3, and ACU5) than the less efficient LNPs (ACU22, MOD5, and L319; P values in Table S6, Table S7, and Table S8). This tendency was not apparent in LAMP1 endosomes (compare L608 with MC3 and ACU5; P values in Table S9), suggesting that this compartment contributes only minimally to mRNA delivery.

### Accumulation of mRNA in Rab11 endosomes is a strong predictor of delivery efficacy

**Figure 2.**
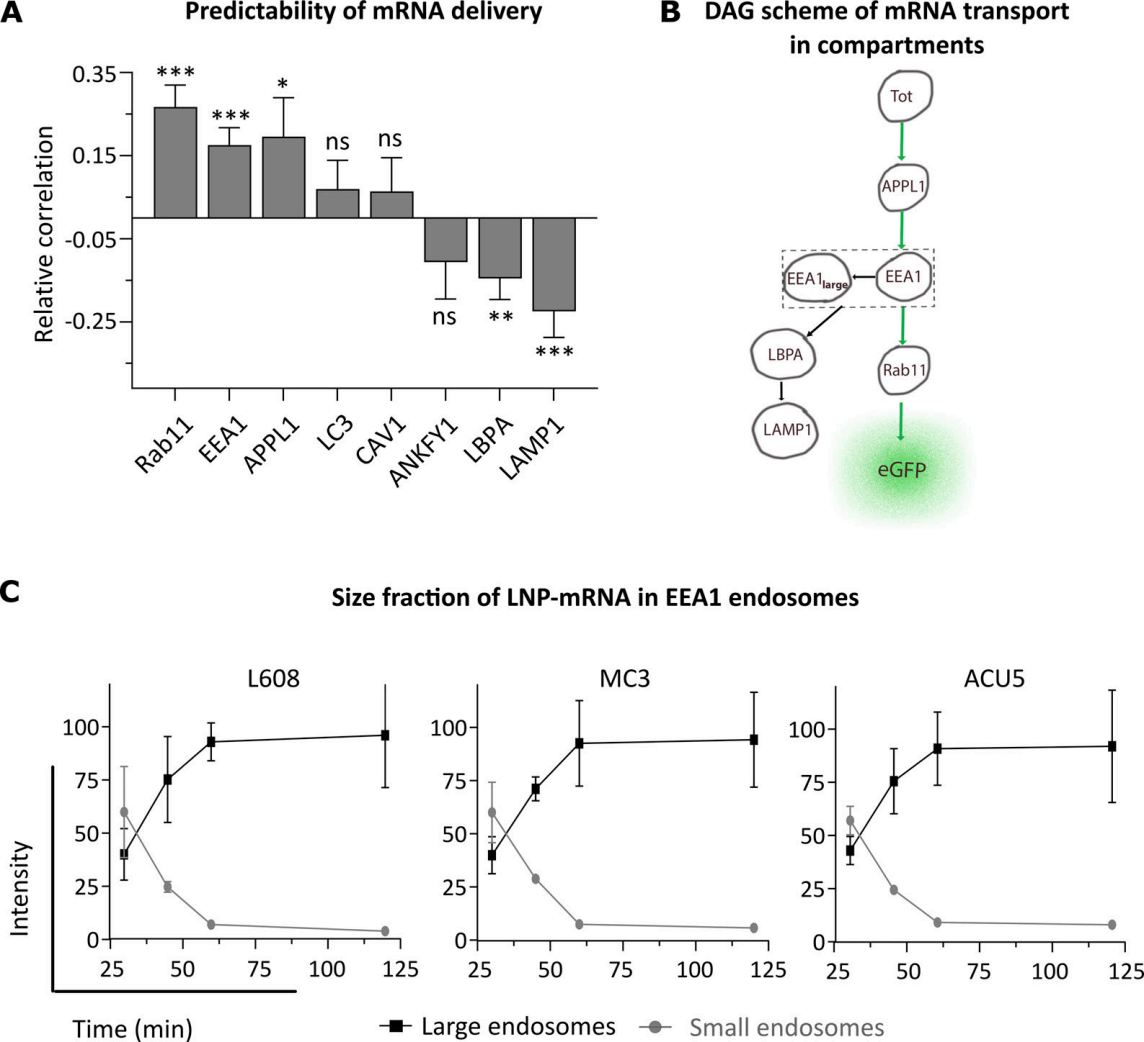
**Differential correlation and size distribution kinetics analysis reveal that Rab11^+^, small EEA1^+^, and APPL1^+^ endosomes are more favorable than other endosomal compartments for mRNA delivery.** **(A)** The graph shows the differential correlation between LNP-mRNA (smFISH-labeled) distribution in eight endosomal compartments and eGFP expression in human primary adipocytes (see Materials and methods). Three compartments, Rab11, EEA1, and APPL1, have a correlation above total uptake and, therefore, are potential hot spots of mRNA escape, whereas, e.g., Lamp1 is not. *n* = 5 independent experiments for Rab11, EEA1, APPL1, and LBPA and *n* = 3 independent experiments for the other markers. *, P < 0.05; **, P < 0.01; ***, P < 0.001 by two-sided *t* test. **(B)** Schematic representation of LNP-mRNA transport through endosomal compartments reconstructed based on the DAG differential correlation (A) and size fraction analysis (C). The total LNP-mRNA uptake is represented by node Tot, which is upstream of the various endosomal compartments (represented by the indicated nodes) where cargo is sequentially distributed to. The Rab11 node is located close to the node eGFP because of its high correlation of LNP-mRNA distribution with eGFP expression (see Materials and methods). Whereas the node EEA1 is upstream because it consists of small EEA1 LNP-mRNA endosomes that are productive for delivery, the large arrested (EEA1_large_) endosomes are less/unproductive for LNP-mRNA delivery. **(C)** Quantification of LNP-mRNA accumulation in EEA1 endosomes based on endosome size and intensity. The graph illustrates that the fraction of large LNP-mRNA endosomes (object diameter >1 μm) increases substantially, whereas the smaller ones (object diameter = up to 0.75 μm) decrease over time. Decrease of small LNP-mRNA endosomes: L608 = 60.0 ± 14% to 5.7 ± 0.4; MC3 = 59.9 ± 21% to 4 ± 0.5%; ACU5 = 57 ± 7% to 8 ± 1.0%. Increase of large LNP-mRNA endosomes: L608 = 40.0 ± 9% to 94.2 ± 22%; MC3 = 40.0 ± 12% to 96.0 ± 24%; ACU5 = 42.9 ± 7% to 92.3 ± 26%. *n* = 3 replicates (mean ± SEM).

To rank the compartments with the highest probability of escape, we used a directed acyclic graph (DAG), a general mathematical tool to infer dependencies between observed variables by differential correlation (Supplemental figures, Fig. S3; see Materials and methods). We applied DAG to the sequential transport of LNP-mRNA through the compartments of the endocytic system, from internalization to mRNA escape, to infer the correlation between amount of LNP-mRNA in a compartment and escape. Of the eight compartments tested, only the EEA1, APPL1, and Rab11 compartments had positive differential correlations (Fig. 2, A and B; see Materials and methods). This suggests that in the path from uptake to escape, mRNA sequentially traverses APPL1^+^, EEA1^+^, and Rab11^+^ compartments, with Rab11 endosomes having the highest probability for mRNA escape (Fig. 2 B and Supplemental figures, Fig. S3; see Materials and methods). This sequence is consistent with previous measurements of cargo flux through the endosomal system (Jovic et al., 2010; Kalaidzidis et al., 2015a). LBPA^+^ (multivesicular bodies, late endosomes), LAMP1^+^ (late endosomes and lysosomes), and LC3^+^ (autophagosomes) compartments have a negative or zero differential correlation (Fig. 2 A) and, thus, are unlikely to promote endosomal escape.

EEA1^+^ early endosomes constitute a heterogeneous population that gradually increases in size as they accumulate degradative cargo until they convert into late endosomes (Rink et al., 2005). The small-sized early endosomes actively sort recycling cargo through Rab11 endosomes, whereas the large ones assume characteristics of multivesicular bodies enriched in LBPA (Gruenberg, 2020). Given the low correlation of LBPA to delivery (Fig. 2 A), we hypothesized that the small EEA1 endosomes are more competent for delivery than the large ones. Therefore, we analyzed EEA1 endosomes with respect to mRNA delivery dependent on size. The fraction of endosomes with diameter >1.0 μm (Fig. 2 B, denoted as EEA1_large_) increased over time at the expense of the fraction with diameter <0.75 μm (EEA1_small_) in L608, MC3, and ACU5 LNPs (Fig. 2 C). Note that accumulation of LNP-mRNA in large-sized endosomes is not due to saturation of the endosomal system, as only <5% of EEA1 endosomes contained LNP-mRNA during the initial 2 h of uptake (Supplemental figures, Fig. S4). The finding that LNP-mRNA accumulate (∼90% at 2 h) in few (<5%) large early endosomes without converting into late endosomes suggests that progression of cargo is inhibited. However, since such accumulation did not correlate with eGFP expression, we conclude that these compartments contribute minimally or not at all to delivery.

### LNPs differentially impair endosomal acidification, leading to mRNA accumulation in nonproductive compartments

**Figure 3.**
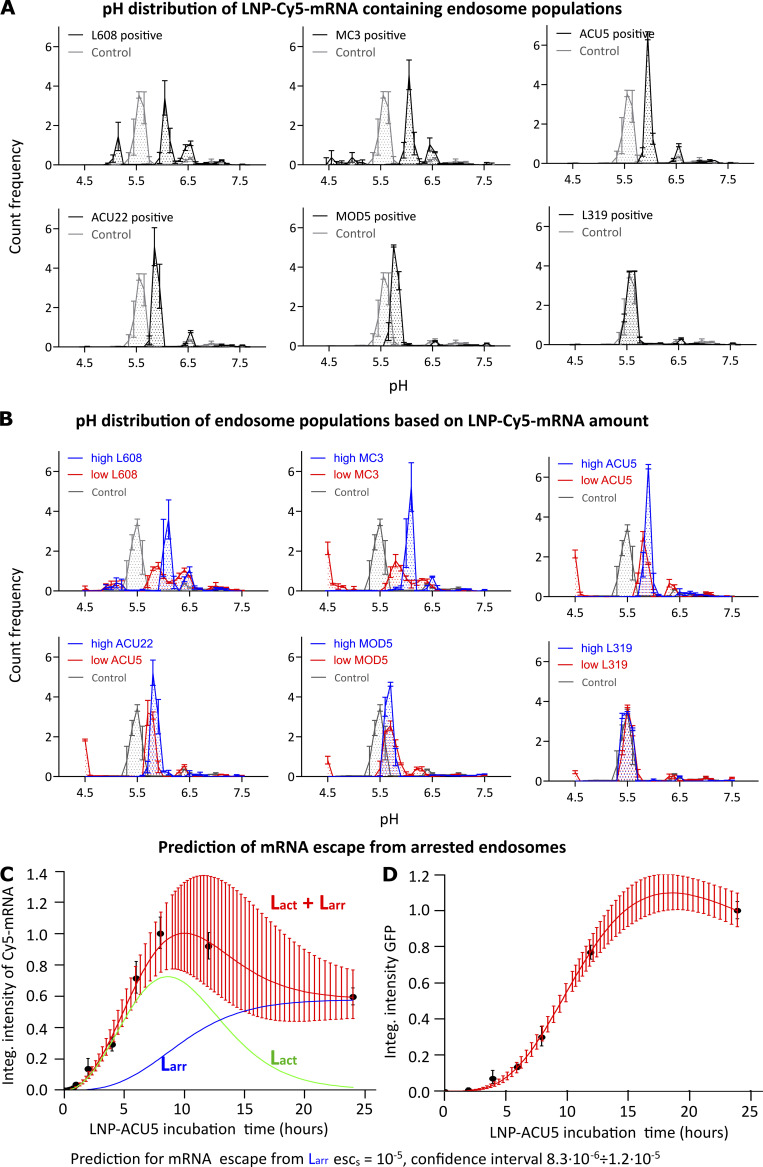
**Endosomes accumulating LNP-Cy5-mRNA display acidification defects and negligible endosomal mRNA escape. (A)** HeLa cells were incubated with pH Rodo-Red and pH stable Alexa Fluor 488 fluorophore-labeled LDLs with and without LNP-Cy5-mRNA for 2 h and imaged live. The pH of LNP-Cy5-mRNA–containing endosomes was calculated by intensity ratiometric analysis from LDL-pH Rodo-Red and LDL-Alexa Fluor 488 (see Materials and methods). Whereas in control cells (LDL probes internalized without LNP-Cy5-mRNA, gray line), a significant proportion of endosomes was acidified to pH 5.5, characteristic of late endosomes/lysosomes, most LNP-Cy5-mRNA–containing endosomes (black line) stalled between early (pH 6.5) and late endosomal (pH 5.5) pH. **(B)** The graph illustrates pH distribution in endosome populations based on their LNP-Cy5-mRNA accumulation (high versus low). Endosomes with a high amount of LNP-Cy5-mRNA show higher pH (blue line, prominent in L608 and MC3, less prominent in ACU5 and ACU22, no difference in MOD5 and L319) than those with low LNP-Cy5-mRNA (red line). **(C and D)** Mathematical model for prediction of mRNA escape from arrested (L_arr_) endosomes in human primary adipocytes. The theoretical model fit (red lines in C and D) to experimental ACU5 LNP-Cy5-mRNA uptake kinetics (C, black dots) and GFP expression (D, black dots). Green and blue lines denote model predictions for active (L_act_) and L_arr_ endosomes, respectively (see Materials and methods; Supplemental figures, Fig. S5, A and B). Experimental points and error bars in A and B indicate mean ± SEM of three independent experiments. The error bars in C and D represent the 95% confidence interval on the theoretical curves (red) estimated by uncertainty distribution approximation by normal distribution with inverse Hessian of likelihood as a covariance matrix. Integ., integral.

A possible mechanism blocking early endosome maturation is suppression of endosomal acidification (Mauthe et al., 2018; Novikoff et al., 1956; Yoshimori et al., 1991). Therefore, to measure the pH of endosomes, we used ratiometric intensity measurements (Maxfield and Yamashiro, 1987) of low-density lipoprotein (LDL) conjugated to pH-sensitive Rodo-Red and Alexa Fluor 488 probes cointernalized with LNP-cyanine 5 (Cy5)-mRNA for 45, 120, and 180 min and imaged in living cells (see Materials and methods). Since we aimed to achieve high accuracy of the pH measurements, we applied the well-established procedure of deconvolution to the analysis of ratio distributions (see Materials and methods). The excessive amount of autofluorescence precluded the possibility to make such measurements in adipocytes. Therefore, we turned to HeLa cells as a widely used cell system. We first verified that HeLa cells could be transfected with LNP-mRNA (Supplemental figures, Fig. S5, A and B). Although eGFP expression in HeLa cells and adipocytes is significantly different, the correlations between total LNP-mRNA uptake and eGFP expression (HeLa: *r* = 0.94; adipocytes: *r* = 0.83) are comparable. This validated the choice of HeLa cells as a substitute for adipocytes to study LNP influence on endosomal lumen pH. In control cells that cointernalized the LDL probes (by receptor-mediated endocytosis) for 120 min without LNP-mRNA, 5% of LDL^+^ structures had a pH of 6.5 and 74% a pH of 5.5, characteristic of early and late endosomes, respectively (Fig. 3 A and Table S2; Maxfield and Yamashiro, 1987). In contrast, a major fraction of LNP-Cy5-mRNA (except for L319) containing endosomes had higher pH values (between early and late endosomes), i.e., failed to acidify to late endosomal pH, corroborating the hypothesis of endosomal maturation arrest. The percentage of arrested endosomes continued to increase over time (Table S3). Interestingly, LNP formulations differed profoundly in their effects on endosomal acidification (Fig. 3 A). The poorly endocytosed L319 LNP-Cy5-mRNA did not significantly affect endosomal acidification.

Next, we hypothesized that endosomes accumulating large amount of LNP-Cy5-mRNA are more severely blocked in acidification than endosomes accumulating less. Therefore, we analyzed the pH of endosomes with varying degrees of LNP-Cy5-mRNA accumulation (upper tertile versus lower tertile) for each formulation. As predicted, endosome populations with high LNP-Cy5-mRNA showed higher pH than their counterparts for many LNP formulations (Fig. 3 B). In control conditions, LDL was undetectable in compartments with pH <5, i.e., the characteristic pH of lysosomes, indicating that it is degraded in lysosomes. Interestingly, in endosomes with high LNP-Cy5-mRNA content, we never detected pH <5.5, indicating a block of acidification. In contrast, in all LNP-treated cells, LDL was found in a subpopulation of vesicles with low LNP-Cy5-mRNA content and pH <5. This suggests that high LNP accumulation causes a block of endosomal acidification, whereas low LNP accumulation, despite not severely blocking acidification, inhibits cargo degradation.

Impaired acidification and/or cargo degradation may also cause failure to recycle LDL receptor (LDLR) to the plasma membrane, thus reducing LDL uptake. Consistent with this hypothesis, cells incubated with LNP-Cy5-mRNA, which impairs endosomal acidification, also showed poor LDL uptake compared with the control- and L319 LNP-Cy5-mRNA–incubated cells (Supplemental figures, Figs. S6 and S7).

If endosomes are impaired in cargo progression (Fig. 2 C and Supplemental figures, Fig. S4), acidification, and cargo uptake, they are probably also unproductive for mRNA escape. A simple model and experimental data of LNP propagation through endocytic compartments provided support for this idea (Supplemental figures, Fig. S8 A; see Materials and methods). In this model, we considered two groups of endosomes: “active” and “arrested”. Besides the eGFP degradation rate (Balleza et al., 2018), all parameters were deduced by fitting model predictions (Fig. 3 B, lines) to experimentally measured intensity values of ACU5 LNP-Cy5-mRNA in endosomes (Fig. 3 B, black dots) and expressed eGFP (Fig. 3 C, black dots; see Materials and methods). The best fit to experimental data predicted that escape from the arrested endosomes is negligible (Supplemental figures, Fig. S8 B; esc_S_ = 10^−5^; 95% confidence interval, 8.3 ⋅ 10^−6^ ÷ 1.2 ⋅ 10^−5^), indeed suggesting that they do not contribute significantly to mRNA delivery.

### Multicolor single-molecule localization microscopy resolves singular LNP in endosomes with nanometer resolution

Rab11^+^, APPL1^+^, and (nonarrested) EEA1^+^ endosomes share tubular structures transporting recycling cargo, such as transferrin, to the plasma membrane (Franke et al., 2019; Kalaidzidis et al., 2015a; Sönnichsen et al., 2000; Ullrich et al., 1996). We wondered whether such recycling tubules may be preferential sites of mRNA escape into the cytosol. Conventional light microscopy cannot provide sufficient resolution to visualize mRNAs within endosomes or in the cytoplasm. Therefore, we used multicolor single-molecule localization microscopy (SMLM; Franke et al., 2019). The number of mRNA molecules per LNP ranged between 5 and 25 (Sabnis et al., 2018; Yanez Arteta et al., 2018), and each mRNA contained multiple Cy5 (average, ∼25 per mRNA, L-7701; TriLink Bio Technologies; see Materials and methods), permitting the resolution of the geometry of single LNP and even single Cy5-mRNA, i.e., stretched (maximum length of 996 nt ≈ 300 nm, L-7701; TriLink Bio Technologies) versus condensed. We first benchmarked our SMLM by visualizing isolated LNPs rested on a glass surface at the nanoscale (see Materials and methods). Individual LNPs were resolved as isolated clusters of single-molecule spots with median diameter ∼60 nm (Supplemental figures, Fig. S9). Smaller elongated clusters (∼25-nm diameter) may correspond to damaged LNP or even dissociated Cy5-mRNAs (Supplemental figures, Fig. S9).

**Figure 4.**
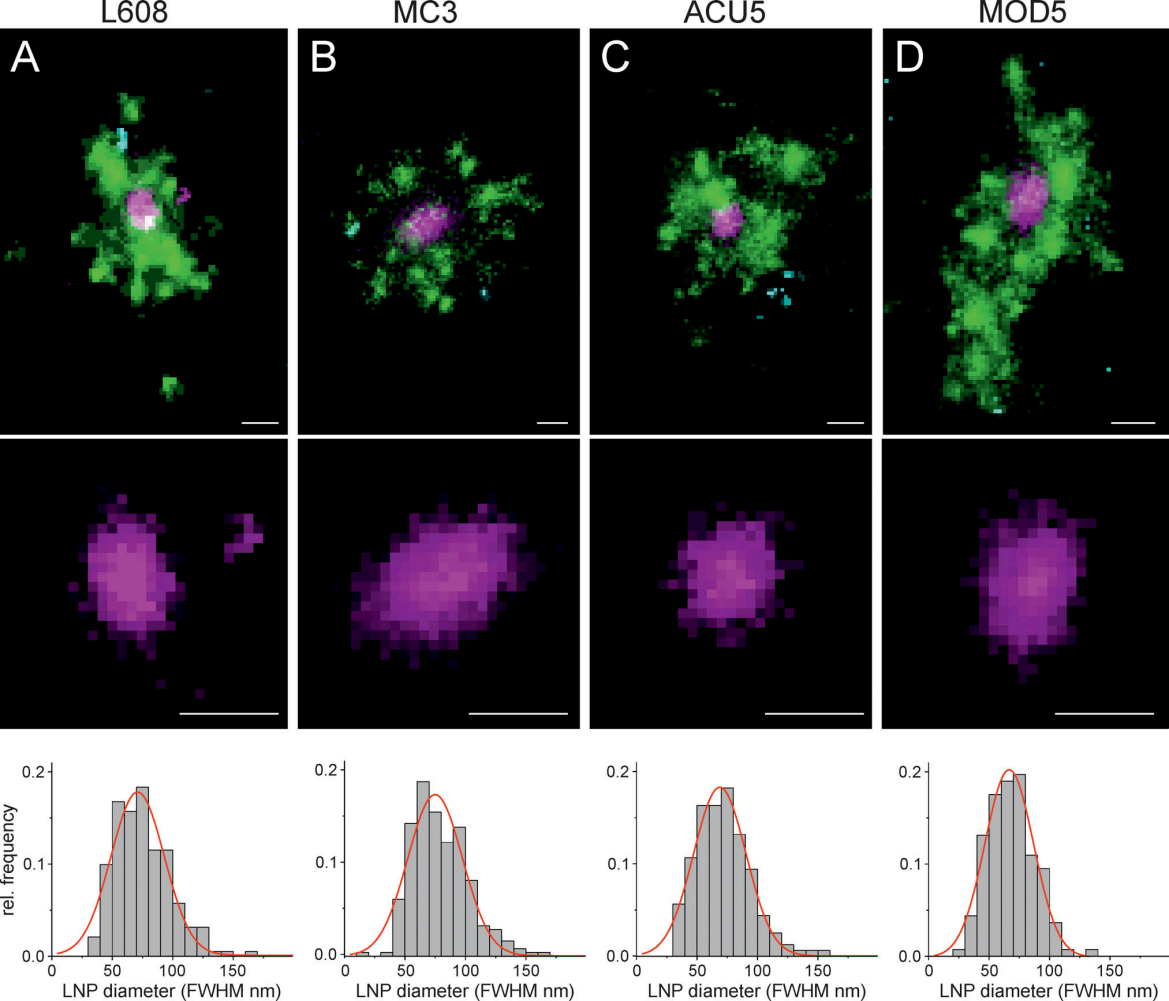
**Multicolor SMLM detects and visualizes singular LNP-mRNA in endosomal compartments with nanometer resolution. (A–D)** Exemplary images of endosomes containing a single, clearly detectable LNP-Cy5-mRNA (magenta) together with transferrin (green) and EGF (cyan) cargo for each imaged LNP formulation (top row). SMLM resolves single LNP as round nano domains (zoom-in; middle row) with characteristic size distribution. LNP diameter distributions were built by determining the FWHM of single LNPs (see Materials and methods). Mean LNP diameters (FWHM) were calculated by Gaussian fitting of the distributions to (mean ± SD) 74.9 ± 22.6, 71.0 ± 22.0, 66.7 ± 20.2, and 68.9 ± 21.7 nm, respectively. The cellular context of displayed endosomes is provided in Supplemental figures, Figs. S10, S25, S26, and S27. Scale bars are 100 nm. rel., relative.

Next, we performed SMLM on cells. In adipocytes, the large lipid droplets function as microlenses that distort the single-molecule emission patterns. Therefore, we first performed SMLM in HeLa cells on the entire cell and then validated the observations in human primary adipocytes by focusing on the endosomes underneath the plasma membrane by total internal reflection fluorescence. As we aimed to resolve mRNA localization within endosomes versus cytoplasm, we refrained from using immunostaining, which requires membrane permeabilization and may cause membrane leakage artifacts. We used receptor-mediated uptake of transferrin (Alexa Fluor 568) and EGF (Alexa Fluor 488) to label early-recycling and early-late endosomes, respectively (Franke et al., 2019). We compared four LNP formulations with high, middle, and low (L608, MC3, ACU5, and MOD5) eGFP expression. Strikingly, we could resolve individual LNPs within endosomes labeled by EGF and/or transferrin both in HeLa cells and in primary human adipocytes (Fig. 4). Interestingly, singular LNPs were most often associated with transferrin-positive structures, i.e., within early-recycling compartments. To our knowledge, this is the first time that LNPs are resolved by SMLM in subendosomal compartments. Based on our previous correlative ultrastructural analysis of early endosomes (Franke et al., 2019), closely located clusters of single-molecule signals from labeled cargo belong to a single endosome (see Materials and methods). Therefore, we quantified the size distribution of single LNPs within endosomes. The size was determined as a full width at half maximum (FWHM) of intensity (see Materials and methods). The calculated LNP diameters (Fig. 4) are in good agreement with measurements of LNP size by dynamic light scattering (DLS; Table S1) and SMLM on a glass surface (Supplemental figures, Fig. S9). Therefore, SMLM allows resolution of individual LNPs with subendosomal precision.

### LNPs accumulate in arrested endosomes, often lacking internalized EGF and transferrin

**Figure 5.**
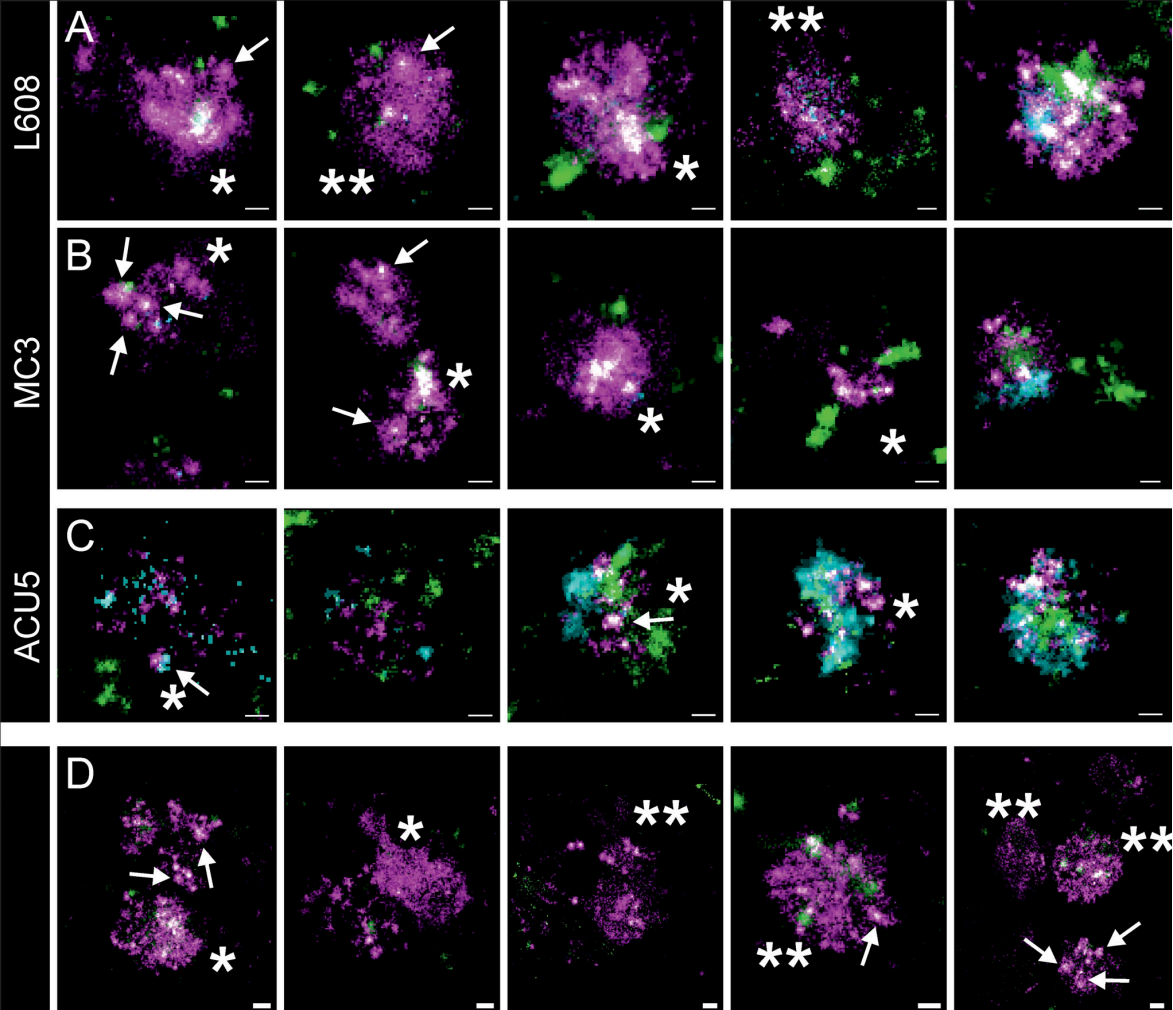
**Arrested endosomes are large structures filled with dense and dispersed mRNA and often devoid of endocytosed cargo. (A–C)** SMLM visualizes large, LNP-Cy5-mRNA–rich (magenta) structures in HeLa cells incubated with L608 (A), MC3 (B), and ACU5 (C) LNPs. These endosomes can display an accumulation of LNP-Cy5-mRNA–like puncta (likely to be single LNPs indicated with arrows; single asterisk) but more often a dispersed signal over large areas (double asterisks). Most endosomes that exhibit these characteristics show a substantial lack of transferrin (green) and EGF (cyan) cargo compared with endosomes containing singular LNPs (compare Fig. 3), although endosomes with varying degrees of cargo content together with a strong mRNA signal also can be found. **(A and B)** Increasing cargo content from left to right. **(C)** In the case of ACU5, large endosomal structures, also containing more than one LNP, can be found. Contrary to A and B, cells incubated with ACU5 often exhibited endosomes with cargo, especially EGF. **(D)** Arrested endosomes with similar features were consistently found in primary human adipocytes. ROIs indicating the cellular context of displayed endosomes are provided in Supplemental figures, Figs. S10, S11, S12, S13, S14, S15, S16, S17, S18, and S19. Scale bars are 100 nm.

For L608 and MC3, we consistently found a significant number of larger structures with an abundance of Cy5-mRNA signal in HeLa cells, primary human adipocytes, and fibroblasts in the same culture (Fig. 5, Supplemental figures, Figs. S10, S11, S12, S13, S14, S15, S16, S17, S18, S19, and S29). Within these structures, in some cases, individual LNPs could be resolved (Fig. 5, arrows). We also detected large Cy5-mRNA^+^ structures that either corresponded to LNPs too close to be resolved (Fig. 5, single asterisk) or dispersed Cy5-mRNA (Fig. 5, double asterisks). This suggests that some LNPs are disassembled and the mRNAs released into the lumen of the endosomes. Interestingly, most of these large endosomes filled with LNP-mRNA exhibited little EGF/transferrin signal, in contrast to endosomes having no LNP-mRNA, or small endosomes with a single LNP-mRNA that contained internalized EGF/transferrin (Fig. 5 and Supplemental figures, Figs. S10, S11, S12, S13, S14, S15, S16, S17, S18, and S19). These observations corroborate the pH measurement data (Fig. 3 A), suggesting that a significant fraction of endosomal structures with accumulated LNPs is in a maturation-arrested state and insulated from normal cargo uptake (Supplemental figures, Figs. S6 and S7).

### Multicolor SMLM reveals mRNA escape from transferrin-containing endosomal tubules

**Figure 6.**
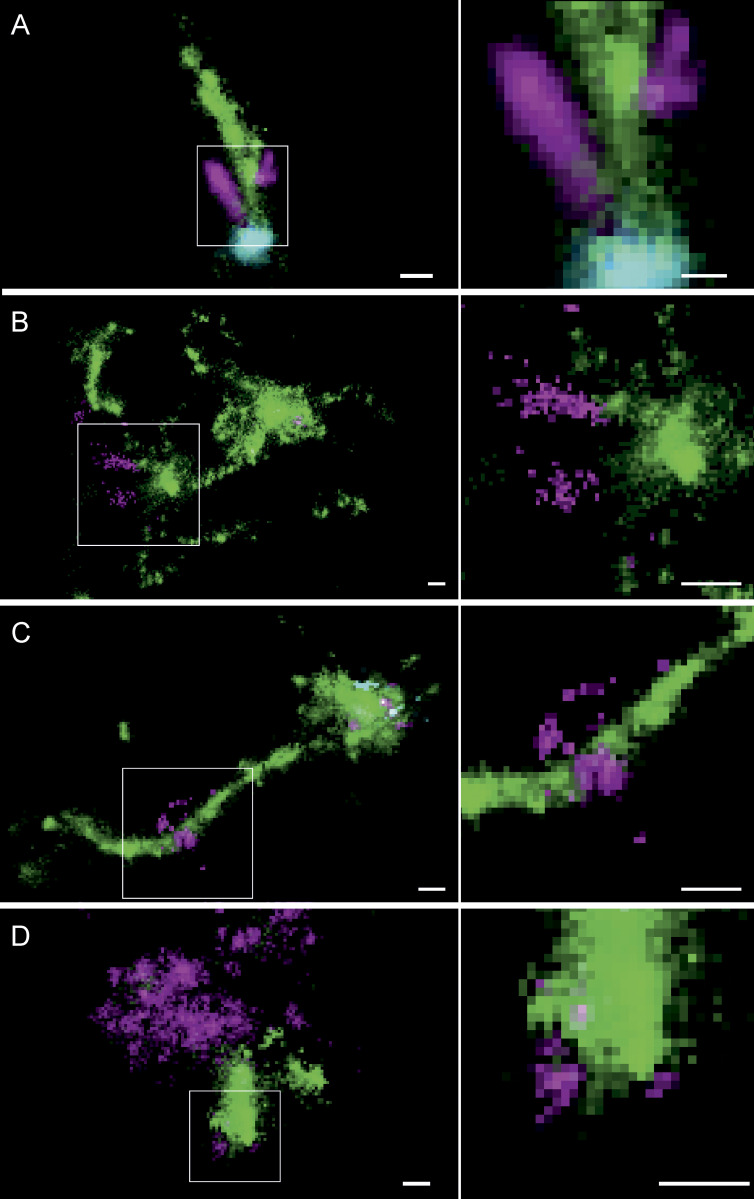
**Multicolor SMLM suggests endosomal mRNA escape at the nanoscale from transferrin-containing tubules in primary human adipocytes and HeLa cells.** Exemplary images of the different observable types of Cy5-mRNA (magenta) escape in primary human adipocytes and HeLa cells relative to transferrin-positive tubules (green) and EGF (cyan). **(A)** Concentrated mRNA signal is located at the very tip of a transferrin-positive tubule, connected to an elongated mRNA signal colocalizing along the tubule. Very sparse mRNA signal can be detected outside the tubule. **(B)** Disperse Cy5-mRNA is seemingly emanating from the transferrin-positive tubule from which it is already segregated. These patterns clearly do not constitute intact LNP and are likely to represent partly stretched mRNA molecules escaping from endosomal structures. **(C)** An LNP is located on a long transferrin-positive tubule together with a perpendicular dispersed Cy5-mRNA signal, likely representing an instance of Cy5-mRNA escape. **(D)** In very rare cases, also in arrested endosomes (compare Fig. 4), dispersed mRNA signal attached to a transferrin-positive structure can be detected. ROIs indicating the cellular context of displayed endosomes are provided in Supplemental figures, Figs. S15, S17, S18, S19, S20, S21, S22, S23, S24, S25, and S26. Zoom-in of the indicated regions are presented in the right-side panels. Scale bars are 100 nm.

In many cases, we found that Cy5-mRNA distinctly localized to transferrin-positive tubules for all four LNP formulations (Fig. 6 and Supplemental figures, Figs. S15, S17, S18, S19, S20, S21, S22, S23, S24, S25, and S26). In the majority of such cases, the Cy5-mRNA signal could be observed alongside or at the very tip of tubules (Fig. 6 A). Interestingly, we could also detect Cy5-mRNA signal that was not as intense and condensed as the single LNP shown in Fig. 4 or as large diffuse agglomerates as in Fig. 5 but formed a pattern ranging from clustered to dispersed single-molecule flashes (Fig. 6 B). Importantly, such a signal was adjacent to, or outside of, transferrin-positive structures, suggesting that this may correspond to single or few unpacked mRNAs escaped or in the process of escaping (Fig. 6 B). Strikingly, in very rare cases, we could resolve Cy5 flashes ordered along a smooth line spanning a tubule and projecting into the cytoplasm (Fig. 6 C). We interpret these events as capturing the escape of the mRNA from endosomal tubules. This interpretation is supported by several lines of evidence. First, most mRNA signals have the nanoscopic appearance of single LNPs (Fig. 4) and are associated with endosomal structures easily recognizable by the presence of EGF/transferrin. Only a small fraction of Cy5-mRNA did not colocalize with, or was in close proximity to, endosomal markers. Such a signal was structurally very different from the Cy5-mRNA entangled within endosomes (Figs. 4 and 5), and notably, its proportion varied depending on the formulation. The frequency of signal attributed to outside of endosomes was (mean ± SD) 6.9% ± 1.5 (L608), 5.9% ± 1.3 (MC3), 4.0% ± 0.7 (MOD5), and 3.0% ± 0.9 (ACU5). Therefore, the signal interpreted as cytoplasmic correlated well (*r* = 0.85) with eGFP expression (see Materials and methods). This is a compelling statistical argument supporting the conclusion that, if not all, at least the majority of cytoplasmic mRNA detected in the images correspond to escaped events. Second, it is unlikely that this signal may be due to rupture of the endosomal membrane due to fixation. The SMLM protocol used does not detectably alter the integrity of the endosomal membranes (limiting membrane, intralumenal vesicles, and recycling tubules) at the ultrastructural level (Franke et al., 2019). However, if there was significant membrane rupture (endosomal as well as plasma membrane), we would expect to detect cytoplasmic mRNA signal, i.e., not colocalized with EGF/transferrin, also at early time points. This is not the case, as after 30-min uptake of LNPs, no escaped mRNA was detected but only single LNPs were visible within endosomes (Supplemental figures, Fig. S28). We cannot obviously exclude that at least a fraction of mRNA signal that does not colocalize with EGF/transferrin could still be within unlabeled endosomal compartments. However, this is unlikely because such a compartment would have to be an early compartment (no high accumulation of LNP-mRNA), and yet, it is not detected in the 30-min time point. Third, the combination of the HiLo illumination and the restriction of the fitted FWHM results in a reasonably narrow axial distribution of mRNA localizations in our SMLM images (Supplemental figures, Fig. S30). Therefore, a random axial colocalization of distinct Cy5-mRNA signal to transferrin tubules can be excluded with great certainty. Fourth, the observed patterns did not change during the time of acquisition, ruling out sample drift or diffusion artifacts (Supplemental figures, Fig. S31). Fifth, escape events could also be detected at endosomes containing multiple LNPs and transferrin, i.e., competent for cargo transport. The Cy5 signal resembling stretched mRNA molecules was exclusively associated with the transferrin tubules and nowhere else at the object perimeter (Fig. 6 D). Finally, we consistently found similar events in primary human adipocytes and HeLa cells (Fig. 6 and Supplemental figures, Figs. S15, S17, S18, S19, S20, S21, S22, S23, S24, S25, and S26), substantiating the notion that mRNA escape from transferrin-positive tubules is a general mechanism contributing to effective transfection and shared by different cell types.

## Discussion

The biggest challenge for the delivery of mRNA, and macromolecular therapeutics in general, is to target them to the correct cells and, once endocytosed, let them cross the endosomal membrane. Only a small fraction of exogenous macromolecules can escape from endosomes via yet-unknown mechanisms. To gain insight into this outstanding problem, we performed a comparative analysis of six LNPs with distinct chemical composition and delivery efficiency in primary human adipocytes, fibroblasts, and HeLa cells to identify endosomal compartments that are most favorable to mRNA escape. Contrary to what is generally assumed (Novakowski et al., 2019; Pei and Buyanova, 2019), our analysis revealed that delivery efficacy cannot be predicted by total cellular uptake alone. LNPs had different uptake efficiencies and, most importantly, different endosomal distributions. We found that a subpopulation of EEA1^+^, APPL1^+^, and Rab11^+^ early/recycling endosomes have a higher probability for mRNA escape than late endosomes (i.e., LBPA, LAMP1). Therefore, our data contrast both claims that RNA escape does not occur from EEA1 endosomes (Wittrup et al., 2015) and that LNP recycling is counterproductive for delivery (Sahay et al., 2013).

**Figure 7.**
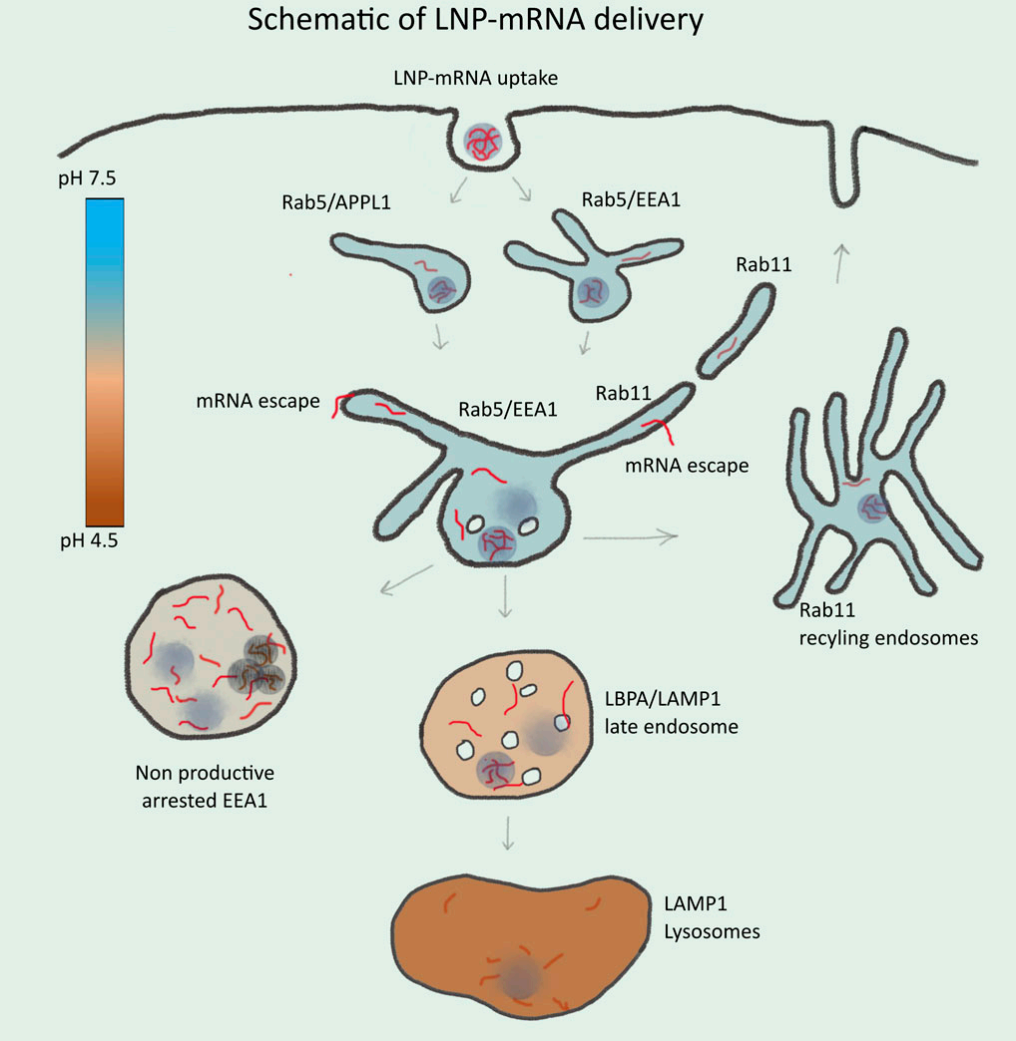
**Schematic illustration of endosomal-mediated LNP-mRNA delivery.** LNP-mRNAs are taken up in cells via endocytosis and sequentially transported to various endosomal compartments. Under normal conditions, these endosomal compartments maintain a characteristic pH (see heat map) for their functionality. Following uptake, mRNA in endosomes can be detected as individual LNP-mRNAs similar to the starting material by SMLM. Acidification of endosomal lumen leads to release of mRNA from LNP and escape from the endosomal lumen into the cytoplasm. Escape occurs mainly from small APPL1^+^, EEA1^+^, and/or Rab11^+^ tubular endosomes. Over time, the majority of LNP-mRNA accumulates in large, EEA1 endosomes where individual compact LNPs are disrupted, and mRNA signal becomes amorphous. Large endosomes become deficient in acidification, maturation arrested, and nonproductive for delivery. This may account for cytotoxicity of LNPs. Late endosomes and lysosomes are not favorable for mRNA escape.

The endosomal compartments that correlate best with delivery efficacy (EEA1, APPL1, and Rab11) share a high proportion of recycling tubules (Kalaidzidis et al., 2015a; Sönnichsen et al., 2000). The tubulation of endosomal compartments in dendritic cells (Nakamura et al., 2014) may underlie the efficacy of mRNA-based vaccines. Consistent with this interpretation, by SMLM, we could capture rare events of single mRNA molecules in the process of escaping from transferrin-containing tubules in primary human adipocytes and HeLa cells. What makes endocytic/recycling endosomes particularly favorable to macromolecule escape? Multiple potential mechanisms have been proposed, such as proton sponge and subsequent endosomal bursting, cationic lipid-based membrane destabilization, and pore formation-mediated membrane fusion (Pei and Buyanova, 2019). However, none of these mechanisms have received compelling experimental support, and endosome bursting appears restricted to lipoplexes but not to LNPs (Gilleron et al., 2013; Wittrup et al., 2015). It is possible that multiple mechanisms are in play, depending on differences in LNP composition and surface properties, macromolecules, and their transport. Our data argue for a new mechanism. Endosomal recycling tubules are characterized by high positive curvature along the tubules and sharp transition to negative curvature at the neck of the tubules. Exogenous cationic lipids may interfere with the packing of lipids in the membrane bilayer, resulting in local instability and, thus, membrane leakage. In addition, recycling tubule fission could create spontaneous breakage of the membrane favoring macromolecular escape. Therefore, we propose that endosomes with recycling tubules are hot spots for mRNA escape events (Fig. 7).

For some LNP-mRNA formulations, a large fraction accumulates in a small population of early endosomes that are defective in new cargo uptake and arrested in maturation. We found these structures consistently in adipocytes, fibroblasts, and HeLa cells, suggesting that this is a common defect. By SMLM, we could resolve mRNA packed in single LNPs and in an unpacked form within these endosomes. The unpacking probably depends on lipid reorganization at low pH that facilitates release of mRNA from LNP into the endosomal lumen. Despite the abundance of LNP-mRNA in the enlarged endosomes, we never detected mRNA escape events associated with them. We interpret these endosomes as incompetent for mRNA delivery, in agreement with the correlation analysis. In view of these considerations, the claim that enhanced retention of LNP can result in productive RNA escape (Sahay et al., 2013) is untenable. The accumulation of undeliverable LNP-mRNA in endosomes does not support the hypothesis of endosome disruption by the proton sponge mechanism and argues that retarding cargo degradation along the endosomal pathway may not increase the chance for cargo escape but, rather, may contribute to toxic effects. An important finding was that such LNP accumulation is accompanied by endosome acidification defects. Interestingly, MOD5 (an analogue of the lipid used in mRNA-1273 SARS-CoV-2 vaccine) also had an impact on acidification, albeit less than MC3. One potential reason could be protonation of ionizable cationic lipids in the formulation that may produce buffering effects similar to the proton sponge mechanism (Pei and Buyanova, 2019). Such a defect cannot persist over time if the V-ATPase maintaining the pH of endosomes remains functional. However, ionizable LNP lipids may interfere with V-ATPase activity (Jones et al., 1995) or increase the leakiness of endosomal membrane to protons. If the endosomal pH is greater than the pH required for LNP lipid reorganization, the unpacking of LNPs and release of mRNA within the endosomal lumen will fail. In addition, acidification is so critical to various endosomal activities, such as protein sorting, endosomal progression, lysosomal degradation, and cellular homeostasis, that if compromised, will lead to a series of cytotoxic consequences. As endolysosomal hydrolytic enzymes require acidic pH (Dubland and Francis, 2015), suppressed acidification will prevent the biodegradation of the LNP lipids (per se biodegradable), thus exacerbating LNP accumulation further. LNP uptake is typically mediated by LDLR (Akinc et al., 2010). Lack of acidification will impede LDL–LDLR dissociation, preventing LDLR from recycling to the surface (Bai et al., 2018; Davis et al., 1987) and thus blocking further uptake. This is consistent with the decreased LNP uptake at later times of internalization (Fig. 1 B). Interestingly, we also observed inhibition of LDL degradation in endosomes where LNP accumulated (Fig. 3 B). Endosomal maturation arrest and accumulation of undegraded cargo are reminiscent of lysosomal storage disorders (Ballabio and Gieselmann, 2009; Platt et al., 2012). In addition to cytotoxicity, these alterations may cause an inflammatory response similar to the immune system defects characterizing lysosomal storage disorders (Castaneda et al., 2008). Therefore, defective endosomal acidification may account for a great deal of the cytotoxic effects of LNPs (Garber, 2017; Kulkarni et al., 2018).

Our results define quantitative endosomal parameters that can guide the development of new mRNA formulations toward high efficacy and low cytotoxicity by breaking down the process of delivery into at least three distinct stages. The first step to optimize is clearly the uptake of LNP, which is mediated by apolipoprotein E (Akinc et al., 2010) and, therefore, depends on the binding of apolipoprotein E to LNP. The second stage is the dissociation of LNP and mRNA within the early endosomes, a process that can be monitored at the nanoscale (Figs. 4 and 5). A parameter to monitor is the potential source of cytotoxicity by suppression of endosome acidification. The third stage is the escape of mRNA, which depends on the fraction that accumulates in recycling tubules. A possible strategy would be to develop LNPs that can distribute more evenly between endosomes or even preferentially sorted to recycling tubules. For this, extended binding to LDLR may prolong the resident time in recycling endosomes, increasing efficacy and decreasing toxicity. We suggest the importance of performing a structure/function relationship analysis of different LNP components, e.g., structure of lipid heads and tails, not only with respect to the different delivery stages independently of each other but also with respect to the cytotoxic effects derived from the block of acidification. The assays and parameters to measure them, as described in this study, can therefore complement the chemical optimization of delivery systems.

## Materials and methods

### Cell culture

Human adipose stem cells (hASCs) were obtained from patients undergoing elective surgery at Sahlgrenska University Hospital in Gothenburg, Sweden, all of whom received written and oral information before giving written informed consent for the use of the tissue. The studies were approved by the regional ethical review board in Gothenburg, Sweden. All procedures performed in studies involving human participants were in accordance with the ethical standards of the institutional and national research committee and with the 1964 Declaration of Helsinki and its later amendments or comparable ethical standards. All subjects complied with ethical regulations. hASCs tested negative for mycoplasma. We adapted a previously published protocol (Bartesaghi et al., 2015) from AstraZeneca to differentiate hASCs to mature white-like adipocytes in 384-well format. Briefly, EGM-2 proliferation medium was prepared according to the manufacture’s protocol with EBM-2 medium supplemented with 5% FBS and all provided supplements, except hydrocortisone and GA-1000 (EGM-2MV Bulletkit [CC-3156 and CC-41472], cat. no. 3202; Lonza). Cryopreserved hASCs were then resuspended in EGM-2 medium and centrifuged at 200 ×*g* for 5 min. Cells were counted with a CASY cell counter (Schärfe System), and 4,000 cells/well were seeded in 50 μl EGM-2 medium containing 50 U/ml penicillin and 50 μg/ml streptomycin (P/S; 15140-122; Gibco-BRL) into 384-well plates (781092; Greiner Bio-One) using the Multidrop dispenser (Thermo Fisher Scientific). The cells were cultured at 37°C and 5% CO_2_ for 3–4 d. For adipocyte differentiation, 90% confluent cells were incubated for 1 wk with basal medium (BM-1; Zen-Bio) supplemented with 3% FBS Superior (S0615; Merck), 1 μM dexamethasone (Sigma-Aldrich), 500 μM 3-isobutyl-1-methyxanthine (I5879; Sigma-Aldrich), 1 μM pioglitazone (AZ10080838; AstraZeneca), P/S, and 100 nM insulin (Actrapid; Novo Nordisk [provided by AstraZeneca]). Medium was replaced with BM-1 medium supplemented with 3% FBS Superior, 1 μM dexamethasone, P/S, and 100 nM insulin, and cells were incubated for another 5 d.

HeLa cells were cultured in DMEM supplemented with 10% FBS Superior and 50 μg/ml gentamycin (G1397; Gibco-BRL) at 37°C with 5% CO_2_. For LNP transfection for eGFP expression and endosomal pH measurement studies, 3,000 HeLa cells/well were seeded in 384-well plates using the Multidrop dispenser 1 d before the experiment.

### Chemicals and reagents

FBS Superior, dexamethasone, 3-isobutyl-1-methyxanthine, insulin, and pioglitazone were provided by Astra Zeneca. P/S, transferrin Alexa Fluor 568 (T23365; Invitrogen), EGF Alexa Fluor 488 (E13345; Invitrogen), LDL-pHrodo Red (L34356; Invitrogen), LDL-Alexa Fluor 488 (homemade) were as previously reported (Franke et al., 2019). pH calibration buffers were from Invitrogen (P35379). LNPs were prepared using the cationic ionizable lipids MC3, L608, ACU5, ACU22, MOD5, and L319 (AstraZeneca) and the helper lipids cholesterol (Sigma-Aldrich), 1,2-distearoyl-sn-glycero-3-phosphocholine (DSPC; CordenPharma), 1,2-dimyristoyl-sn-glycero-3-phosphoethanolamine-N [methoxy (polyethylene glycol)-2000] (DMPE-PEG2000; NOF Corporation), and contained CleanCap eGFP mRNA (5-methoxyuridine, cat. no. L-7201) and/or CleanCap Cy5 eGFP mRNA (5-methoxyuridine, batch no. WOTL18871, cat. no. L-7701; TriLink Bio Technologies). Formaldehyde was from Merck and digitonin from Sigma-Aldrich.

### LNP mRNA formulation and characterization

All LNPs were formulated by a bottom–up approach (Zhigaltsev et al., 2012) using a NanoAssemblr microfluidic apparatus (Precision NanoSystems). Lipids were characterized by nuclear magnetic resonance (NMR) for quality control (Supplemental figures, Fig. S32). Before mixing, the lipids were dissolved in ethanol and mixed in the appropriate molar ratios (cationic ionizable lipid:DSPC:cholesterol:DMPE-PEG2000 ratios of 50:10:38.5:1.5), while mRNA was diluted in RNase-free 50 mM citrate buffer, pH 3.0 (Teknova). The aqueous and ethanol solutions were mixed in a 3:1 vol ratio at a mixing rate of 12 ml/min to obtain LNP with an mRNA:lipid wt ratio of 10:1. Finally, the lipids were dialyzed overnight using Slide-A-Lyzer G2 dialysis cassettes with a mol wt cutoff of 10 kD (Thermo Fisher Scientific). The size was determined by DLS measurements using a Zetasizer Nano ZS (Malvern Panalytical). The encapsulation and concentration of mRNA were determined using RiboGreen assay (Table S1). The ζ potential measurements were performed at 7.4 ± 0.1 by diluting LNPs in 1 mM phosphate buffer to a final concentration of ∼1–2 μg/ml mRNA. pH of the samples was measured after dilution and adjusted if needed with a few microliters of 0.1 M NaOH or 0.1 M HCl. The measurements were done using Zetasizer Nano ZS. The reported values were calculated using the Smoluchowski equation and correspond to the average of three measurements and the error to the SD (Table S10).

### LNP-mRNA uptake

For both mature human white-like adipocytes and HeLa cell experiments, LNP at a final mRNA concentration of 1.25 ng/μl was incubated at the indicated time points in the figures. Mature human white-like adipocytes were transfected in the presence of fresh BM-1 medium supplemented with 1% human serum (H4522; Sigma-Aldrich) to mimic the subcutaneous tissue environment. HeLa cells were transfected in the presence of 10% FBS Superior. LNP addition on 384-well plates was done either using a Fluent automated liquid handling robot or manual multichannel pipettes.

### Combined immunofluorescence and smFISH staining

Endosomes and unlabeled mRNA-formulated LNPs were labeled by immunofluorescence and smFISH, respectively. We used an optimized protocol compatible with quantitative retention of the delivered mRNA in the cell cytoplasm (Paramasivam et al., 2021 *Preprint*). Briefly, cells incubated with LNP-mRNA were washed with PBS, fixed with freshly prepared 7.4% formaldehyde for 2 h, washed three times with PBS, and permeabilized with 0.004% digitonin for 2 min (note: digitonin was first purified using the manufacturer’s protocol [product number D5628]. Due to batch variations of this natural product, we recommend to optimize the concentration for each new purchase). After permeabilization, cells were washed and incubated with 3% BSA–PBS blocking solution for 30–45 min. Primary antibodies prepared in 3% BSA–PBS solution were incubated for 2 h and then washed three times with PBS. Secondary antibodies (final concentration of 3% BSA–PBS solution) were incubated for 1 h and washed three times with PBS. After this step, cells were fixed a second time with 3.7% formaldehyde for 10 min to preserve antibody staining and before proceeding with smFISH to fluorescently label mRNAs. The cells were washed three times with PBS, the supernatant was removed manually with an eight-needle aspirator, and 70% ethanol was added for 1 h. After washing the cells with PBS using Tecan Power Washer 384, Wash Buffer A (40 μl/well) was added for 2–5 min. The supernatant was removed, and eGFP–CAL Fluor Red 590 Dye probe (Stellaris) was diluted 1:100 in hybridization buffer (12.5 μl/well) and incubated with cells for 16 h at 37°C. The supernatant was removed, washed two times by incubating 40 μl Wash Buffer A for 30 min at 37°C. Cells were then washed again with Wash Buffer B for 2–5 min. Finally, cells were incubated with DAPI (1 μg/ml) to stain the nuclei and/or cell mask blue (CMB; 0.25 μg/ml) to stain the cytoplasm. All solutions were prepared in nuclease-free PBS, and all blocking and antibody solutions were mixed with 1× final concentration of nuclease inhibitors. Most washing steps and addition of solutions were performed via automated liquid handling robotic systems. All antibodies used in this study and their dilutions are listed in Table S11. We avoided double antibody staining to keep the green fluorescent channel free to subtract autofluorescence from adipocytes by image analysis methods.

### Fluorescence imaging and quantification

All confocal imaging was performed on an automated spinning disc confocal microscope (CV7000; Yokogawa) equipped with an Andor Neo 5.5 sCMOS camera using an UPLSAPO 60×/1.2 NA water immersion objective. Image size was 2,560 × 2,160 pixels. Pixel size was 0.108 μm. In all cases, at least six images were acquired per well, and each condition was in triplicate wells.

Image analysis was performed using custom-designed software (MotionTracking, http://motiontracking.mpi-cbg.de; Collinet et al., 2010). Images were first corrected for illumination, chromatic aberration, and physical shift using multicolor beads. All fluorescent objects in corrected images were then segmented and their number and intensity per image mask area calculated. Cells differentiated into fibroblasts in the same culture were excluded by mask image generated by lipid droplets of adipocytes using a Fiji script. For kinetics and colocalization correlation analysis, the autofluorescence objects were eliminated using green channel as a base. First, the images taken by exciting green laser was used to segment autofluorescence objects. mRNA objects (CAL Fluor Red 590 Dye) colocalizing to autofluorescence objects were then excluded from the analysis. The quality of the quantification was verified by internal nontreated control cells.

Colocalization and statistical analysis were performed using the MotionTracking software. Colocalization of mRNA to endosomal compartments was performed with a threshold of 35% and corrected for random colocalization as described previously (Kalaidzidis et al., 2015b). Image analysis was also done using Fiji (Schindelin et al., 2012) and CellProfiler (Carpenter et al., 2006) software. Briefly, corrected images were preprocessed for segmentation in Fiji, and LNP spots and nuclei were segmented and quantified in CellProfiler. Some data from MotionTracking were loaded in KNIME (Berthold et al., 2008) for visualization with customized R scripts.

### Sample size

No statistical methods were used to predetermine sample size. In general, data presented from confocal microscopy images comprise fluorescent objects (labeled mRNA–formulated LNPs and endosomal compartments) in the range of 1,000 to ≥100,000 objects/condition, depending on LPN formulations, time point, and endosomal compartments observed.

### Differential correlation analysis

For correlation analysis of mRNA (detected by smFISH) distribution in endosomal compartments and eGFP expression, we chose a specific time point in LNP kinetics based on two criteria to exclude false predictions: (1) LNP mRNA signal in the endosomal compartments above the measurement noise level and (2) a time point before mRNA saturation in endosomal compartments. Therefore, given the different lag time from the addition of LNP to the beginning of its intercellular accumulation between experimental repeats, we chose the time point in each experiment at the maximum slope of uptake kinetics. These points varied from 1 to 2 h in five experimental repeats.

The LNP-mRNA traffic through endocytic compartments could be drawn as a DAG, where the nodes of the graph denote the compartments and directed edges (shown as arrows) denote the direction of cargo flow. We expanded such presentation by adding a master node A (Supplemental figures, Fig. S3 A) to denote total uptake of LNP-mRNA by the endocytic system and node Ex to denote release of mRNA to cytoplasm (specifically, expression of eGFP as a proxy for mRNA release). The edges of such an extended graph represent cause–consequence dependencies between amounts of cargo in the endocytic compartments. Indeed, total uptake A is the cause of the amount of LNP-mRNA in compartment C, and the amount of LNP-mRNA in D depends on C, etc. The correlation between the amount of LNP-mRNA in any endocytic compartment and the amount of released mRNA (Ex) decreases proportionally to the stochasticity of the edges connecting these two nodes (regardless of the direction of connections). Indeed, total mRNA escape (node Ex) correlates with all compartments that receive LNP-mRNA from node A (nodes B–F). However, since the escape occurs from compartment D, the correlation between amount of LNP-mRNA in D and eGFP expression is the largest (r_6_). The correlation of the upstream compartment C to eGFP expression Ex is equal to r_C->Ex_ = r_3_ ⋅ r_6_, where r_3_ is the correlation between the amounts of LNP-mRNA in compartment C and D and thus lower than r_6_. Therefore, we chose r_A->Ex_ of total LNP-mRNA uptake (A) to eGFP expression (Ex) as a baseline and subtracted it from the correlations of amount of LNP-mRNA in each endocytic compartment to eGFP expression to get the differential correlation. It is obvious that compartments on the path from uptake A to escape Ex (but not only them) have a differential correlation >0. On the other hand, compartments with differential correlations <0 are located on side branches of the graph (e.g., E, F, and B). Therefore, by ranking compartments according to the differential correlation, such analysis allows reconstructing the DAG of causal dependencies for mRNA delivery (see Fig. 2, A and B).

### Uncertainty estimation for correlation

Let us assume that we have two zero mean experimental vectors $\left\{a_{i}\right\}$ and $\left\{b_{i}\right\}$ with per-element uncertainty estimation $\left\{\delta a_{i}\right\}$ and $\left\{\delta b_{i}\right\}.$ If elements $a_{i}$ and $b_{i}$ are means of experimental measurements, then $\delta a_{i}$ and $\delta b_{i}$ are SEMs.

The correlation is:


$$r=\frac{\sum_{i=1}^{N}a_{i}b_{i}}{\sum_{i=1}^{N}a_{i}^{2}\sum_{i=1}^{N}b_{i}^{2}}.$$

Let us assume that uncertainties of vector elements are independent and drawn from normal distribution. For example, if the elements are mean of experimental measurements, then this assumption is correct as a result of central limit theorem.

Given such an assumption, the variance of correlation can be estimated as:


$$D\left(r\right)=\frac{\sum_{j=1}^{N}{\left(\frac{\partial r}{\partial a_{j}}\delta a_{j}\right)}^{2}+\sum_{j=1}^{N}{\left(\frac{\partial r}{\partial b_{j}}\delta b_{j}\right)}^{2}}{N}.$$

After substitution, the final expression for variance of correlation is:


$$D\left(r\right)=\frac{\sum_{j=1}^{N}{\left(\left(b_{j}-a_{j}c_{a}\right)\delta a_{j}\right)}^{2}+\sum_{j=1}^{N}{\left(\left(a_{j}-b_{j}c_{b}\right)\delta b_{j}\right)}^{2}}{N\sum_{i=1}^{N}a_{i}^{2}\sum_{i=1}^{N}b_{i}^{2}},$$

where

$$c_{a}=\frac{\sum_{i=1}^{N}a_{i}b_{i}}{\sum_{i=1}^{N}a_{i}^{2}},$$

and


$$c_{b}=\frac{\sum_{i=1}^{N}a_{i}b_{i}}{\sum_{i=1}^{N}b_{i}^{2}}.$$

One can see, that if r→1 and $a_{i}\cdot b_{i}>0,$ then D(r)→0 is independent on uncertainty $\delta a_{i}$ and $\delta b_{i}.$

DAG graphical schemes were prepared in Adobe Photoshop 2020 and Inkscape software.

### Statistics

Data presented on Fig. 1, A, B, and G; and Fig. 2, A and C were acquired from three to five independent experiments as described in the figure legends. Each independent experiment consisted of three biological replicates by six technical replicates each. Since each measurement in technical replicates was calculated as a sum of >1,000 endosome signals, the distributions of measurements have to converge to a normal one as stated by central limit theorem. Nevertheless, we tested the normality of pooled technical and biological replicates of each independent experiment (18 replicates per experiment) using Kolmogorov–Smirnov test and found that the P value was between 0.2 and 0.9. All P values presented are calculated by two-sided *t* test. To suppress potential systematic errors, data were aggregated in three steps. First, technical replicates were averaged for each biological replicate. Second, means of biological replicates were averaged within independent experiment. Third, means and SEMs of independent experiments were calculated.

### Ratiometric pH measurement by LDL probes

HeLa cells were seeded 1 d before transfection either in 96- or 384-well plates at a density of either 12,000 or 3,000 cells/well, respectively. pH measurements of LNP-mRNA^+^ endosomes were determined by ratiometric analysis of intensities of pH-sensitive LDL pH Rodo-Red and LDL-Alexa Fluor 488 probes, similar to that previously reported (Tycko and Maxfield, 1982). Briefly, LNP-mRNA (1.25 ng/μl) with or without LDL pH Rodo-Red (20 μg/ml) and LDL-Alexa Fluor 488 (1:100 from homemade stock; see Chemicals and reagents) were cointernalized in cells for 45, 120, and 180 min, imaged live, and fixed at the end of the experiment. The pH calibration measurements were performed on each plate. For this, LDL-pH Rodo-Red/LDL-Alexa Fluor 488 were cointernalized in HeLa cells for 180 min, and cells were fixed (3.7% formaldehyde, 10 min) and incubated in calibration buffers with pH 4.5, 5.5, 6.5, and 7.5 (P35379; Thermo Fisher Scientific) with DAPI/CMB for at least 2 h. The cells were imaged using an automated spinning disc confocal microscope (CV7000; Yokogawa) 60×/1.2 NA (Supplemental figures, Fig. S33 A). From these images, individual endosomes were segmented using MotionTracking software. The integral intensities for both LDL probes in each endosome were calculated for all pH buffer–calibrated conditions (pH 4.5, 5.5, 6.5, and 7.5) in three independent experiments with three replicates per experiment, averaging 74,000 ± 27,000 endosomes for each replicate. As expected, the intensity of Alexa Fluor 488 decreased in acidic buffer, whereas the pH Rodo-Red intensity increased (Supplemental figures, Fig. S33 B). The intensity ratio of LDL-pH Rodo-Red/LDL-Alexa Fluor 488 was then calculated. The ratio increased up to ∼30-fold when pH decreased from 7.5 to 4.5. First, the ratio was calculated for each double-positive endosome (LDL-pH Rodo-Red and LDL-Alexa Fluor 488), then averaged within each replicate. The mean of replicates was calculated for each experiment, and the mean of experiments was then taken as a final value (Supplemental figures, Fig. S33 C). Since in the calibration experiments almost the whole uncertainty was concentrated in the values of ratios, whereas the pH was relatively accurate, the estimation of pH value uncertainty in the calibration curve was calculated by the standard formula of uncertainty propagation:

$$\sigma_{pH}^{2}={\left(\frac{d}{dr}pH\right)}^{2}\cdot \sigma_{r}^{2},$$

where *r* denotes ratio of intensities. The derivative $\frac{d}{dr}pH$ was calculated numerically as a curve slope between two sequential points. From these calculations, we found that the uncertainty of pH $\sigma_{pH}$ in the calibration curve was in the interval from 0.15 to 0.25, with a 95% confidence interval from 0.3 to 0.5 units of pH (Supplemental figures, Fig. S33 D).

Since we aimed to determine the pH distribution in the population of endosomes, we measured a distribution of ratios from the calibration experiments (see example in Supplemental figures, Fig. S34) and converted it into a pH distribution (Supplemental figures, Fig. S34 B). In this example, although the calibration experiments were done at fixed pH (buffer pH 6.5) and the peak of distribution coincides with this pH, the width at half of maximum of the distribution is almost 1 pH unit.

The spread of colors in the images of Fig. S33 A and the consequent broad distribution of ratios of Fig. S33 B (Supplemental figures) are expected because (1) the two endocytic markers (LDL-pHrodo Red and LDL-Alexa Fluor 488) cannot be present in equal amounts in all endosomes; (2) all endosomes cannot be in the middle of the focal plane, and attenuation of light out of focal plane is wavelength dependent; and (3) uncertainty of object segmentation and intensity calculation exists. Next, we applied the method of ratio conversion to the measurements of LDL uptake at different time points (45, 120, and 180 min). Representative images are presented in Fig. S35, the distribution of ratios in Fig. S36 A, and the derived pH distribution in Fig. S36 B (Supplemental figures).

The width of distribution in Figs. S33 B and S36 B (Supplemental figures) may give an impression of low accuracy of the pH measurements. However, the smooth distribution of ratios (Supplemental figures, Figs. S34 A and S36 A), which originated from tens of thousands of endosomes, provides the possibility to apply deconvolution of the distribution of ratios to determine the pH with much higher accuracy. Deconvolution of distribution is a widely used method to analyze data. For this, we revisited the original calibration ratios performed for each individual experiment (Supplemental figures, Fig. S37). Surprisingly, the distributions of ratios significantly varied between experiments and were multimodal for all pH values. Such multimodality could originate from the aforementioned variability of markers and *z*-position of endosomes from one experiment to another.

The expected distribution of ratios is a log-normal distribution. Therefore, we described the measured ratio of distributions as the sum of log-normal distribution of components and used Bayesian analysis to find how many components are required to describe them (Sivia and Carlile, 1992). From this, we found that the most probable number of components was three. Therefore, we globally (simultaneously) fit the calibration of distributions of three independent experiments by the sum of log-normal components, keeping the parameters of components equal for the experiments but allowing different amplitudes:

$$y_{j}\left(x\right)=\sum_{i=1}^{3}\frac{A_{i,j}}{\sqrt{2\pi }\sigma_{i}x}e^{-\frac{1}{2\sigma_{i}^{2}}ln{\left(\frac{x}{\mu_{i}}\right)}^{2}},$$

where $y_{j}\left(x\right)$ is the model distribution of ratios in the repeat *j*; x is ratio of intensities; _$\mu_{i},\sigma_{i}$_ are parameters of component *i*, and $A_{i,j}$ is the contribution of component *i* in the repeat *j*. The resulting parameters $\mu_{i},\sigma_{i}$ as a function of pH are presented in Fig. S39 (Supplemental figures). Given that the fit was performed independently for each pH, the smooth changes of parameters of log-normal components with pH support our three-component model and, importantly, justify the interpolation of ratio distribution for intermediate pH values by linear interpolation of log-normal component parameters (Supplemental figures, Fig. S40).

To find the pH distribution in endocytic compartments, we divided the pH interval from 4.5 to 7.5 on bins by steps of 0.05 units. We introduced amplitudes of all three components for each bin (*A_i_(pH)*, *i* = 1,2,3) as free variables to fit experimental data (Supplemental figures, Fig. S41 A). The fit was performed by library Climb in MotionTracking software. Finally, the pH distribution was calculated as a sum of amplitudes of all three components in each pH bin: $y\left(pH\right)=\sum_{i=1}^{3}A_{i}\left(pH\right).$ The results of such fit for three independent experiments are presented in Fig. S41 B (Supplemental figures).

The robustness of our method is demonstrated by the fact that despite differences in calibration distributions of experimental repeats (Supplemental figures, Fig. S37), the predicted pH distributions of three independent experiments are very much in agreement with each other (Supplemental figures, Fig. S41 B).

An important feature of our measurements is the estimation of uncertainty of the pH values in the peaks of distribution. Since in the deconvolved pH distribution (Supplemental figures, Fig. S42) the uncertainties accumulate in the amplitude but not in the pH values of the distribution, we fitted the pH distribution by sum of Gaussians using a Bayesian method of histogram deconvolution. The fitting procedure was implemented in MotionTracking software by descent gradient with Monte–Carlo stochastic search of starting point. The output of the fitting procedure gives the pH value of each peak (subpopulation of endosomes) and its 95% confidence interval (Table S2 and Table S3). The 95% confidence interval was estimated as ±2 σ of fitted Gaussians.

### LNP uptake and eGFP expression kinetics measurements

For determining the arrested endosome contribution to mRNA escape, we used ACU5 LNP formulated with either unlabeled (eGFP expression) or Cy5 fluorophore–labeled mRNA (uptake). Adipocytes were incubated with ACU5 mRNA formulation for 20 min, 30 min, 45 min, 60 min, 2 h, 4 h, 6 h, 8 h, 12 h, and 24 h and fixed using an optimized protocol (Paramasivam et al., 2021 *Preprint*). The cells were then stained for nuclei (DAPI 1 μg/ml) and cytoplasm (CMB 0.5 μg/ml) and imaged using an automated confocal microscope (CV7000; Yokogawa), and the total eGFP intensity and segmented mRNA intensities were analyzed using MotionTracking software.

### Model for prediction of mRNA escape from arrested endosomes

The experimental data and theoretical model were combined to determine the contribution of the maturation-arrested endosomes to mRNA escape. In our model, we considered two groups of endosomes: active and arrested. The LNPs propagate through active early endosomes and either route toward late endosomes and lysosomes for degradation or long-living arrested endosomes. The amount of LNPs in active and arrested endosomes are denoted as L_e_ and L_s_, respectively (Supplemental figures, Fig. S8 A, Eqs. 1 and 2). The lag in LNP uptake is modeled by the transition function *F*(*t*). The number of LDLRs on the plasma membrane is denoted by *R* (Supplemental figures, Fig. S8 A, Eqs. 1 and 3). Surprisingly, we found that we could not explain the declining LNP uptake (between 8 and 24 h; Fig. 3 C, black dots) without assuming that a massive uptake of LNP leads to the downregulation of LDLR from the plasma membrane (Supplemental figures, Fig. S8 A, Eq. 3). This led us to hypothesize that the arrested endosomes are not sufficiently acidic to release LNPs from LDLRs, thus preventing their recycling to the plasma membrane. This process is modeled by Fig. S8 A, Eq. 3 (Supplemental figures). The fraction of mRNA that leaks from early active and arrested endosomes are described by Fig. S8 A, Eq. 4 (Supplemental figures), where *esc_S_* denotes the ratio of mRNA leakage from arrested to active endosomes. Fig. S8 A, Eq. 5 (Supplemental figures), describes the synthesis and degradation of eGFP as determined previously (Balleza et al., 2018).

### SMLM

Cover glasses of 24-mm diameter were first sonicated in 70% ethanol for 5 min and washed three times using autoclaved water and PBS. The clean cover glasses were distributed on a six-well plate and seeded with HeLa cells at a density of 200,000 cells before day 1 of the experiment. Primary hASCs were seeded at a density of 500,000 cells and differentiated to adipocytes as mentioned in Cell culture. The cells were incubated with LNP-Cy5-mRNA (at a final mRNA concentration of 1.25 ng/μl) for a total of 120 min. EGF-Alexa Fluor 488 (100 ng/ml) and transferrin-Alexa Fluor 568 (10 μg/ml) or homemade LDL-Alexa Fluor 488 (1:100 dilutions, see Chemicals and reagents) were incubated for the last 30 min of LNP uptake. LNP incubation in HeLa cells was supplemented with 10% FBS and primary human adipocytes supplemented with 1% human serum. The cells were then fixed with 7.4% formaldehyde for 2 h and washed three times with PBS.

Multicolor SMLM experiments were performed in standard SMLM imaging buffer (Franke et al., 2017), which sets a homogeneous global pH value of 8.2, on a Nikon Eclipse Ti microscope, which is specified elsewhere in detail (Franke et al., 2019). Before acquisition, cells were irradiated in epifluorescence illumination mode to turn emitters, which were out of focus in the HiLo illumination scheme, into the dark state. In all experiments, the length of the acquisition was set to capture the majority of emitters; i.e., imaging was concluded when only a very minor number of active emitters was detectable. Typical acquisition lengths were 30,000–60,000 frames for 641 and 561 nm excitation and 15,000–30,000 frames for 488 nm excitation. Hereby, mEOS2 was excited at 561 nm and activated with 405 nm. Raw image stacks were analyzed with rapidSTORM 3.2 (Wolter et al., 2012). The FWHM was set as a free fit parameter but in the limits of 275–475 nm for 561 nm and 640 nm excitation and 275–450 nm for 488 nm excitation. This way, we allowed only a narrow axial range (∼500 nm; Franke et al., 2017) to contribute to the final SMLM data set to minimize the likelihood of random axial colocalization. The window radius fit to 1,200 nm, while all other fit parameters were kept from the default settings in rapidSTORM 3.2. Linear lateral drift correction was applied manually by spatiotemporally aligning distinct structures to themselves. This was facilitated by color coding of the temporal coordinate with the built-in tool. The same tool was used to create spatiotemporal images of Cy5-mRNA escape events (Supplemental figures, Fig. S31).

Before imaging samples, a glass surface with TetraSpeck beads (Thermo Fisher Scientific) was imaged with alternating 488 nm, 561 nm, and 641 nm excitation to create a nanometer-precise map to allow the correction of chromatic shift.

Candidates for single LNP were selected in the range of 25–250 nm on the basis of previously reported LNP diameters. FWHMs were determined from the super-resolved images (10-nm pixel size) by Gaussian fitting. Mean diameters for LNPs in cells were determined as (mean ± SD) 74.9 ± 22.6 nm for L608, 71.0 ± 22.0 nm for MC3, 66.7 ± 20.2 nm for ACU5, and 68.9 ± 21.7 nm for MOD5.

In adipocytes, large lipid droplets function as microlenses, making quantitative SMLM-based studies very challenging, since the three-dimensional distortions to the single-molecule emission patterns negate the theoretical advantage of nanometer resolution. Therefore, we imaged only the endosomes underneath the plasma membrane at the bottom of adipocytes by full total internal reflection fluorescence.

### Estimation of cytosolic Cy5-mRNA signal

LNP-mRNA localizations were classified as noncytosolic when satisfying one of the following criteria: (1) object is colocalized with endosomal cargo, (2) object is localized within an arrested endosome, or (3) object is localized on cellular edge (cell border). All other mRNA localizations not fulfilling at least one of these criteria were considered likely to be cytosolic, and the ratio regarding the total number of localizations was given.

According to TriLink Bio Technologies, the Cy5–eGFP mRNA (L-7701; Batch No. WOTL18871) is 75% substituted with 5-methoxy-U and 25% substituted with Cy5-U; in other words, 1:3 ratios (https://www.trilinkbiotech.com/media/productattach/e/g/egfp__orf_catno_l-7201_l-7601_l-7701_.txt; https://www.trilinkbiotech.com/media/productattach//import/coa/L-7701%20WOTL18871.pdf). eGFP mRNA contains 103 uracil, and therefore, 25% substitution results in ∼25 Cy5-U molecules per mRNA. However, it is important to consider that the photophysical properties of the multiple Cy5 molecules per mRNA may vary between the different settings (i.e., mRNA cramped in an LNP, mRNA escaped or fully elongated in the cytosol). The actual fraction might therefore be even lower, since it is to be expected that possible quenching effects between the dyes will occur predominantly in the densely packed environment of the intact LNP, thus reducing the localization count.

### Online supplemental material

Supplemental figures file contains 42 figures. Fig. S1 shows the structure of lipids used for different LNP formulations. Fig. S2 shows representative images for eGFP expression. Fig. S3 shows the DAG scheme for differential correlation analysis of LNP-mRNA delivery. Fig. S4 shows the percentage of EEA1 endosomes colocalized to LNP-mRNA. Fig. S5 shows eGFP expression in an LNP-mRNA–transfected HeLa cell. Fig. S6 shows LDL-Alexa Fluor 488 uptake in HeLa cells. Fig. S7 shows the pH distribution of LNP-containing endosomes in HeLa cells after 45-min uptake. Fig. S8 shows the model for prediction of mRNA escape from arrested endosomes. Fig. S9 shows LNPs on glass surfaces visualized by SMLM. Fig. S10 shows a partial cellular overview of SMLM data in a HeLa cell with regions of interest (ROIs) indicating endosomes presented in Figs. 4 A and 5 A. Fig. S11 shows a partial cellular overview of SMLM data in a HeLa cell with ROIs indicating endosomes presented in Fig. 5 A. Fig. S12 shows a partial cellular overview of SMLM data in a HeLa cell with ROIs indicating endosomes presented in Fig. 5 B. Fig. S13 shows a partial cellular overview of SMLM data in a HeLa cell with ROIs indicating endosomes presented in Fig. 5 C. Fig. S14 shows a partial cellular overview of SMLM data in an adipocyte with ROIs indicating endosomes presented in Fig. 5 D. Fig. S15 shows a partial cellular overview of SMLM data in an adipocyte with ROIs indicating endosomes presented in Figs. 5 D and 6 D. Fig. S16 shows a partial cellular overview of SMLM data in an adipocyte with ROIs indicating additional examples of arrested endosomes for L608 LNP formulation. Fig. S17 shows a partial cellular overview of SMLM data in an adipocyte with ROIs indicating additional examples of arrested endosomes and possible mRNA escape events for the L608 LNP formulation. Fig. S18 shows a partial cellular overview of SMLM data in an adipocyte with ROIs indicating additional examples of arrested endosomes and possible mRNA escape events for the MC3 LNP formulation. Fig. S19 shows a partial cellular overview of SMLM data in an adipocyte with ROIs indicating additional examples of arrested endosomes and possible mRNA escape events for the MC3 LNP formulation. Fig. S20 shows a partial cellular overview of SMLM data in an adipocyte with ROIs indicating additional examples of possible mRNA escape events for the ACU5 LNP formulation. Fig. S21 shows a partial cellular overview of SMLM data in an adipocyte with ROIs indicating additional examples of possible mRNA escape events for the ACU5 LNP formulation. Fig. S22 shows a partial cellular overview of SMLM data in an adipocyte with ROIs indicating additional examples of possible mRNA escape events for the MOD5 LNP formulation. Fig. S23 shows a partial cellular overview of SMLM data in an adipocyte with ROIs indicating additional examples of possible mRNA escape events (L608). Fig. S24 shows a partial cellular overview of SMLM data in a HeLa cell with ROIs indicating the endosome presented in Fig. 6 B. Fig. S25 shows a partial cellular overview of SMLM data in a HeLa cell with ROIs indicating the endosome presented in Figs. 4 C and 6 C. Fig. S26 shows a partial cellular overview of SMLM data in a HeLa cell with ROIs indicating additional examples of possible mRNA escape. Fig. S27 shows a partial cellular overview of SMLM data in HeLa cells with ROIs indicating the endosome presented in Fig. 4 D. Fig. S28 shows exemplary fields of view of cells incubated with LNPs and cargo molecules simultaneously for 30 min. Fig. S29 shows arrested endosomes in primary fibroblasts. Fig. S30 shows the distribution of fitted FWHM of mRNA-Cy5 localizations. Fig. S31 shows the temporally color-coded images of mRNA escape events. Fig. S32 shows NMR characterization data of cationic lipids. Fig. S33 shows representative images of a cointernalized mixture of LDL-pHrodo Red/LDL-Alexa Fluor 488 and ratio measurements. Fig. S34 shows the distribution of ratios and pH for pH 6.5 buffer measurement. Fig. S35 shows representative images of cointernalized LDL-pHrodo Red/LDL-Alexa Fluor 488 kinetics. Fig. S36 shows the distribution of ratios measured in the kinetics experiment. Fig. S37 shows the distribution ratios of integral intensities of pHrodo Red/Alexa Fluor 488 in calibration measurements. Fig. S38 shows the distribution of object ratios of integral intensities of pHrodo Red/Alexa Fluor 488 in live HeLa cells. Fig. S39 shows pH dependency of parameters μ and σ of log-normal components. Fig. S40 shows the predicted distribution of ratios with equal contribution components at pH range 4.5–7.5. Fig. S41 shows the experimental distribution of intensity ratios and fitted distribution of pH. Fig. S42 shows an example of pH estimation by Gaussian fitting for the data presented in Fig. 3 A. Table S1 lists LNP size and encapsulation efficiency calculated by DLS and RiboGreen assays. Table S2 lists the percentages of LNP-Cy5-mRNA–containing endosomes with indicated pH at 2 h. Table S3 lists the percentages of LNP-Cy5-mRNA–containing endosomes with indicated pH at 3 h. Tables S4 lists P values for data presented in Fig. 1 A. Table S5 lists P values for data presented in Fig. 1 B. Table S6 lists P values for the 2-h time point for LNP-mRNA in EEA1 endosomes presented in Fig. 1 G. Table S7 lists P values for the 2-h time point for LNP-mRNA in APPL1 endosomes presented in Fig. 1 G. Table S8 lists P values of the 2-h time point for LNP-mRNA in Rab11 endosomes presented in Fig. 1 G. Table S9 lists the P values for the 2-h time point for LNP-mRNA in LAMP1 endosomes presented in Fig. 1 G. Table S10 lists LNP ζ potential values calculated by Zetasizer. Table S11 details the antibodies and their dilutions used in this study.

## Supplementary Material

Table S1 lists LNP size and encapsulation efficiency calculated by DLS and RiboGreen assays.

Table S2 lists the percentages of LNP-Cy5-mRNA–containing endosomes with indicated pH at 2 h.

Table S3 lists the percentages of LNP-Cy5-mRNA–containing endosomes with indicated pH at 3 h.

Table S4 lists the P values for the data presented in Fig. 1 A.

Table S5 lists the P values for the data presented in Fig. 1 B.

Table S6 lists the P values of 2-h time point for LNP-mRNA in EEA1 endosomes presented in Fig. 1 G.

Table S7 lists the P values of 2-h time point for LNP-mRNA in APPL1 endosomes presented in Fig. 1 G.

Table S8 lists the P values of 2-h time point for LNP-mRNA in Rab11 endosomes presented in Fig. 1 G.

Table S9 lists the P values of 2-h time point for LNP-mRNA in LAMP1 endosomes presented in Fig. 1 G.

Table S10 lists LNP ζ potential values calculated by Zetasizer.

Table S11 details the antibodies and their dilutions used in this study.

Supplemental figure file contains 42 figures.
