# Supplementary material for: Endosomal escape of delivered mRNA from endosomal recycling tubules visualized at the nanoscale
Source: J Cell Biol. 2021 Dec 9;221(2):e202110137. doi: 10.1083/jcb.202110137 (PMC8666849; doi:10.1083/jcb.202110137)
Supplement: Table S1 — lists LNP size and encapsulation efficiency calculated by DLS and RiboGreen assays. [file JCB_202110137_TableS1.docx]

Supplementary Table 1: LNPs size and encapsulation efficiency calculated by DLS and Ribo green

assays

| **CIL** | **<Z> (nm)** | **PDI** | **<N> (nm)** | **Encapsulation (%)** |
| --- | --- | --- | --- | --- |
| **L608** | 82 ± 3 | 0.03 ± 0.01 | 68 ± 4 | 97 ± 1 |
| **MC3** | 81 ± 4 | 0.03 ± 0.01 | 67 ± 4 | 98 ± 1 |
| **ACU5** | 82 ± 4 | 0.03 ± 0.01 | 67 ± 5 | 97 ± 1 |
| **ACU22** | 72 ± 6 | 0.08 ± 0.02 | 54 ± 7 | 96 ± 2 |
| **MOD5** | 75 ± 4 | 0.03 ± 0.01 | 62 ± 7 | 96 ± 1 |
| **L319** | 90 ± 2 | 0.06 ± 0.02 | 73 ± 6 | 88 ± 5 |

<Z>: Intensity-averaged size, PDI: polydispersity index, <N>: Number-averaged size

**Supplementary Table 1: LNPs size and encapsulation efficiency calculated by DLS and Ribo green assays.**
