# Supplementary material for: Endosomal escape of delivered mRNA from endosomal recycling tubules visualized at the nanoscale
Source: J Cell Biol. 2021 Dec 9;221(2):e202110137. doi: 10.1083/jcb.202110137 (PMC8666849; doi:10.1083/jcb.202110137)
Supplement: Table S2 — lists the percentages of LNP-Cy5-mRNA–containing endosomes with indicated pH at 2 h. [file JCB_202110137_TableS2.docx]

Supplementary Table 2: Percentage of LNP-Cy5 mRNA containing endosomes with indicated pH-2h

| **Endosomes with** | **Average pH** | **95% confidence interval** | **% of endosomes** |
| --- | --- | --- | --- |
| **LDL probes alone** | 5.56 | [5.4, 5.7] | 78 |
|  | 6.5 | [6.4, 6.6] | 5 |
|  | 7 | [6.7, 7.3] | 12 |
|  | Other pH values close to background |  | 5 |
| **L608** | 5.12 | [5.06, 5.18] | 16 |
|  | 6.06 | [6.0, 6.16] | 46 |
|  | 6.49 | [6.37, 6.63] | 20 |
|  | Other pH values close to background |  | 18 |
| **MC3** | 4.99 | [4.81, 5.17] | 6 |
|  | 6.05 | [5.96, 6.14] | 58 |
|  | 6.5 | [6.43, 6.57] | 13 |
|  | Other pH values close to background |  | 23 |
| **ACU5** | 5.95 | [5.88, 6.02] | 73 |
|  | 6.52 | [6.48, 6.56] | 8 |
|  | Other pH values close to background |  | 19 |
| **ACU22** | 5.88 | [5.79, 5.98] | 77 |
|  | 6.5 | [6.46, 6.54] | 7 |
|  | Other pH values close to background |  | 16 |
| **MOD5** | 5.78 | [5.67, 5.89] | 83 |
|  | 6.56 | [6.52, 6.60] | 2 |
|  | Other pH values close to background |  | 15 |
| **L319** | 5.6 | [5.48, 5.71] | 78 |
|  | 6.02 | [5.67, 6.37] | 3 |
|  | 6.87 | [6.53, 7.21] | 2 |
|  | Other pH values close to background |  | 17 |

**Supplementary Table 2: Percentage of LNP-Cy5-mRNA containing endosomes with indicated pH.** HeLa cells were incubated with LNP-Cy5-mRNA and LDL pH probes for 2h and pH of the LNP-mRNA containing endosomes were calculated **(see Methods)**. The percentage of endosomes with an average pH of characteristic late endosomes in control (LDL alone) is shown in green shading. The control cells (LDL alone) have 5% of LDL-positive objects with pH of 6.5 and 74% a pH of 5.5, characteristic of early and late endosomes, respectively The rest (21%) of endosomes displayed pH values ranging between neutral (endocytic vesicles, e.g. Clathrin-coated vesicles) and intermediate acidic values. The percentage of arrested endosomes with a pH values between late and early endosomes are shown in red color shade. The confidence interval was calculated as ±2σ of Gaussians fitted to experimental distributions (see Fig.S42 and Methods).
