# Supplementary material for: Endosomal escape of delivered mRNA from endosomal recycling tubules visualized at the nanoscale
Source: J Cell Biol. 2021 Dec 9;221(2):e202110137. doi: 10.1083/jcb.202110137 (PMC8666849; doi:10.1083/jcb.202110137)
Supplement: Table S3 — lists the percentages of LNP-Cy5-mRNA–containing endosomes with indicated pH at 3 h. [file JCB_202110137_TableS3.docx]

Supplementary Table 3: Percentage of LNP-Cy5 mRNA containing endosomes with indicated pH-3h

| **Endosomes with** | **Average pH** | **95% confidence interval** | **% of endosomes** |
| --- | --- | --- | --- |
| **LDL alone** | 5.52 | [5.36, 5.67] | 74 |
|  | 6.48 | [6.42, 6.54] | 5 |
|  | - |  | - |
|  | Other pH values close to background |  | 5 |
| **L608** | 5.8 | [5.74, 5.86] | 13 |
|  | 6.16 | [5.85, 6.47] | 49 |
|  | 6.52 | [6.39, 6.65] | 15 |
|  | 6.92 | [6.33, 7.50] | 19 |
|  | Other pH values close to background |  | 18 |
| **MC3** | 6.1 | [5.63, 6.57] | 17 |
|  | 6.3 | [6.10, 6.50] | 48 |
|  | 6.54 | [6.44, 6.64] | 10 |
|  | 6.9 | [6.64, 7.16] | 12 |
|  | Other pH values close to background |  | 13 |
| **ACU5** | 6.1 | [6.03, 6.16] | 89 |
|  | 6.5 | [6.43, 6.57] | 5 |
|  | Other pH values close to background |  | 6 |
| **ACU22** | 5.95 | [5.86, 6.04] | 76 |
|  | 6.5 | [6.42, 6.58] | 13 |
|  | Other pH values close to background |  | 11 |
| **MOD5** | 5.9 | [5.82, 5.99] | 92 |
|  | 6.49 | [6.40, 6.58] | 5 |
|  | Other pH values close to background |  | 3 |
| **L319** | 5.5 | [5.40, 5.60] | 91 |
|  | 6.53 | [6.40, 6.66] | 2 |
|  | Other pH values close to background |  | 7 |

**Supplementary Table 3: Percentage of LNP-Cy5-mRNA containing endosomes with indicated pH**. LNP-Cy5-mRNA and LDL pH probes where incubated for 3h in HeLa cells and pH of LNP-Cy5-mRNA containing endosomes were calculated **(see Methods)**. The percentage of endosomes with an average pH of characteristic late endosomes in control (LDL alone) is shown in green shading. The percentage of arrested endosomes with a pH values between late and early endosomes are shown in red color shade. The confidence interval was calculated as ±2σ of Gaussians fitted to experimental distributions (see Fig.S42 and Methods).
