# Supplementary material for: Endosomal escape of delivered mRNA from endosomal recycling tubules visualized at the nanoscale
Source: J Cell Biol. 2021 Dec 9;221(2):e202110137. doi: 10.1083/jcb.202110137 (PMC8666849; doi:10.1083/jcb.202110137)
Supplement: Table S4 — lists the P values for the data presented in Fig. 1 A. [file JCB_202110137_TableS4.docx]

Supplementary Table 4: p-values for figures presented in Figure 1 A-B and Figure 1G

| **P vlaue relative to** | **L608** | **MC3** | **ACU5** | **ACU22** | **MOD5** | **L319** |
| --- | --- | --- | --- | --- | --- | --- |
| **L608** | - | 0.188 | 0.119 | <0.001 | <0.001 | <0.001 |
| **MC3** | 0.188 | - | 0.539 | <0.001 | <0.001 | <0.001 |
| **ACU5** | 0.119 | 0.539 | - | 0.123 | 0.050 | 0.004 |
| **ACU22** | <0.001 | <0.001 | 0.123 | - | 0.404 | 0.017 |
| **MOD5** | <0.001 | <0.001 | 0.050 | 0.404 | - | 0.220 |
| **L319** | <0.001 | <0.001 | 0.004 | 0.017 | 0.220 | - |

**Supplementary Table 4:** p values for the data presented in Figure 1A. p-values are calculated by two sided student-t test. The normality of data tested by Kolmogorov-Smirnov test (see Methods section Statistics).
