# Supplementary material for: Endosomal escape of delivered mRNA from endosomal recycling tubules visualized at the nanoscale
Source: J Cell Biol. 2021 Dec 9;221(2):e202110137. doi: 10.1083/jcb.202110137 (PMC8666849; doi:10.1083/jcb.202110137)
Supplement: Table S7 — lists the P values of 2-h time point for LNP-mRNA in APPL1 endosomes presented in Fig. 1 G. [file JCB_202110137_TableS7.docx]

Supplementary Table 7: p-values for figures presented in Figure 1 A-B and Figure 1G

| **P vlaue relative to** | **L608** | **MC3** | **ACU5** | **ACU22** | **MOD5** | **L319** |
| --- | --- | --- | --- | --- | --- | --- |
| **L608** | - | 0.091 | 0.474 | 0.038 | 0.055 | 0.010 |
| **MC3** | 0.091 | - | 0.410 | <0.001 | <0.001 | <0.001 |
| **ACU5** | 0.474 | 0.410 | - | 0.001 | 0.001 | <0.001 |
| **ACU22** | 0.038 | <0.001 | 0.001 | - | 0.626 | 0.174 |
| **MOD5** | 0.055 | <0.001 | 0.001 | 0.626 | - | 0.013 |
| **L319** | 0.010 | <0.001 | <0.001 | 0.174 | 0.013 | - |

**Supplementary Table 7:** p values of 2h time point for LNP-mRNA in APPL1 endosomes presented in Figure 1G. p-values are calculated by two sided student-t test. The normality of data tested by Kolmogorov-Smirnov test (see Methods section Statistics).
