# Supplementary material for: Endosomal escape of delivered mRNA from endosomal recycling tubules visualized at the nanoscale
Source: J Cell Biol. 2021 Dec 9;221(2):e202110137. doi: 10.1083/jcb.202110137 (PMC8666849; doi:10.1083/jcb.202110137)
Supplement: Table S10 — lists LNP ζ potential values calculated by Zetasizer. [file JCB_202110137_TableS10.docx]

Supplementary Table 10: LNP zeta potential values calculated by zetasizer.

| **CIL** | **Zeta potential (mV)** | **Std. Dev** |
| --- | --- | --- |
| **L608** | -4 | 1 |
| **MC3** | -11.5 | 1.8 |
| **ACU5** | -11.5 | 2.1 |
| **ACU22** | -8.5 | 1.4 |
| **MOD5** | -8.2 | 1.1 |
| **L319** | -10.9 | 1.5 |

**Supplementary Table 10:** LNP zeta potential values calculated by zetasizer.
