# Supplementary material for: Endosomal escape of delivered mRNA from endosomal recycling tubules visualized at the nanoscale
Source: J Cell Biol. 2021 Dec 9;221(2):e202110137. doi: 10.1083/jcb.202110137 (PMC8666849; doi:10.1083/jcb.202110137)
Supplement: Table S11 — details the antibodies and their dilutions used in this study. [file JCB_202110137_TableS11.docx]

Supplementary Table 11: Details of antibodies and their dilutions used in this study

| **Antibody name** | **Company** | **Product number** | **Antibody dilution used** | **RRID number** |
| --- | --- | --- | --- | --- |
| **Mouse monoclonal antibody against human CD107a (LAMP1)** | BD Bioscience | 555798 | 1 to 200 | AB_396132 |
| **Rabbit polyclonal antibody against APPL1** | Produced at Eurogentec, Belgium (Miaczynska et al., 2004). Purified at MPI-CBG | α APPL1 2624-3 | 1 to 250 | RRID not available |
| **Rabbit polyclonal antibody against EEA1** | Produced at EMBL (Simonsen et al., 1998). Purified at MPI-CBG | α EEA1 f.1 Ø7JF | 1 to 1000 | RRID not available |
| **Rabbit polyclonal antibody against Rab11A** | Invitrogen (Thermo Fisher) | 71-5300 | 1 to 250 | AB_2533987 |
| **Mouse polyclonal antibody against ANKFY1** | Sigma Aldrich | SAB1401696-50UG | 1 to 100 | AB_10607865 |
| **Mouse monoclonal antibody against LC3** | MBL | M152-3 | 1 to 500 | AB_1279144 |
| **Mouse monoclonal antibody against LBPA** | Echelon Biosciences/ MoBiTec | Z-SLBPA | 1 to 100 | AB_11127192 |
| **Rabbit polyclonal antibody against CAV1** | Cell Signaling | #3238 | 1 to 100 | AB_2072166 |
| **Donkey polyclonal anti rabbit Alexa Fluor 647** | Invitrogen (Thermo Fisher) | # A31573 | 1 to 1000 | AB_2536183 |
| **Donkey polyclonal anti mouse Alexa Fluor 647** | Invitrogen (Thermo Fisher) | # A31571 | 1 to 1000 | AB_162542 |

**Supplementary Table 11: Details of antibodies and their dilutions used in this study.**
