# Supplementary material for: Endosomal escape of delivered mRNA from endosomal recycling tubules visualized at the nanoscale
Source: J Cell Biol. 2021 Dec 9;221(2):e202110137. doi: 10.1083/jcb.202110137 (PMC8666849; doi:10.1083/jcb.202110137)
Supplement: Supplemental figure — file contains 42 figures. [file JCB_202110137_DataS1.pdf]

Summary of supplementary figures

Figure S1: Structure of lipids used for different LNP formulation

Figure S2: Representative images for eGFP expression

Figure S3: DAG scheme for differential correlation analysis of LNP-mRNA delivery

Figure S4: Percentage of EEA1 endosomes co-localized to LNP-mRNA

Figure S5. eGFP expression in LNP-mRNA transfected HeLa cells

Figure S6. LDL-488 uptake in HeLa cells

Figure S7. pH distribution LNP-containing endosomes in HeLa cells after 45min uptake

Figure S8. Model for prediction of mRNA escape from arrested endosomes

Figure S9: LNPs on glass surfaces visualized by SMLM

Figure S10 -13: Partial cellular overview of SMLM data in a HeLa cell

Figure S14 - S22: Partial cellular overview of SMLM data in an adipocyte with ROIs indicating additional examples of endosomes and arrested endosomes for the L608 MC3, ACU5 and MOD5 LNP formulations

Figure S23: Partial cellular overview of SMLM data in an adipocyte with ROIs indicating additional examples of possible mRNA escape events –L608

Figure S24 -25: Partial cellular overview of SMLM data in a HeLa cell with ROIs indicating the endosome presented in Figure 6B, Figure 4C and Figure 6C

Figure S26: Partial cellular overview of SMLM data in a HeLa cell with ROIs indicating additional examples of possible mRNA escape

Figure S27: Partial cellular overview of SMLM data in HeLa cells with ROIs indicating the endosome presented in Figure 4D

Figure S28: Exemplary field of views of cells incubated with LNPs and cargo molecules simultaneously for 30 minutes

Figure S29: Arrested endosomes in primary fibroblasts

Figure S30: Distribution of fitted FWHM of mRNA-Cy5 localizations

Figure S31: The temporally color-coded images of mRNA escape events

Figure S32: NMR characterization data of cationic lipids (Page 33 – 35)

Figure S33: Representative images of co-internalized mixture of LDL-pHRodo-Red/LDL-Alexa-488 and ratio measurements.
Figure S34. Distribution of ratios and pH for pH 6.5 buffer measurement.

Figure S35. Representative images of co-internalized LDL-pHrodo-Red/LDL-Alexa-488 kinetics.

Figure S36. Distribution of ratios measured in the kinetics experiment.

Figure S37: Distribution ratios of integral intensities of pHRodo-Red/Alexa-488 in calibration measurements

Figure S38: Distribution of objects ratios of integral intensities of pHRodo-Red/Alexa-488 in live HeLa cells

Figure S39: pH dependency of parameters μ and σ of log-normal components

Figure S40: Predicted distribution of ratios with equal contributions components at pH in the range from 4.5 to 7.5

Figure S41: Experimental distribution of intensities ratios and Fitted distribution of pH.

Figure S42: Example figure of pH estimation by Gaussian fitting for the data presented in Figure 3 A.

L608

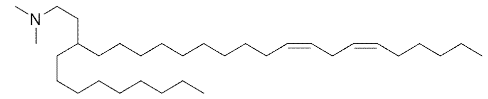

WO 2017/201350 A1, L608

MC3

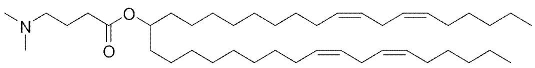

Jayaraman, Ansell et al. 2012

ACU5

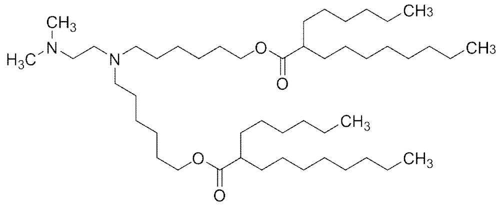

WO 2015/199952 A1, compound 5

ACU22

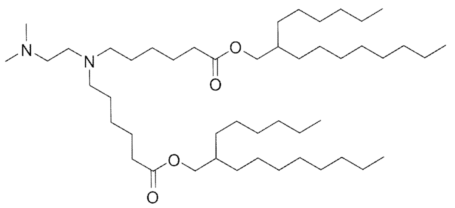

WO 2015/199952 A1, compound 22

MOD5

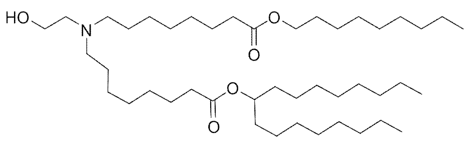

Sabnis, Kumarasinghe et al. 2018

L319

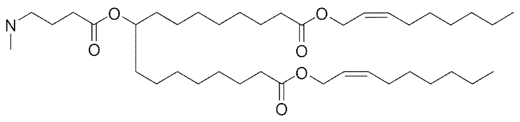

Maier, Jayaraman et al. 2013

Figure S1: Structure of cationic lipids used in different LNP-mRNA formulations.(Ansell, 2015; Frederick, 2017; Jayaraman et al., 2012; Maier et al., 2013; Sabnis et al., 2018)

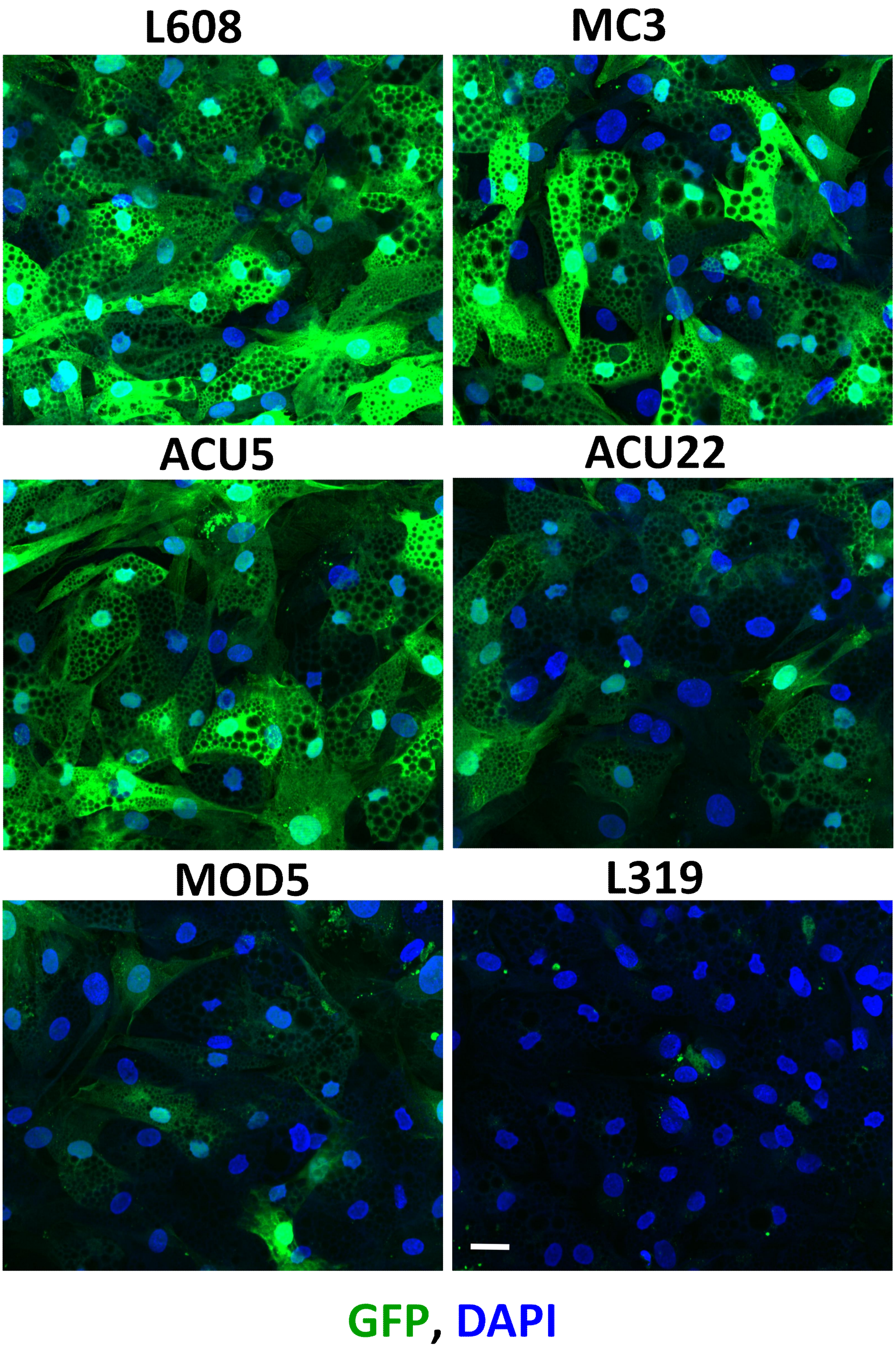

Figure S2: Representative images of human primary adipocytes expressing eGFP after 24h of LNP-mRNA uptake. The cells were fixed, stained for DAPI and imaged by fluorescence microscope. Bar indicates 20µm for all images.

DAG analysis schematic

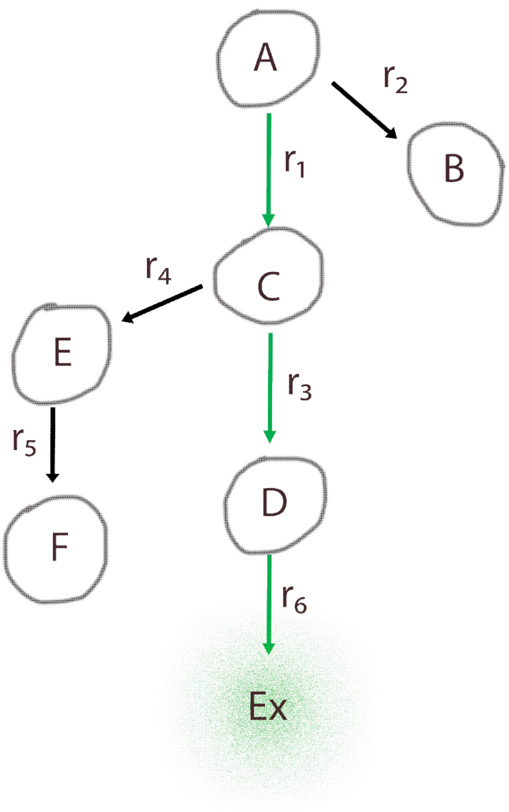

Figure S3: DAG scheme for differential correlation analysis of LNP-mRNA delivery. The first (root) node A represents the total uptake of LNP-mRNA and the last node Ex represents eGFP expression as proxy for mRNA escape. All other nodes of the graph represent the amount of LNP-mRNA in different endocytic compartments received from node A. Compartments on the path from mRNA uptake to endosomal mRNA escape are represented by green edges. Compartments that contribute minimally to mRNA escape are on the side branches (black edges, E, F and B). (**see Methods**).

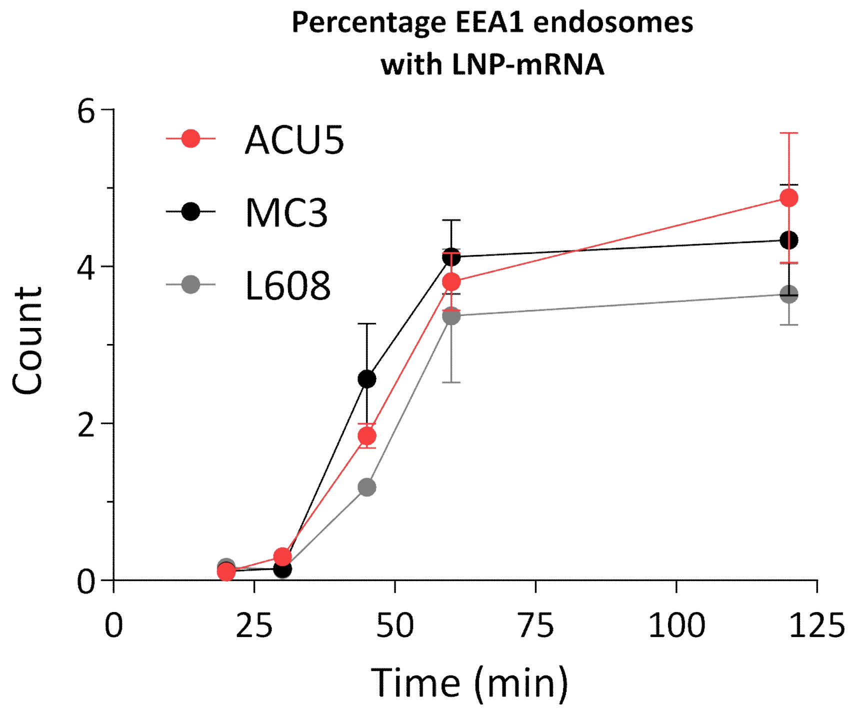

Figure S4. Percentage of EEA1 endosomes co-localized to LNP-mRNA. The graph illustrates that only low percentages of EEA1 endosomes contain LNP-mRNA after 2h of uptake.

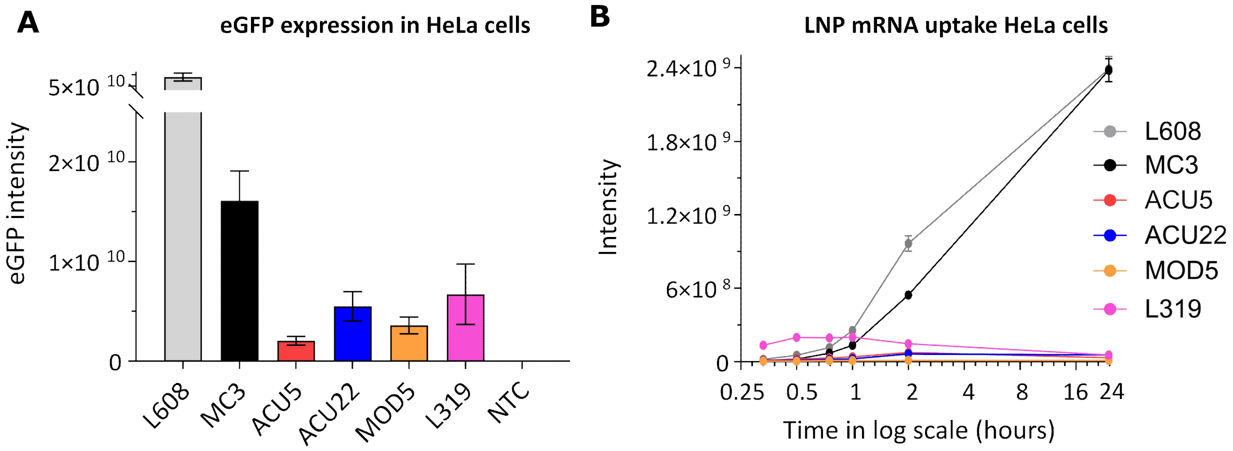

Figure S5. eGFP expression in LNP-mRNA transfected HeLa cells. **(A)** HeLa cells were incubated with LNP-mRNA (1.25ng/μL) for 24h, fixed and imaged. eGFP expression was quantified by MotionTracking software. n=4 independent experiments. **(B)** Cells incubated with LNP-mRNA as described above were fixed at the given time point, processed for smFISH to fluorescently label mRNA and imaged by fluorescence microscopy. The total intensity of LNP-mRNA containing endosomes per image mask was calculated. n = 2 independent experiments.

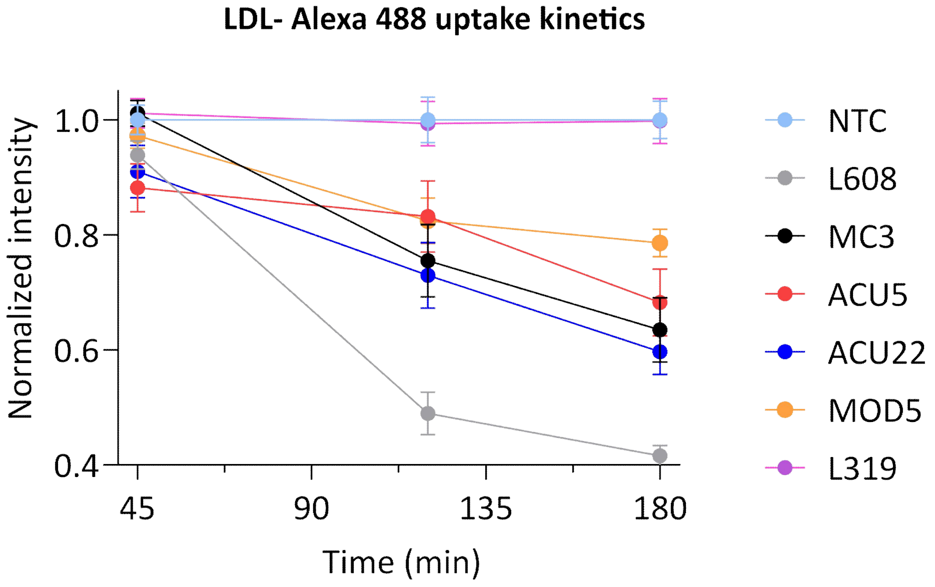

Figure S6. LDL-488 uptake in HeLa cells. LDL-Alexa Fluor 488 total uptake kinetics in HeLa cells that were co-incubated with LNP-Cy5-mRNA (1.25ng/µL). The total LDL uptake was quantified by integrated intensity of LDL positive objects and normalized to non-LNP-treated control NTC (i.e LDL uptake without LNP-Cy5-mRNA). The graph shows that when cells treated with LNP-Cy5-mRNA, the uptake of LDL is mildly affected at 45min, at which time point the pH of endosomes is not severely impaired (see Figure S7). However, the LDL uptake was significantly reduced over time especially when cells were treated with LNP-Cy5-mRNA that blocked endosomal acidification at the given time points (Supplementary Table 2 & 3). L319 LNP-Cy5-mRNA does not block endosomal acidification and show LDL uptake similar to that of non-LNP-treated control (NTC).

pH distribution of LNP-Cy5-mRNA containing endosome populations - 45min

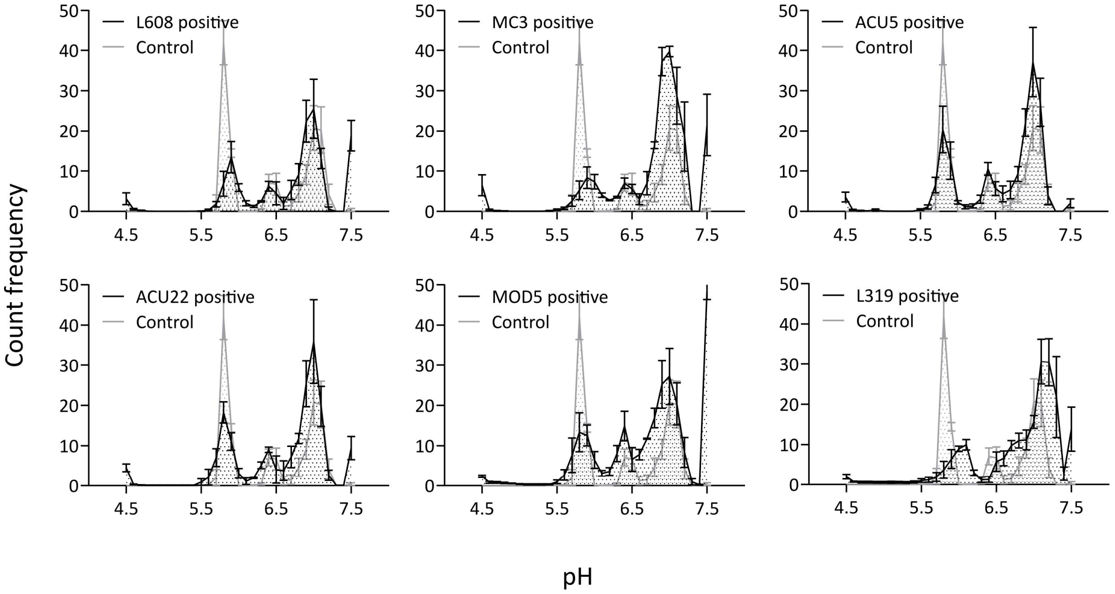

Figure S7: pH distribution LNP-Cy5-mRNA containing endosomes in HeLa cells. LDL-probes co-incubated with LNP-Cy5-mRNA (1.25ng/µL) for 45min and imaged live. The graph shows that the pH of LNP-Cy5 mRNA containing endosomes are only mildly affected in contrast to 120min and 180min time points presented in Figure 3 A, Supplementary Table 2, and 3.

A

$$F(t) = 1 - e^{-\left(\frac{t}{t_0}\right)^2}$$

$$\begin{cases} \dfrac{dL_e}{dt} = F(t)\cdot k_{in}\cdot R - k_{\deg_0}\cdot L_e - k_s\cdot L_e & (1) \\ \dfrac{dL_s}{dt} = k_s\cdot L_e - k_{\deg}\cdot L_s & (2) \\ \dfrac{dR}{dt} = k_{ldl\_syn} - k_{ldl\_\deg}\cdot R - F(t)\cdot k_{in}\cdot R & (3) \\ \dfrac{dmGFP}{dt} = \dfrac{1}{1+esc_s}L_e + \dfrac{esc_s}{1+esc_s}\cdot L_s - k_{m\_\deg}\cdot mGFP & (4) \\ \dfrac{dGFP}{dt} = k_{syn}\cdot mGFP - k_{g\_\deg}\cdot GFP & (5) \end{cases}$$

B

Fit Residue: 7.48221 χ^2 5.58948
LogLikelihood: -56.5035 DoF: 17
Numb. Equations: 7 ☐ Inverse Numb. Params: 10 / 11

In...	Param. Name	Value	Confidence Interval	Fit
1	esc_s	1.00018e-005	[8.36762e-006, 1.19552e-005]	✓
2	g_deg	0.0384615		✗
3	k_deg	1.00012e-005	[8.81751e-006, 1.13439e-005]	✓
4	k_deg0	13.0016	[3.40628, 49.6261]	✓
5	k_in	0.343861	[0.179387, 0.659134]	✓
6	k_ldl_deg	0.000100004	[9.99967e-005, 0.000100012]	✓
7	k_s	0.077326	[0.0551474, 0.108424]	✓
8	k_syn	0.108306	[0.0782092, 0.149984]	✓
9	ldlr_syn	0.00978421	[0.00260095, 0.0368062]	✓
10	m_deg	0.504481	[0.300999, 0.84552]	✓
11	t0	10.5327	[7.71161, 14.3859]	✓

Figure S8. (A-B) Model for prediction of mRNA escape from arrested endosomes**.** The parameters fitted with 95% confidence intervals on experimental data presented in Figure 3 B & C (black dots). The theoretical fit brings the fraction mRNA escape from arrested endosomes to zero (i.e esc_s = 10^{-5}, confidence interval $8.3\cdot10^{-6}\div1.2\cdot10^{-5}$). Uncertainty of parameters distribution was approximated by Gaussian distribution with inverse Hessian of log-likelihood as a covariance matrix. From this approximation the 95% confidence interval was calculated. (**see Methods).**

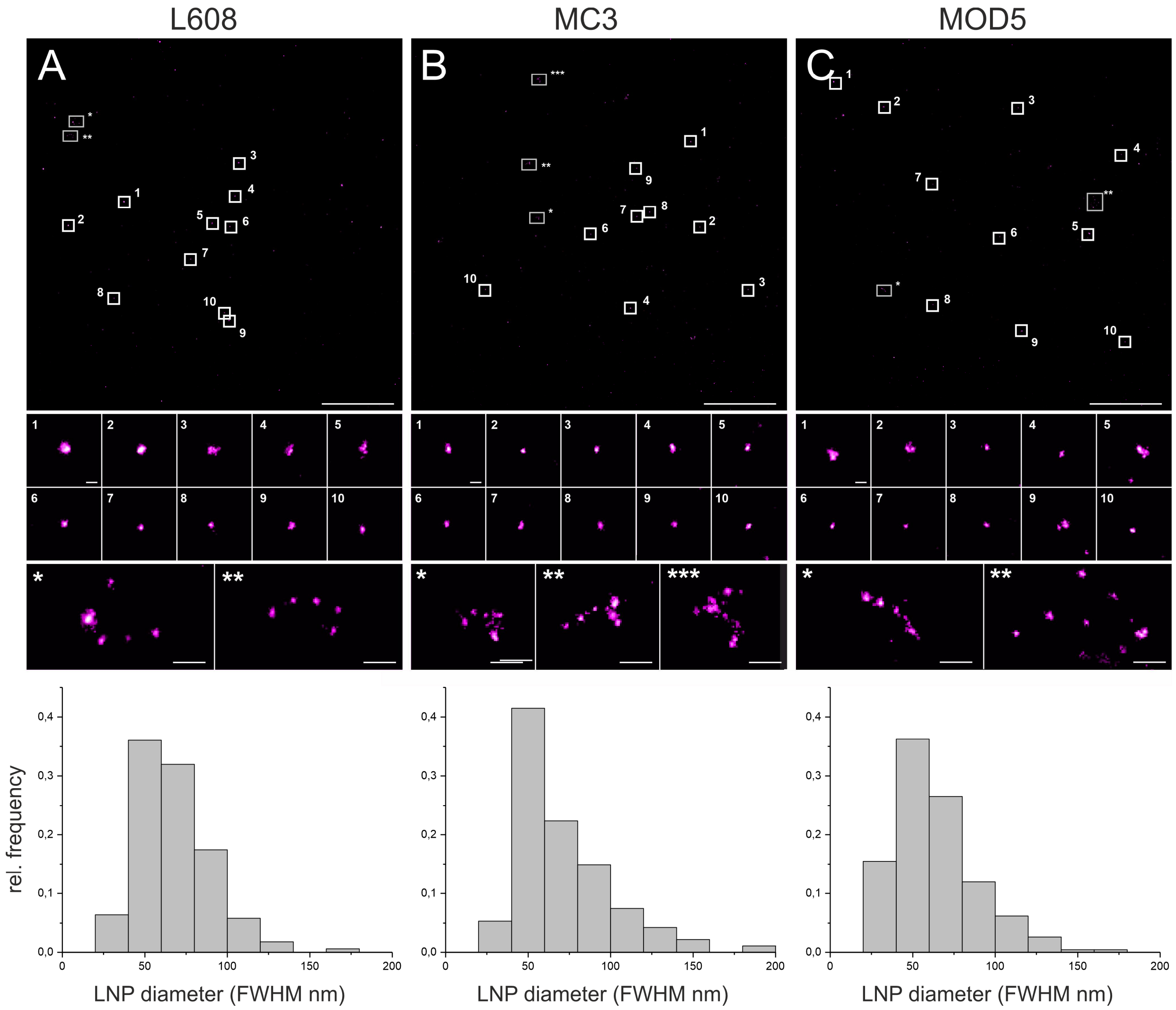

Figure S9. Exemplary field of views of LNP-Cy5-mRNA (magenta) deposited on glass surfaces visualized by SMLM. All imaged LNP-mRNA formulations were detected as concentrated small spots with consistent sizes over the entire field of view. Diameters (FWHM) were determined to 67.4 ± 22.2 nm, 72.9 ± 32.3 nm and 63.9 ± 25.0 nm for LNPs L608, MC3 and MOD5 respectively (mean ± sd). Scale bars 5 µm.

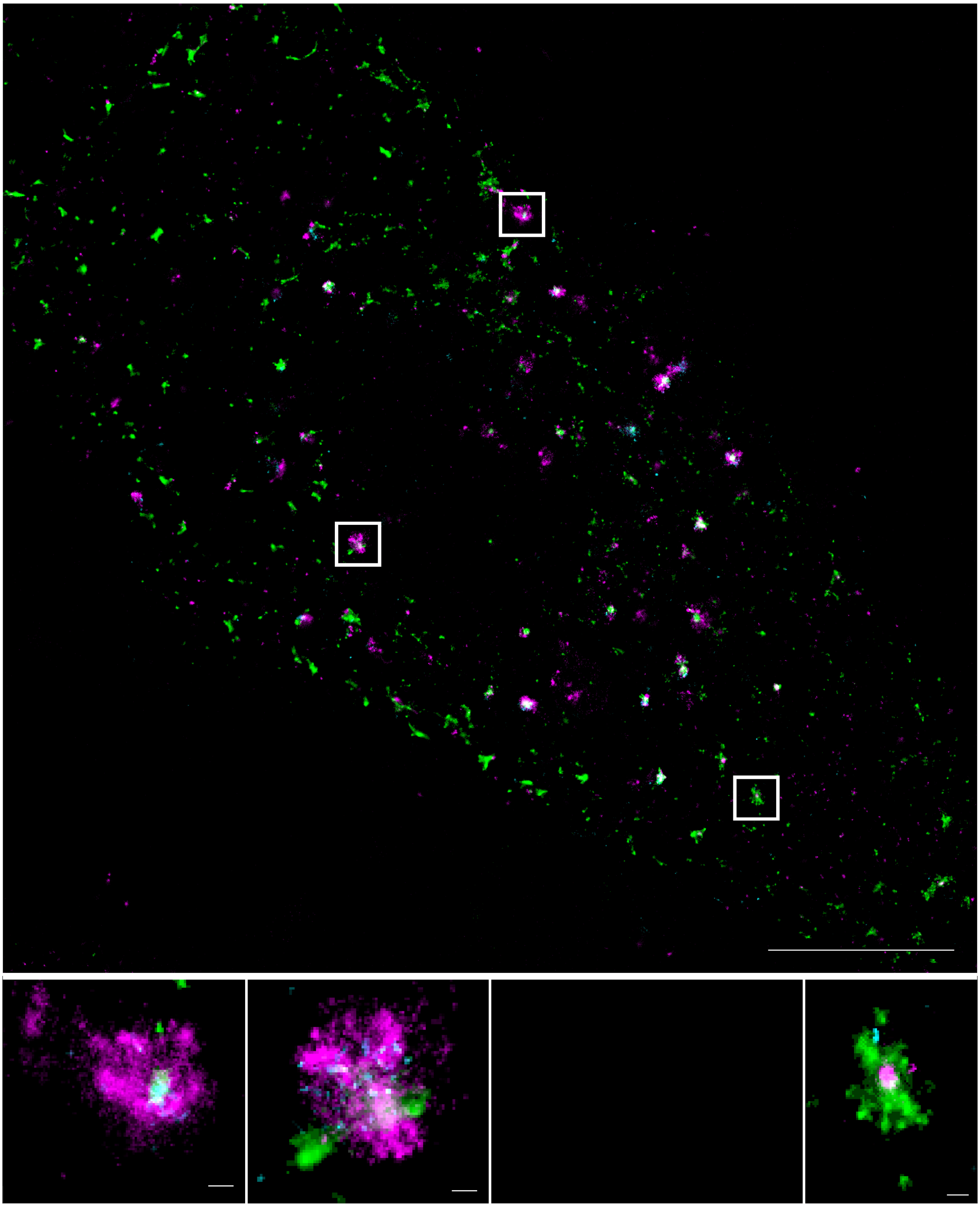

Figure S10. Partial cellular overview of SMLM data in a HeLa cell with ROIs indicating endosomes presented in Figure 4 A and Figure 5 A. LNP-Cy5-mRNA (magenta), Transferrin (green), EGF (cyan). Scale bars 5 µm (top), 100nm (bottom).

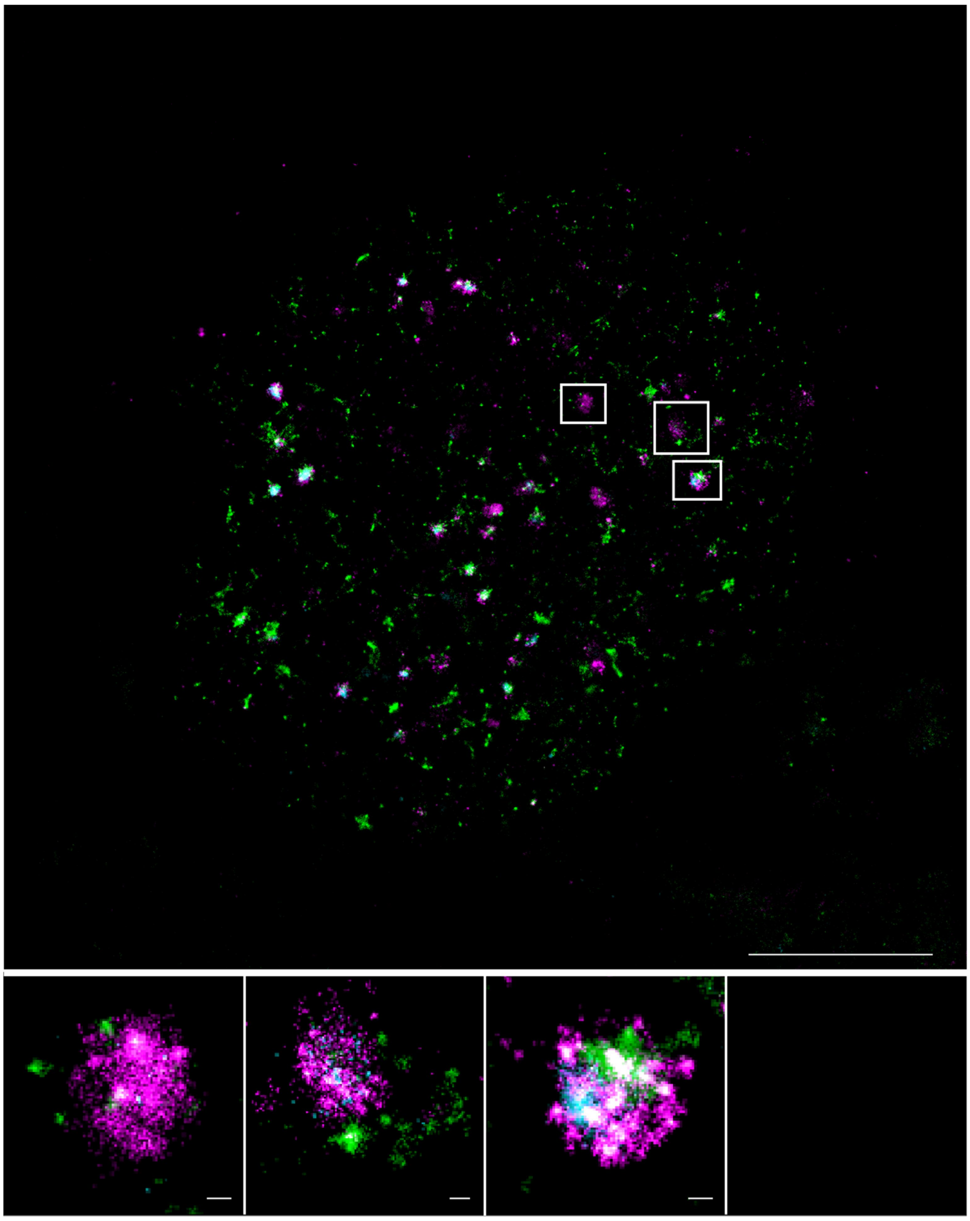

Figure S11. Partial cellular overview of SMLM data in a HeLa cell with ROIs indicating endosomes presented in Figure 5 A. LNP-Cy5-mRNA (magenta), Transferrin (green), EGF (cyan). Scale bars 5 µm (top), 100nm (bottom).

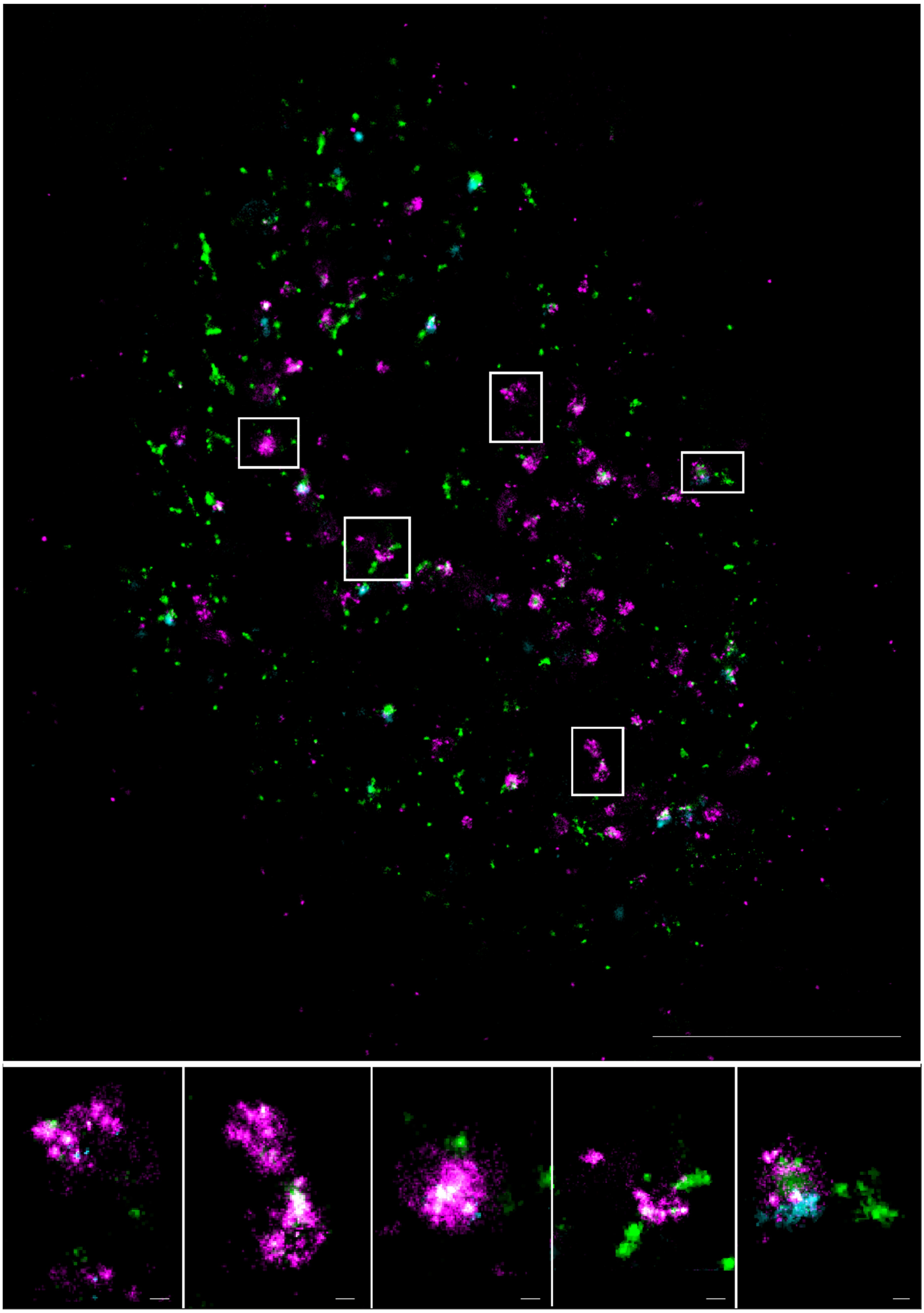

Figure S12. Partial cellular overview of SMLM data in a HeLa cell with ROIs indicating endosomes presented in Figure 5 B. LNP-Cy5-mRNA (magenta), Transferrin (green), EGF (cyan). Scale bars 5 µm (top), 100nm (bottom).

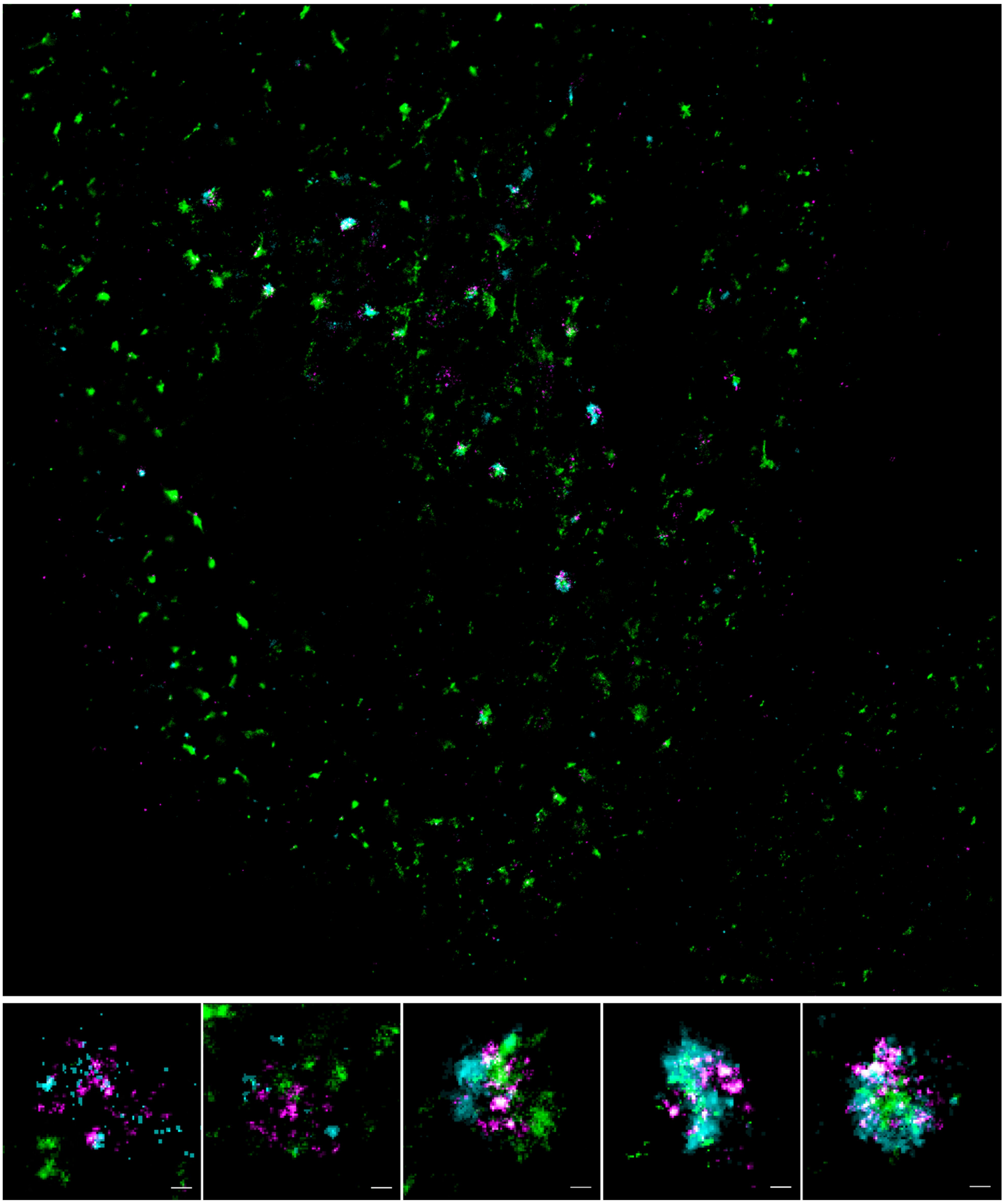

Figure S13. Partial cellular overview of SMLM data in a HeLa cell with ROIs indicating endosomes presented in Figure 5 C. LNP-Cy5-mRNA (magenta), Transferrin (green), EGF (cyan). Scale bars 5µm (top), 100nm (bottom).

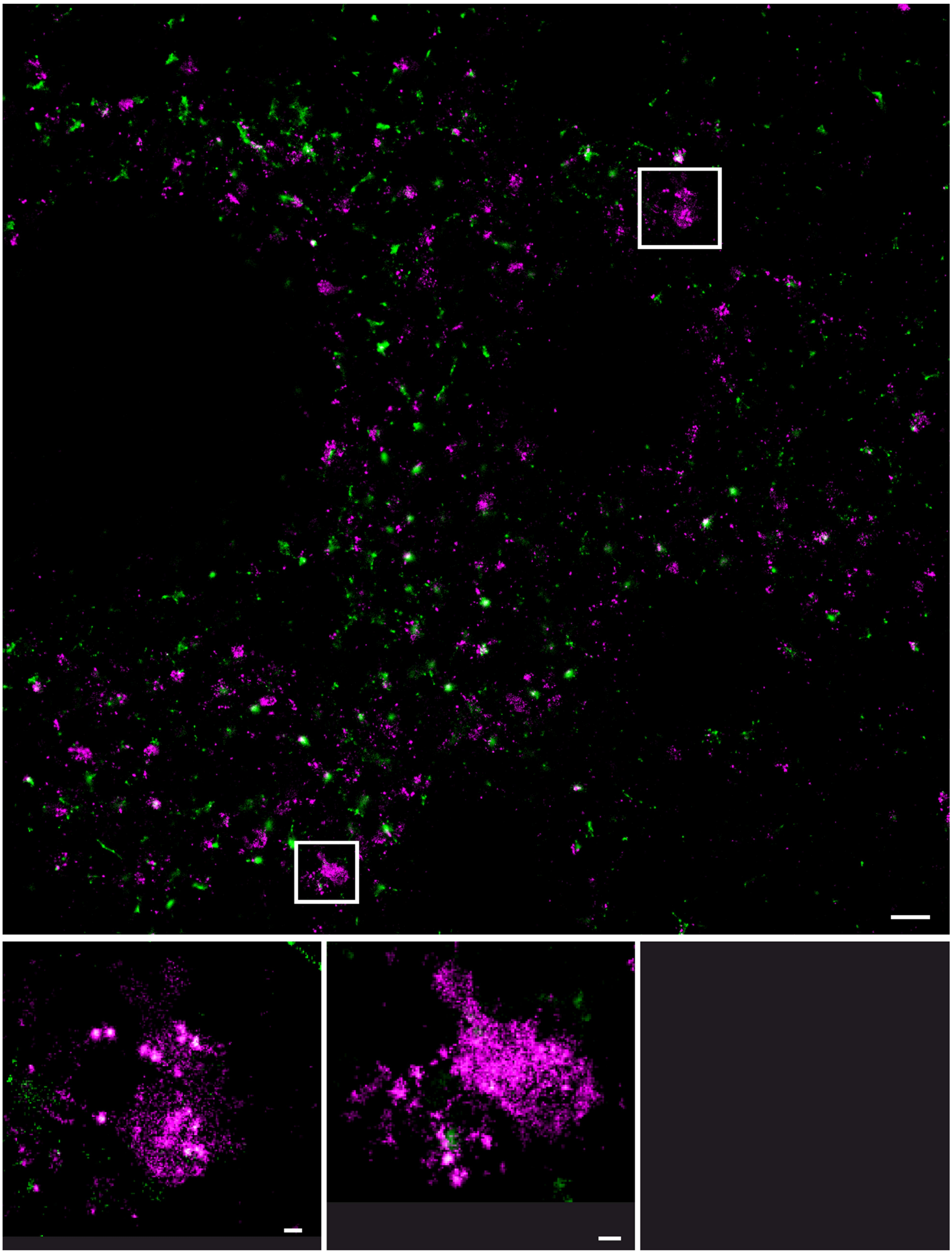

Figure S14. Partial cellular overview of SMLM data in an adipocyte with ROIs indicating endosomes presented in Figure 5 D. LNP-Cy5-mRNA (magenta), Transferrin (green), EGF (cyan). Scale bars 5µm (top), 100nm (bottom).

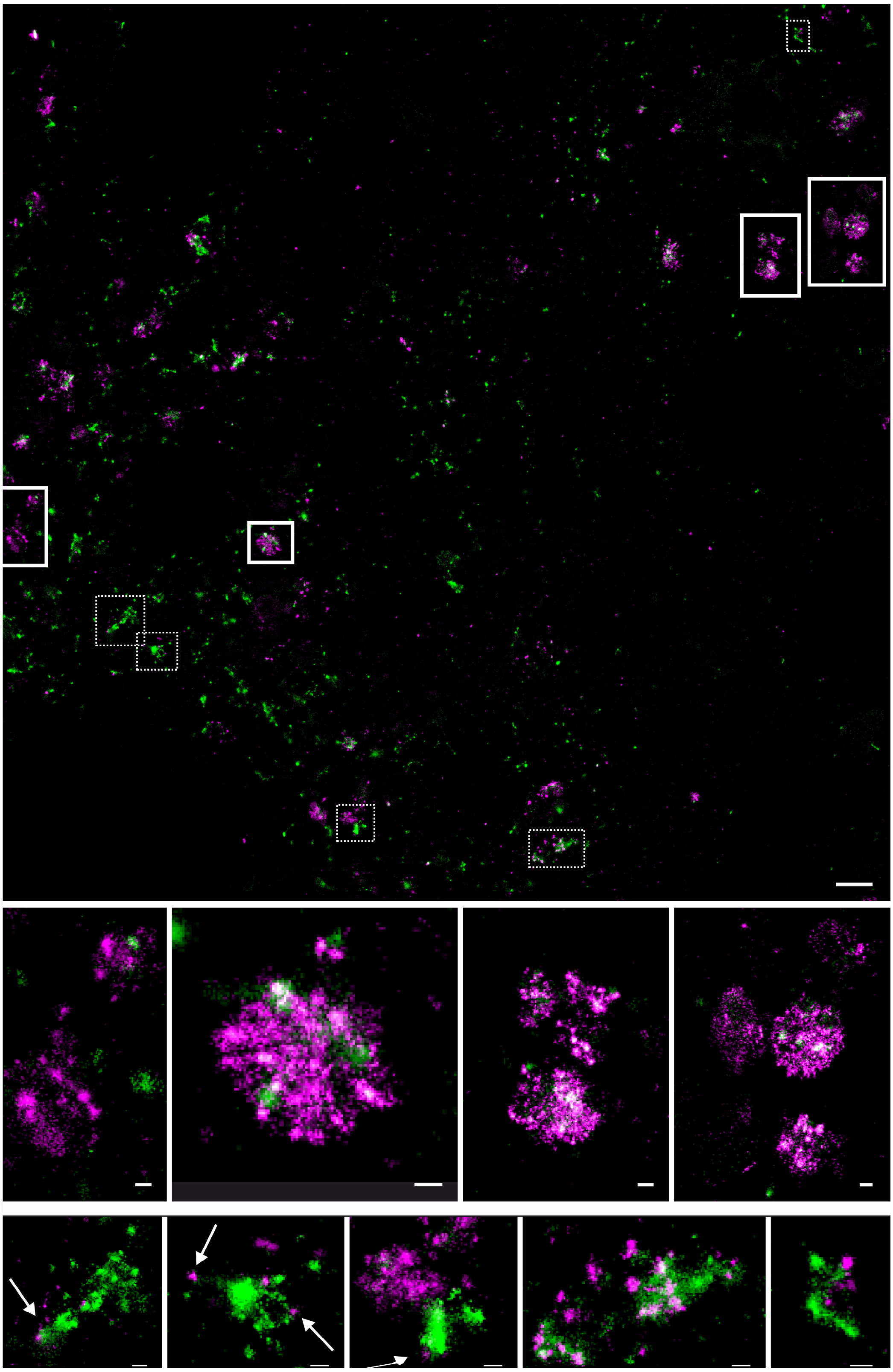

Figure S15. Partial cellular overview of SMLM data in an adipocyte with ROIs indicating endosomes presented in Figure 5 D and 6 D. LNP-Cy5-mRNA (magenta), Transferrin (green), EGF (cyan). Scale bars 5µm (top), 100nm (bottom)

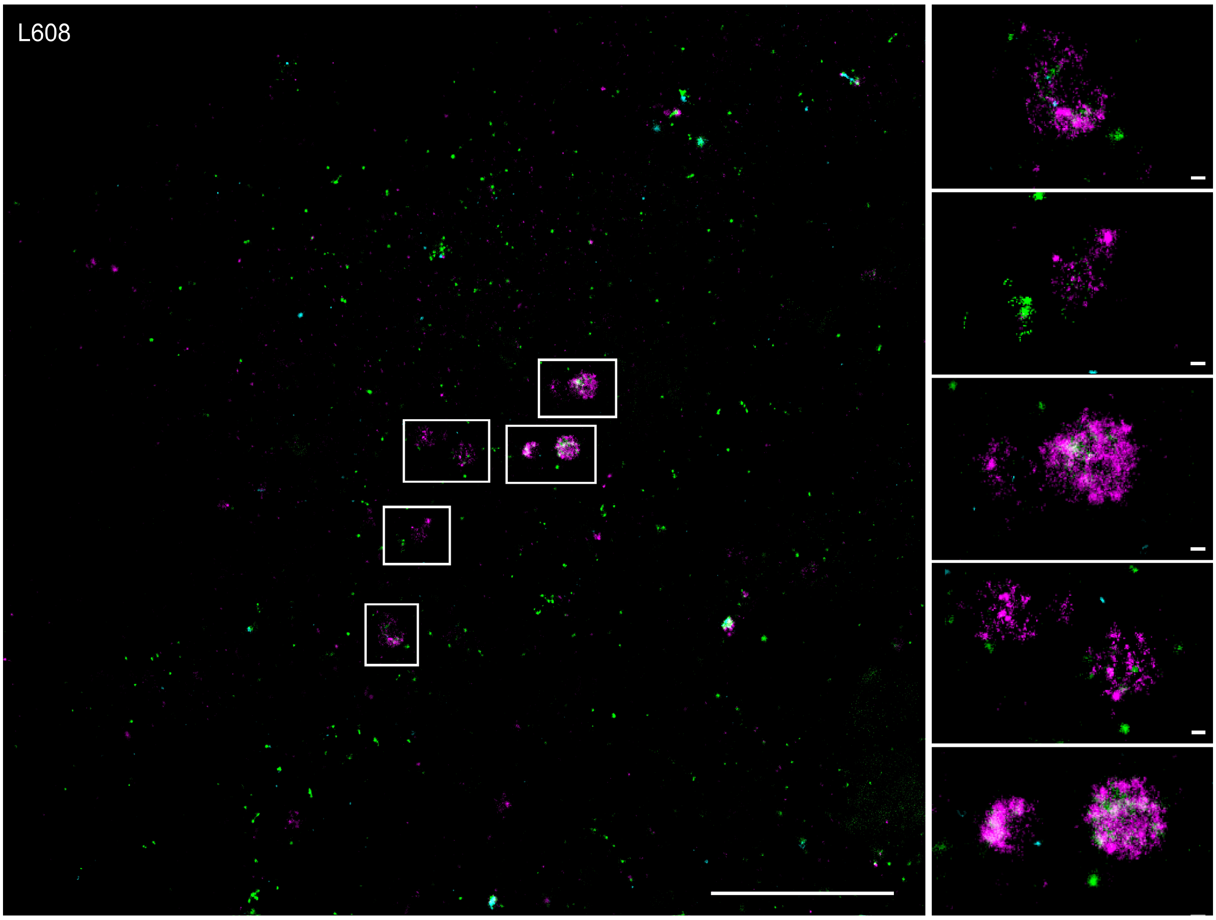

Figure S16. Partial cellular overview of SMLM data in an adipocyte with ROIs indicating additional examples of arrested endosomes for the L608 LNP formulation. LNP-Cy5-mRNA, (magenta), Transferrin (green), EGF (cyan). Scale bars 5 µm (left), 100nm (right).

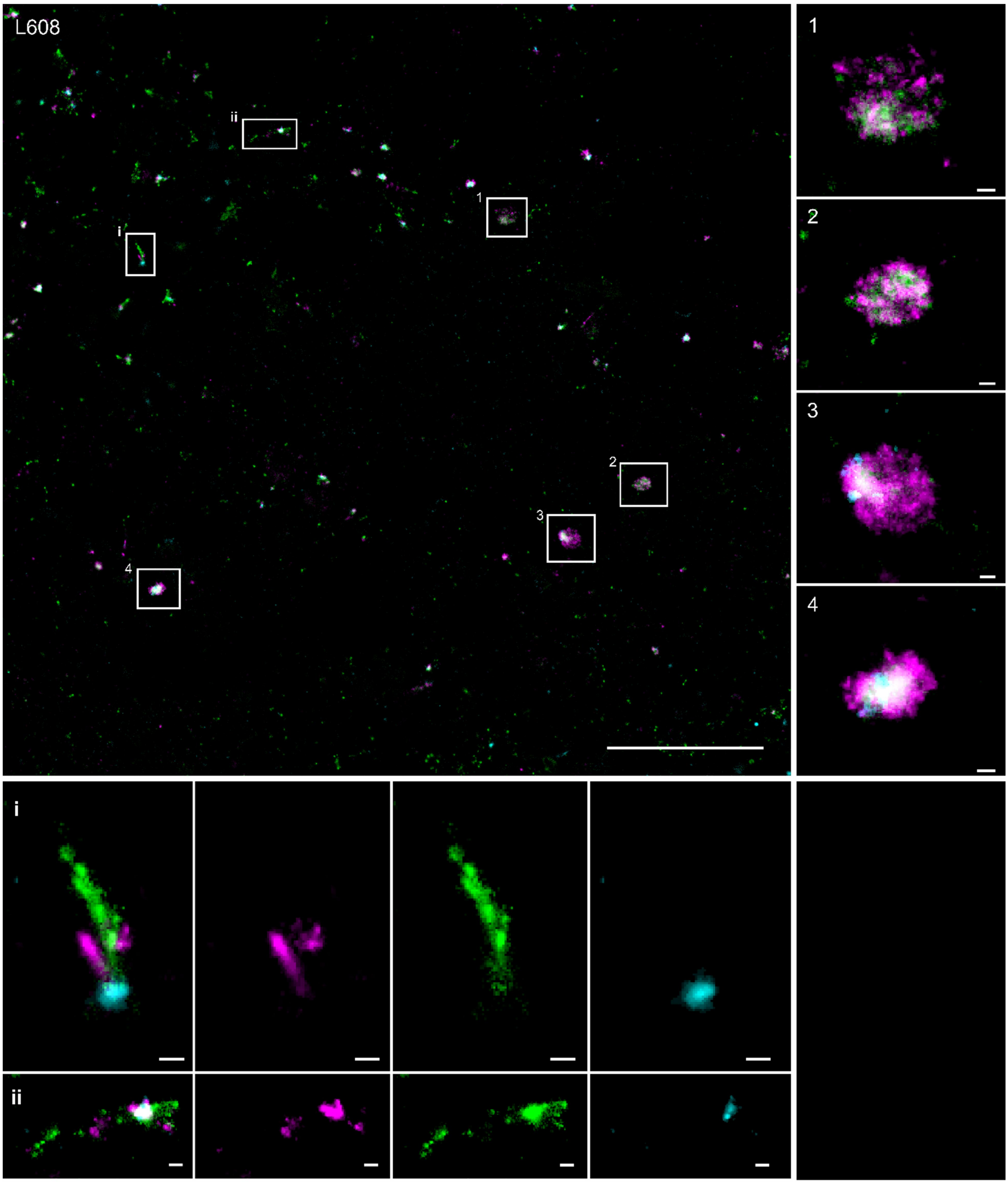

Figure S17. Partial cellular overview of SMLM data in an adipocyte with ROIs indicating additional examples of arrested endosomes (right panel) and possible mRNA escape events (bottom panel) for the L608 LNP formulation. LNP-Cy5-mRNA (magenta), Transferrin (green), EGF (cyan). Scale bars 5 µm (top left), 100nm (bottom, right).

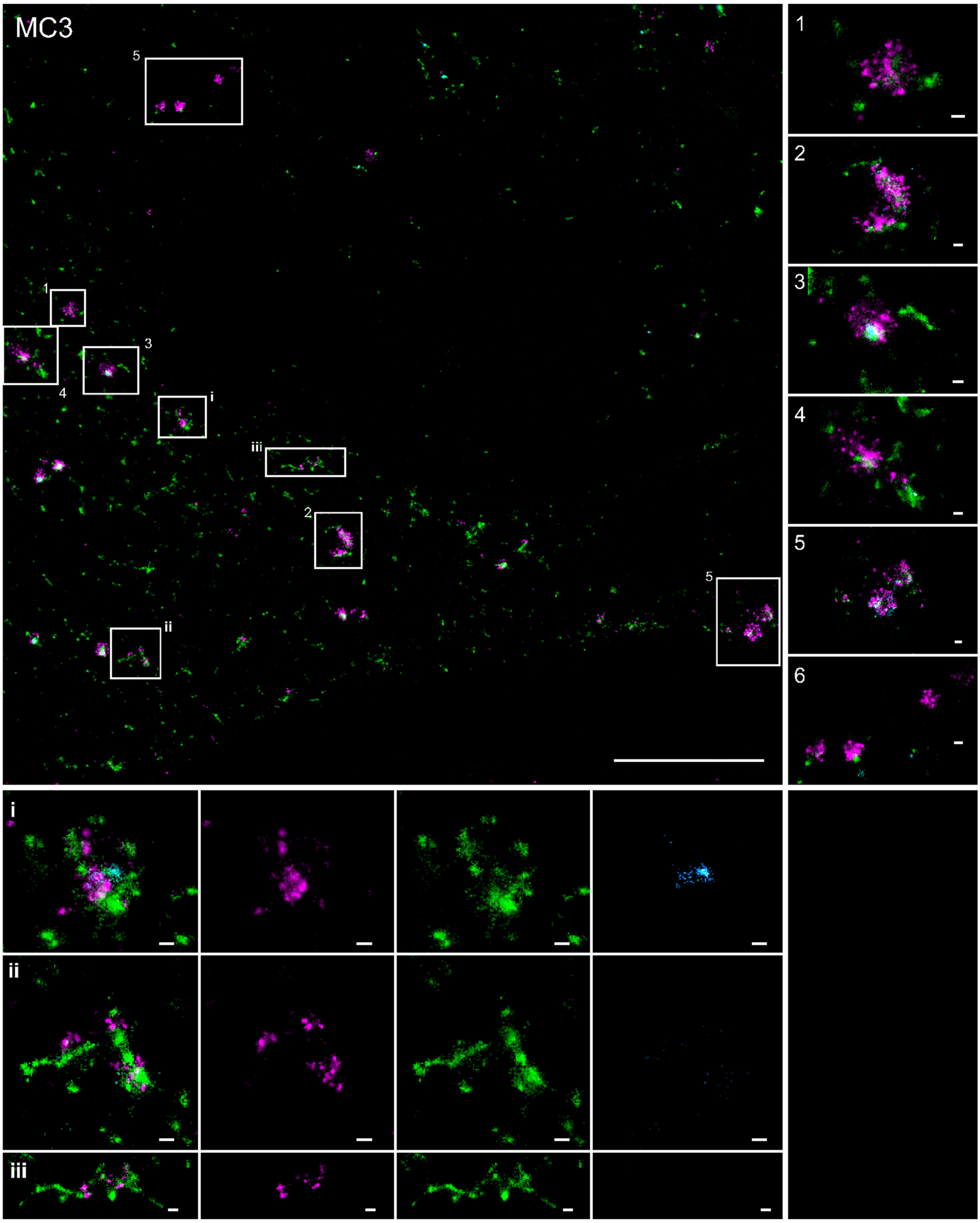

Figure S18. Partial cellular overview of SMLM data in an adipocyte with ROIs indicating additional examples of arrested endosomes (right panel) and possible mRNA escape events (bottom panel) for the MC3 LNP formulation. LNP-Cy5-mRNA (magenta), Transferrin (green), EGF (cyan). Scale bars 5 µm (top left), 100nm (bottom, right).

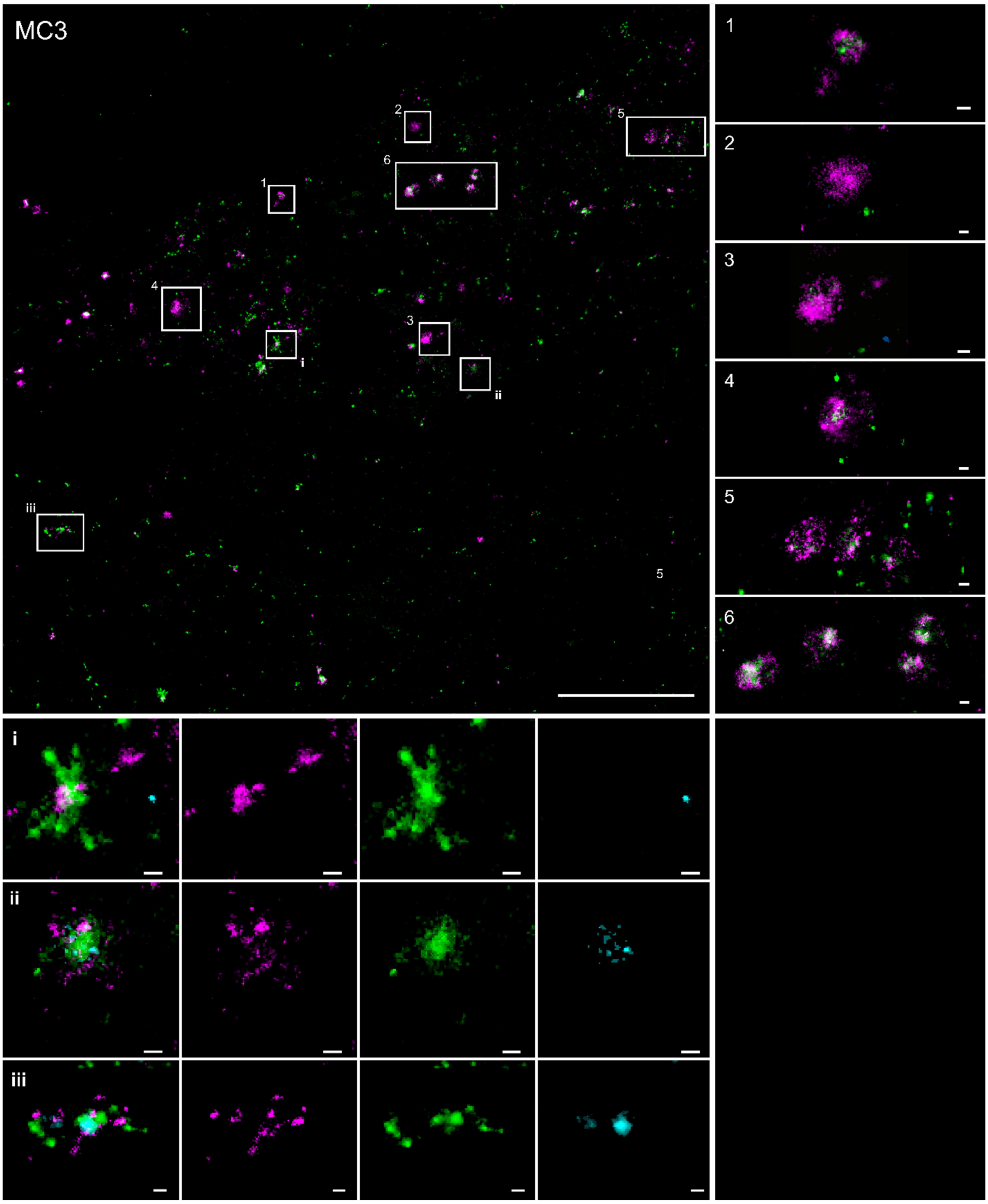

Figure S19. Partial cellular overview of SMLM data in an adipocyte with ROIs indicating additional examples of arrested endosomes (right panel) and possible mRNA escape events (bottom panel) for the MC3 LNP formulation. LNP-Cy5-mRNA (magenta), Transferrin (green), EGF (cyan). Scale bars 5 µm (top left), 100nm (bottom, right).

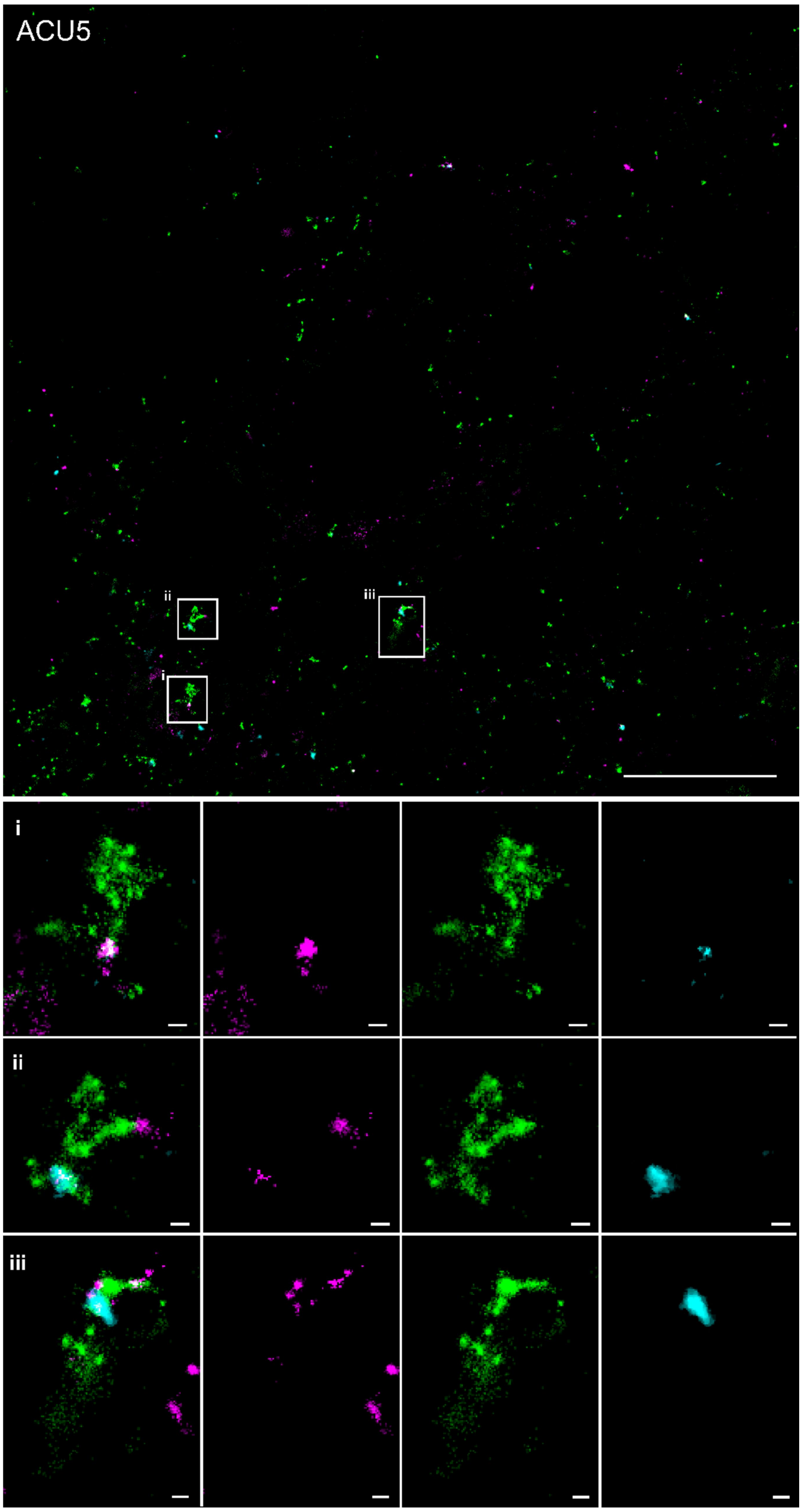

Figure S20. Partial cellular overview of SMLM data in an adipocyte with ROIs indicating additional examples of possible mRNA escape events (bottom panel) for the ACU5 LNP formulation. LNP-Cy5-mRNA (magenta), Transferrin (green), EGF (cyan). Scale bars 5 µm (top), 100nm (bottom).

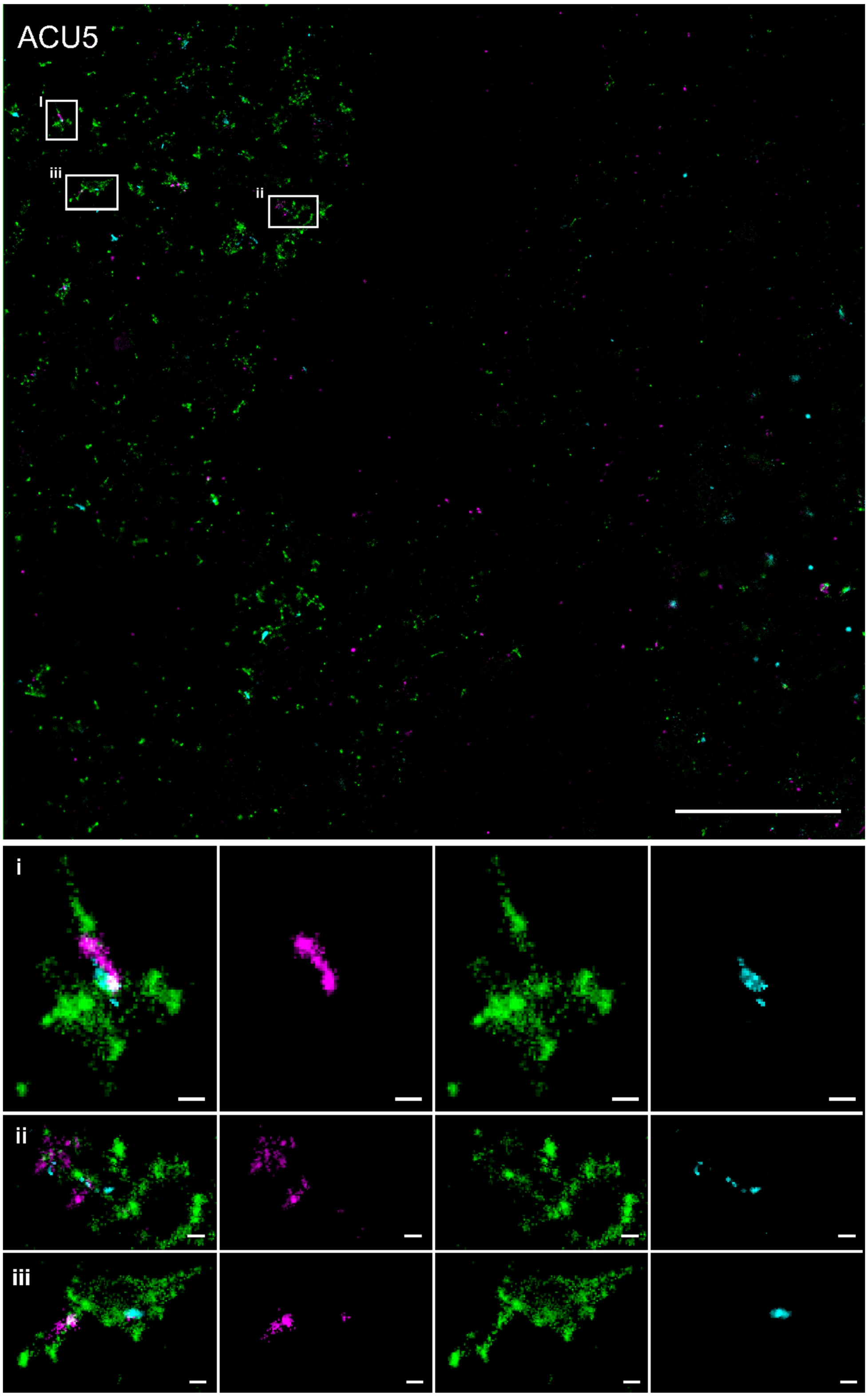

Figure S21. Partial cellular overview of SMLM data in an adipocyte with ROIs indicating additional examples of possible mRNA escape events (bottom panel) for the ACU5 LNP formulation. LNP-Cy5-mRNA (magenta), Transferrin (green), EGF (cyan). Scale bars 5 µm (top), 100nm (bottom).

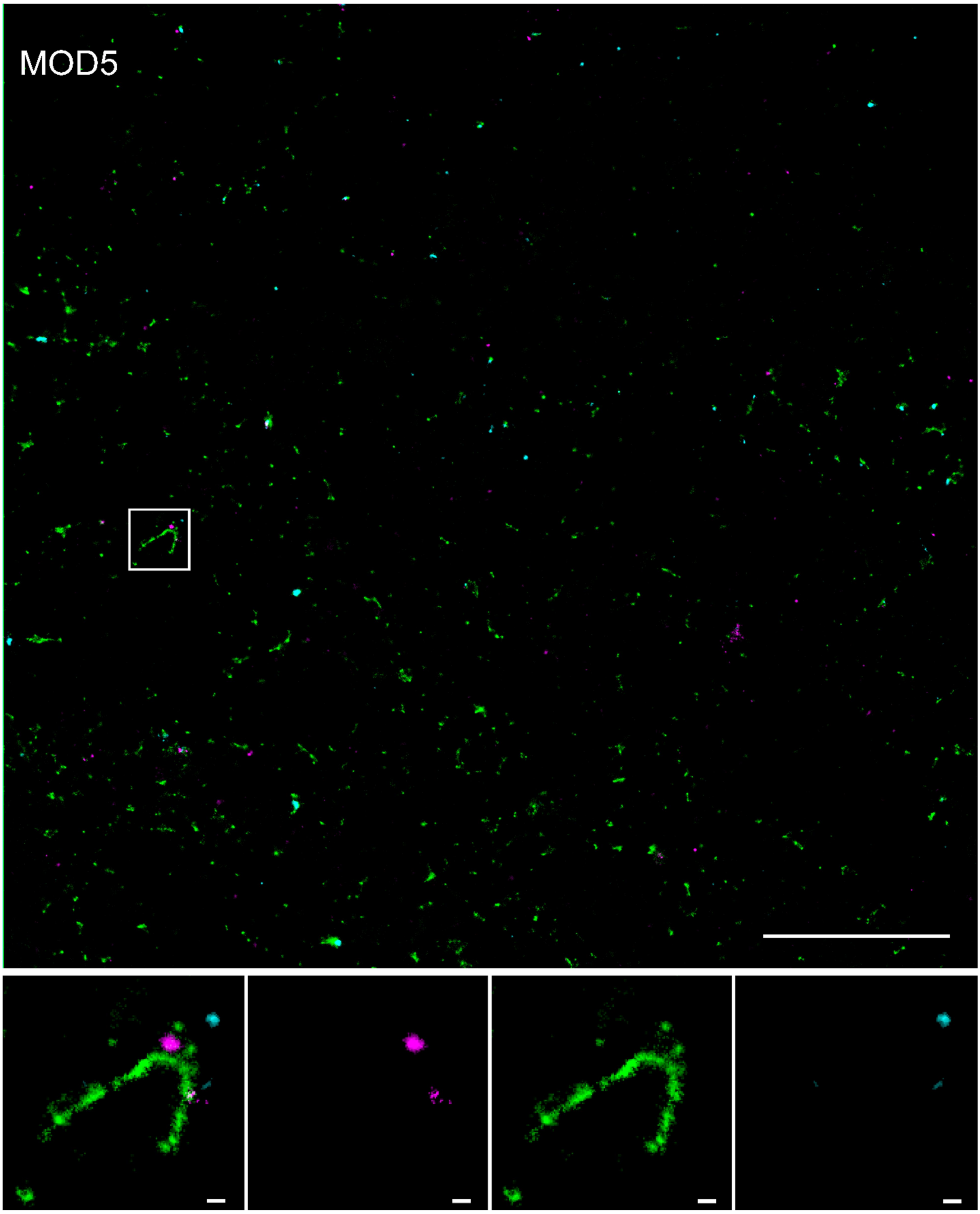

Figure S22. Partial cellular overview of SMLM data in an adipocyte with ROIs indicating additional examples of possible mRNA escape events (bottom panel) for the MOD5 LNP formulation. LNP-Cy5-mRNA (magenta), Transferrin (green), EGF (cyan). Scale bars 5 µm (top), 100nm (bottom).

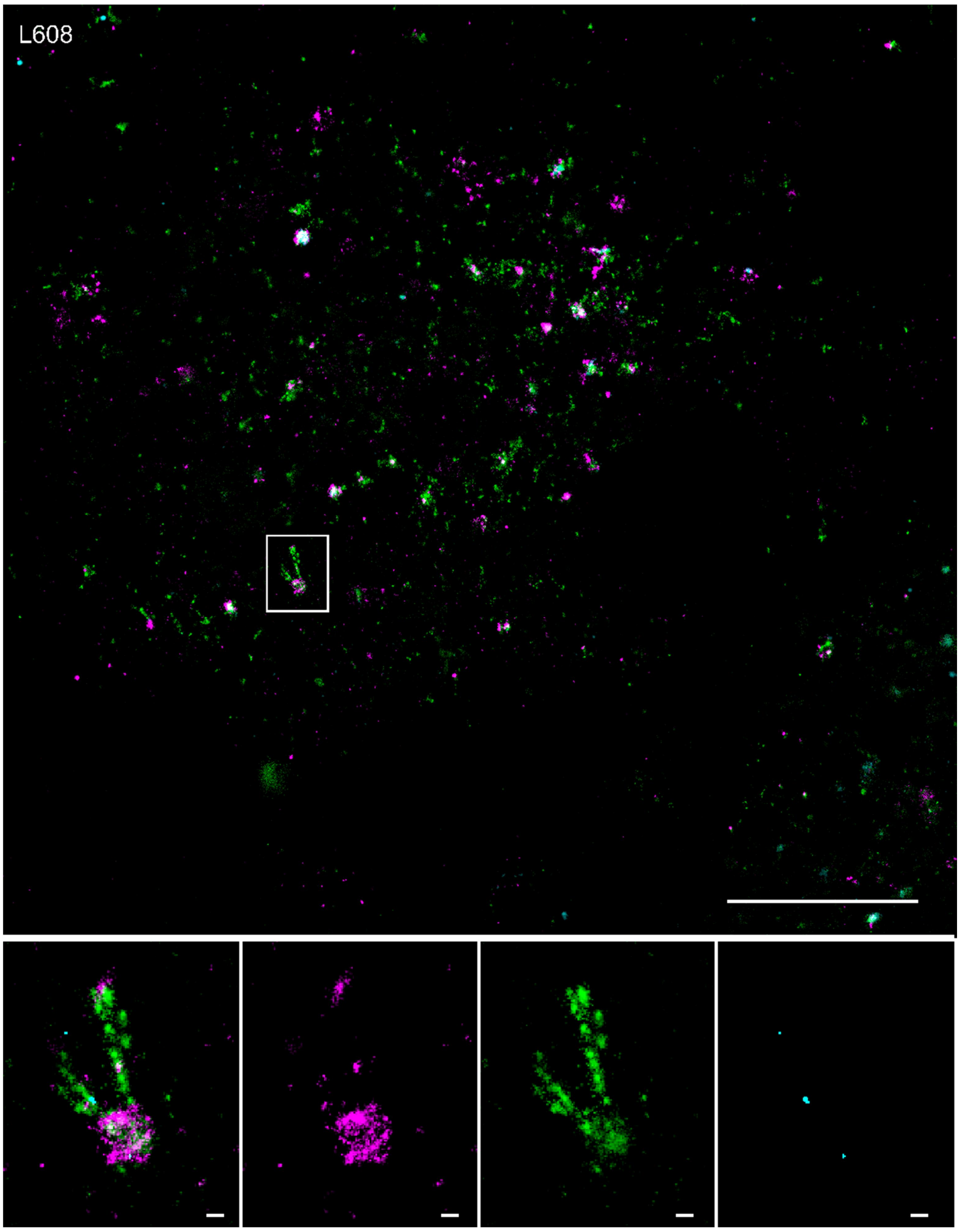

Figure S23. Partial cellular overview of SMLM data in an adipocyte with ROIs indicating additional examples of possible mRNA escape events (bottom panel) for the L608 LNP formulation. LNP-Cy5-mRNA (magenta), Transferrin (green), EGF (cyan). Scale bars 5 µm (top), 100nm (bottom).

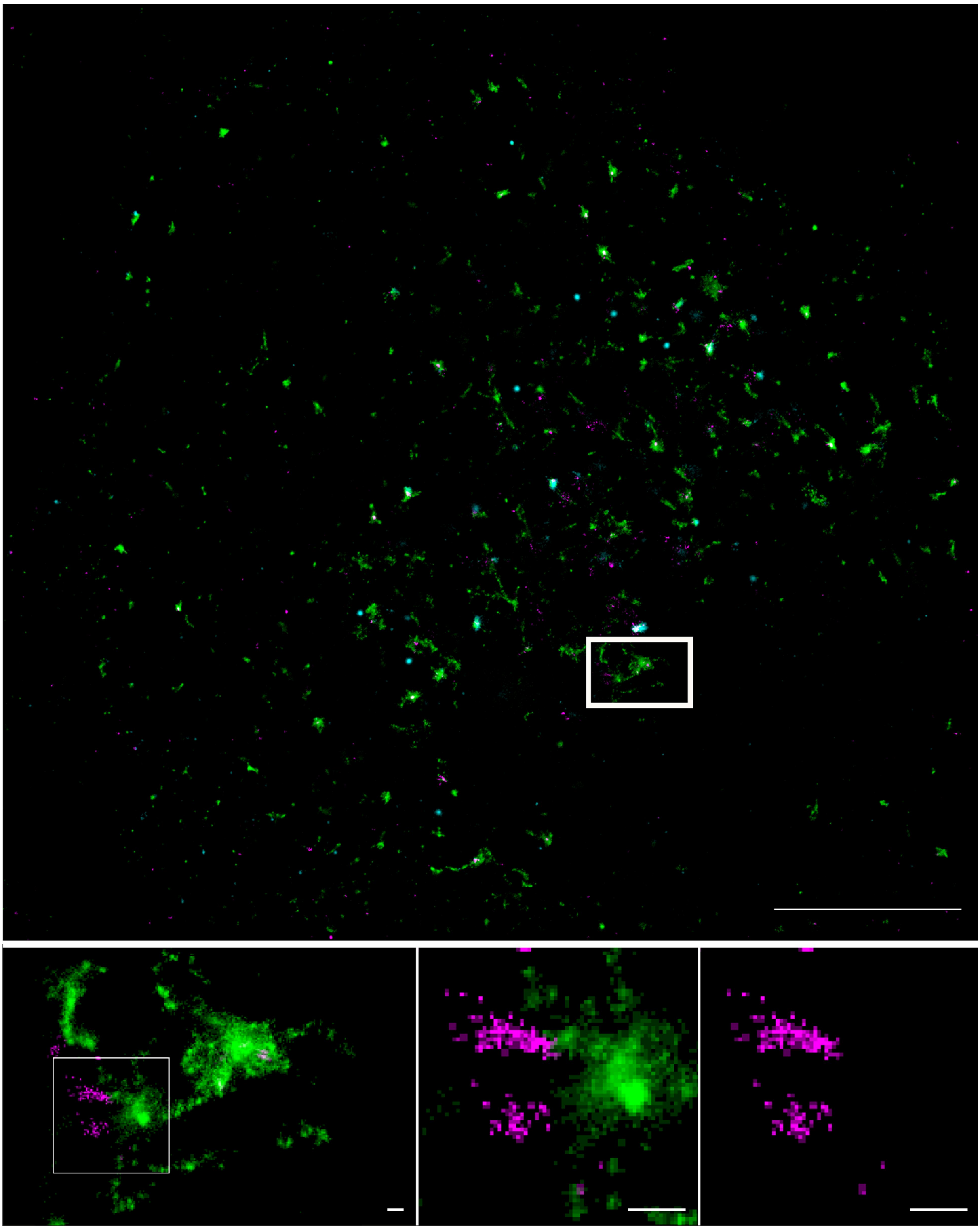

Figure S24. Partial cellular overview of SMLM data in a HeLa cell with ROIs indicating the endosome presented in Figure 6 B. LNP-Cy5-mRNA (magenta), Transferrin (green), EGF (cyan). Scale bars 5 µm (top), 100nm (bottom).

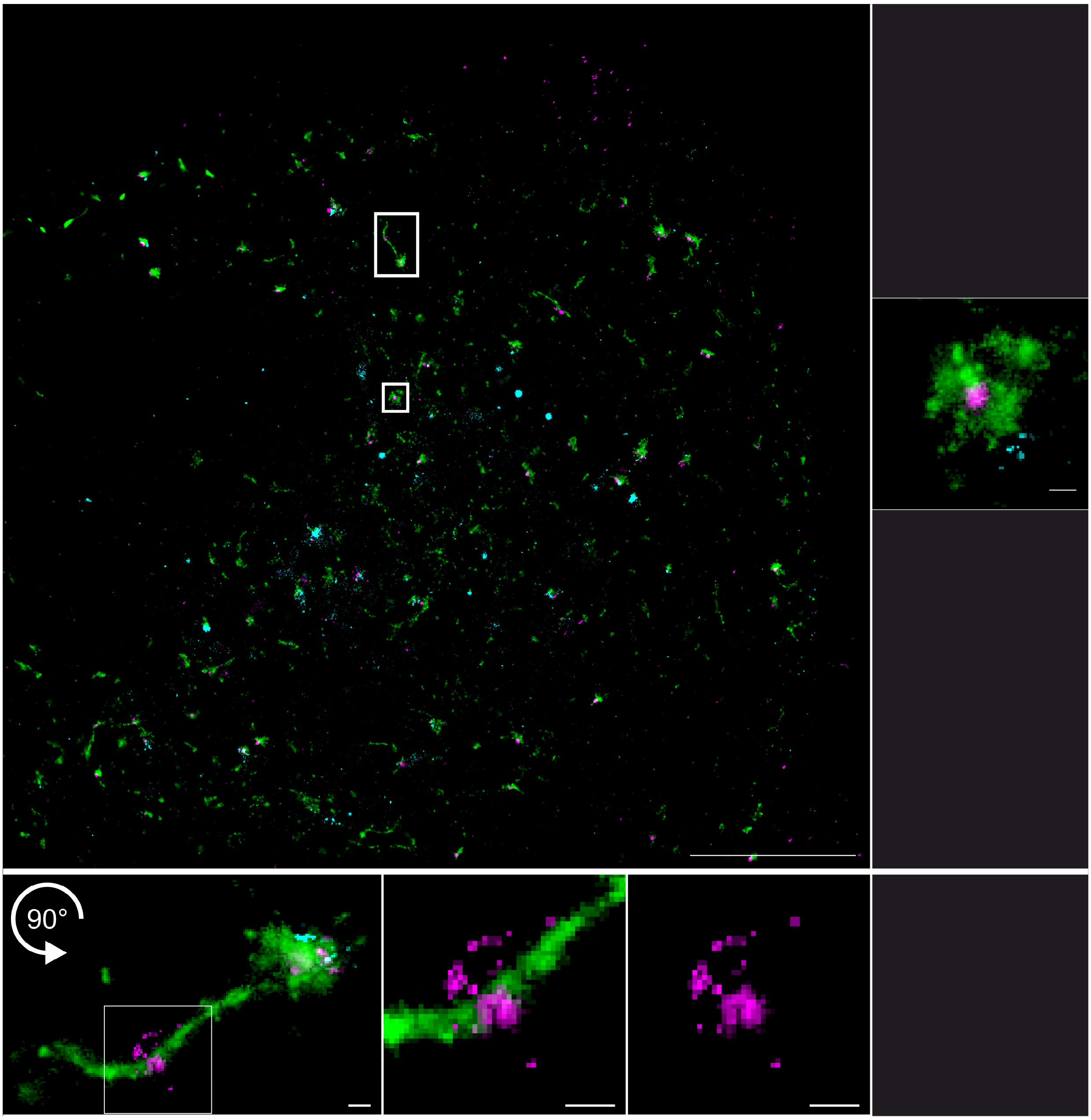

Figure S25. Partial cellular overview of SMLM data in a HeLa cell with ROIs indicating the endosome presented in Figure 4 C and Figure 6 C. LNP-Cy5-mRNA (magenta), Transferrin (green), EGF (cyan). Scale bars 5 µm (top), 100nm (bottom).

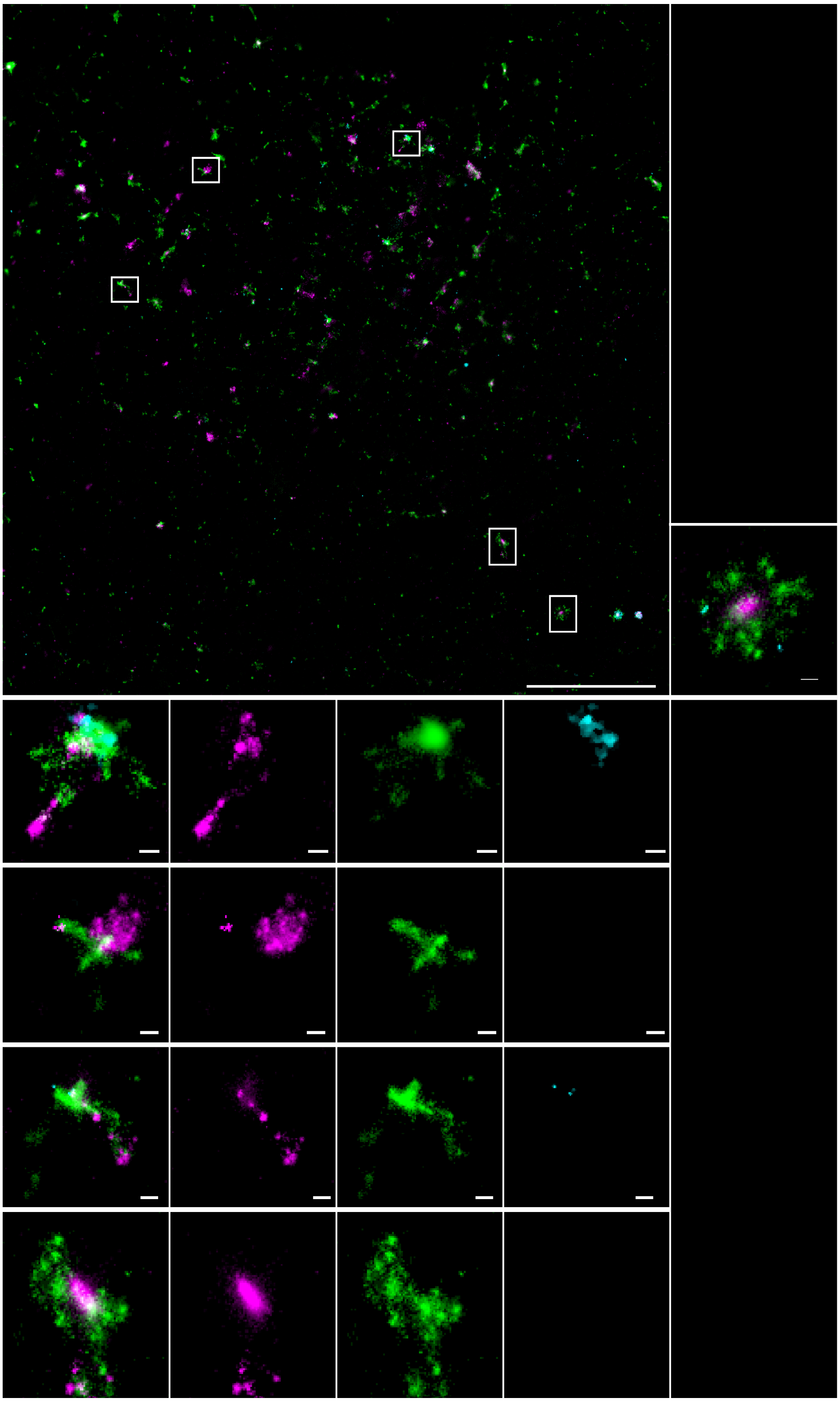

Figure S26. Partial cellular overview of SMLM data in a HeLa cell with ROIs indicating additional examples of possible mRNA escape events (bottom panel) for the MC3 LNP formulation and ROI for image presented in Figure 4 B. LNP-Cy5-mRNA (magenta), Transferrin (green), EGF (cyan). Scale bars 5µm (top), 100nm (bottom).

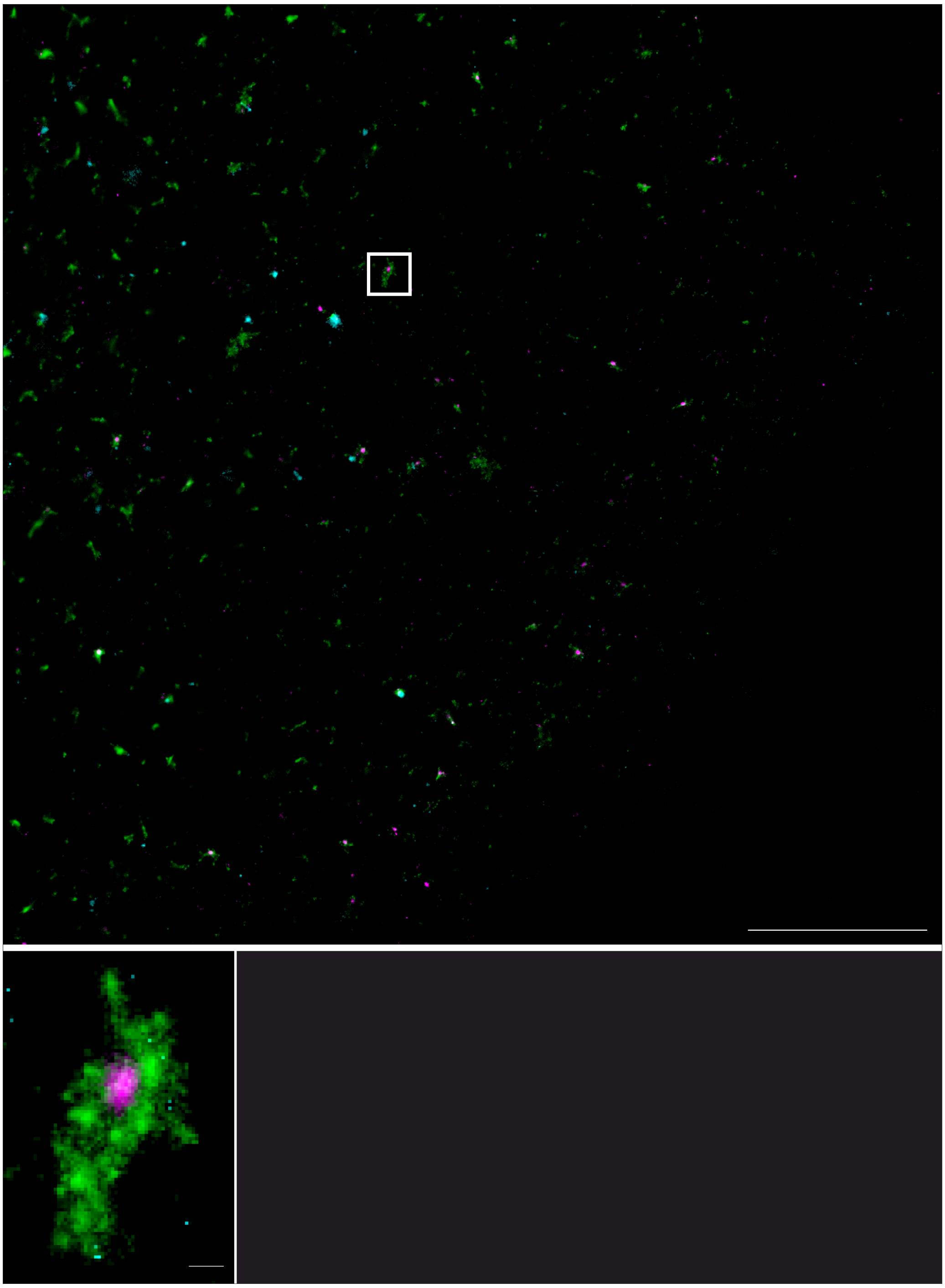

Figure S27: Partial cellular overview of SMLM data in HeLa cells with ROIs indicating the endosome presented in Figure 4 D. LNP-Cy5-mRNA (magenta), Transferrin (green), EGF (cyan). Scale bars 5µm (top), 100nm (bottom).

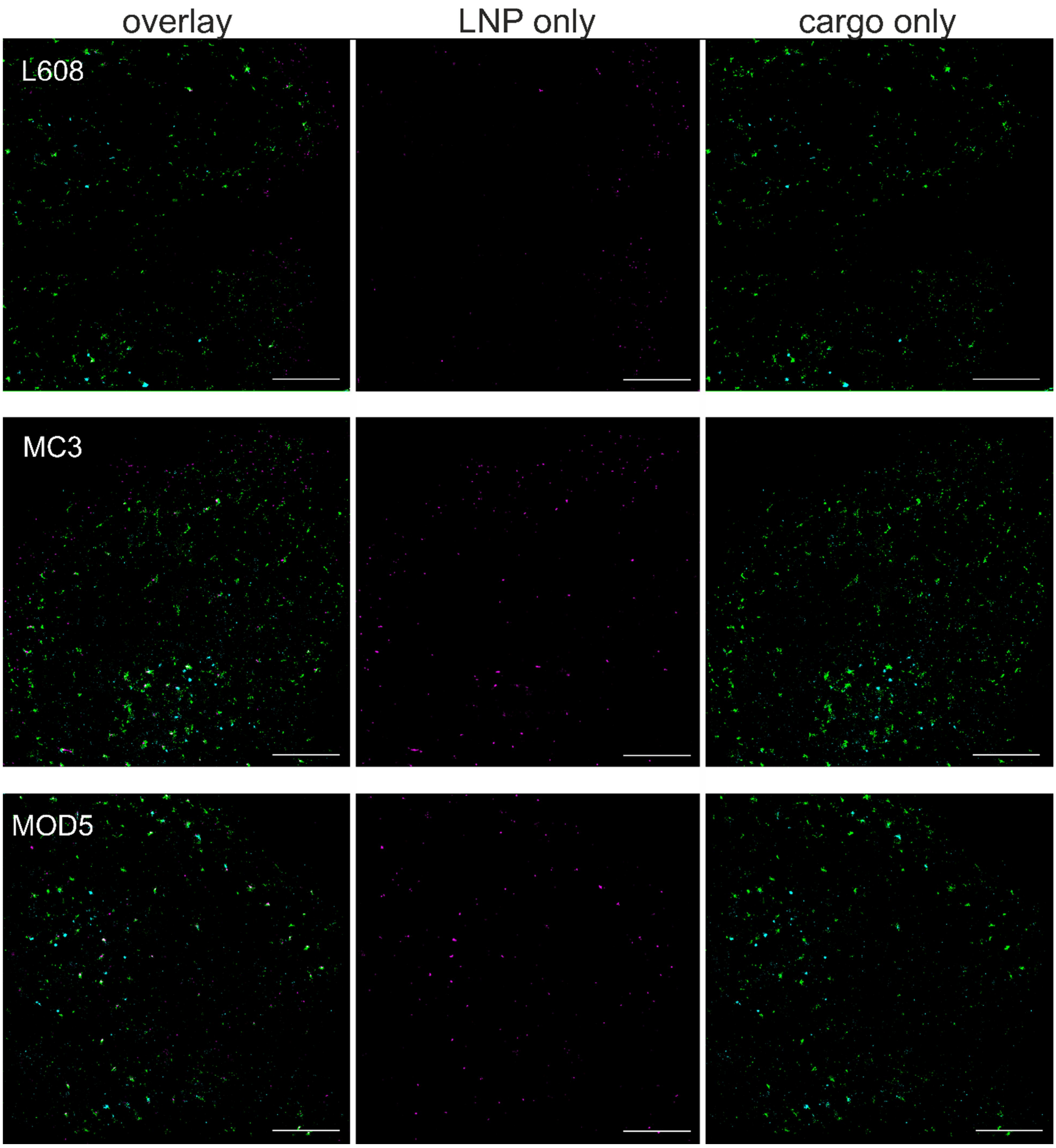

Figure S28. Exemplary field of views of cells incubated with LNPs and cargo molecules simultaneously for 30 minutes. LNPs (magenta) are distributed almost exclusively at the cellular periphery, while Transferrin (green) and EGF (cyan) cargo is apparent within the entire cell. Scale bars 5µm

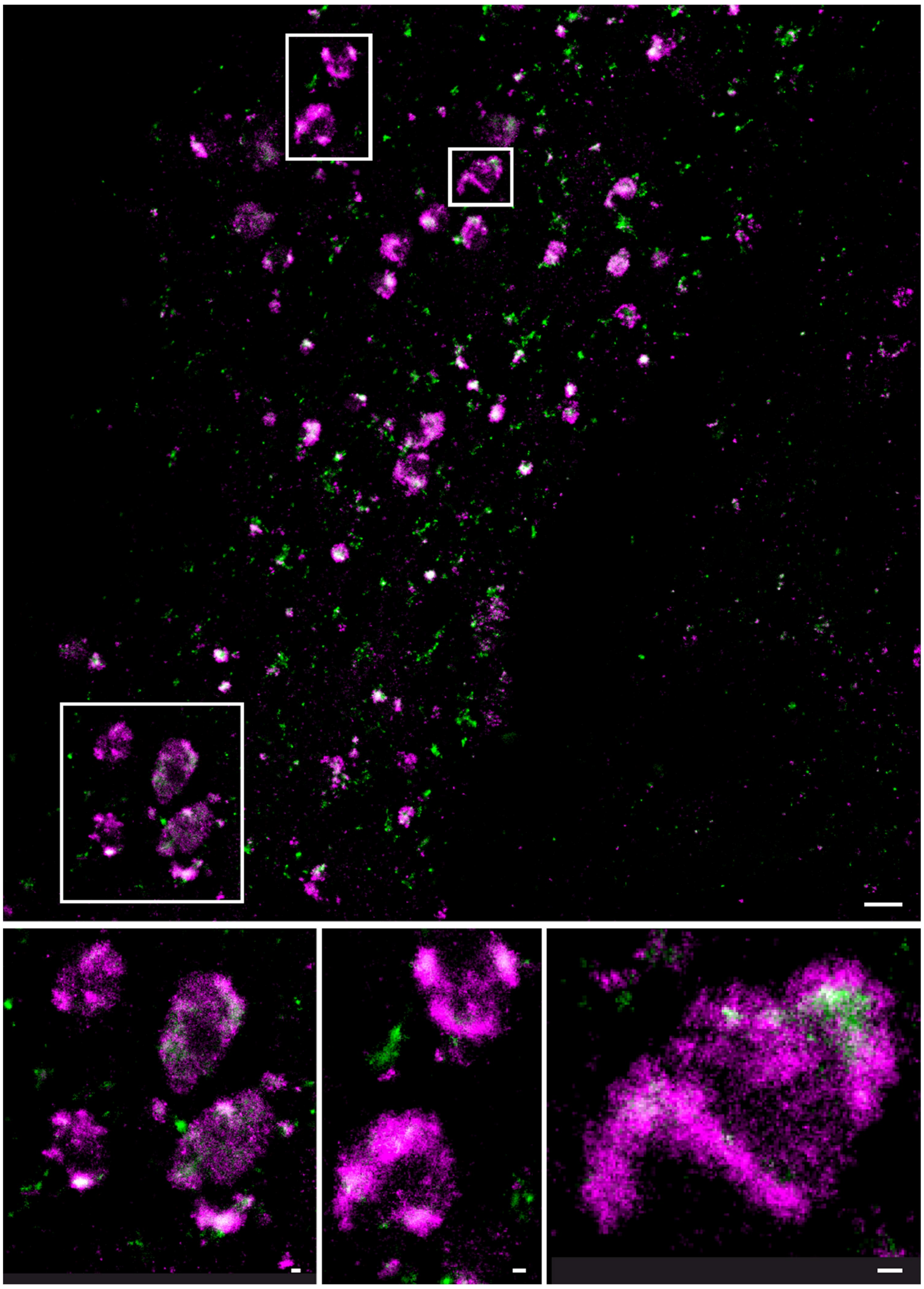

Figure S29: Arrested endosomes in primary fibroblasts. *Top:* Paradigmatic overview of a fibroblast, imaged by SMLM (Tfn (green) and LNP-Cy5-mRNA (red), Scale bars 1µm). White boxes indicate exemplary arrested endosomes. *Bottom:* Zoom-ins of regions of interest for arrested endosomes. Scale bars 100 nm.

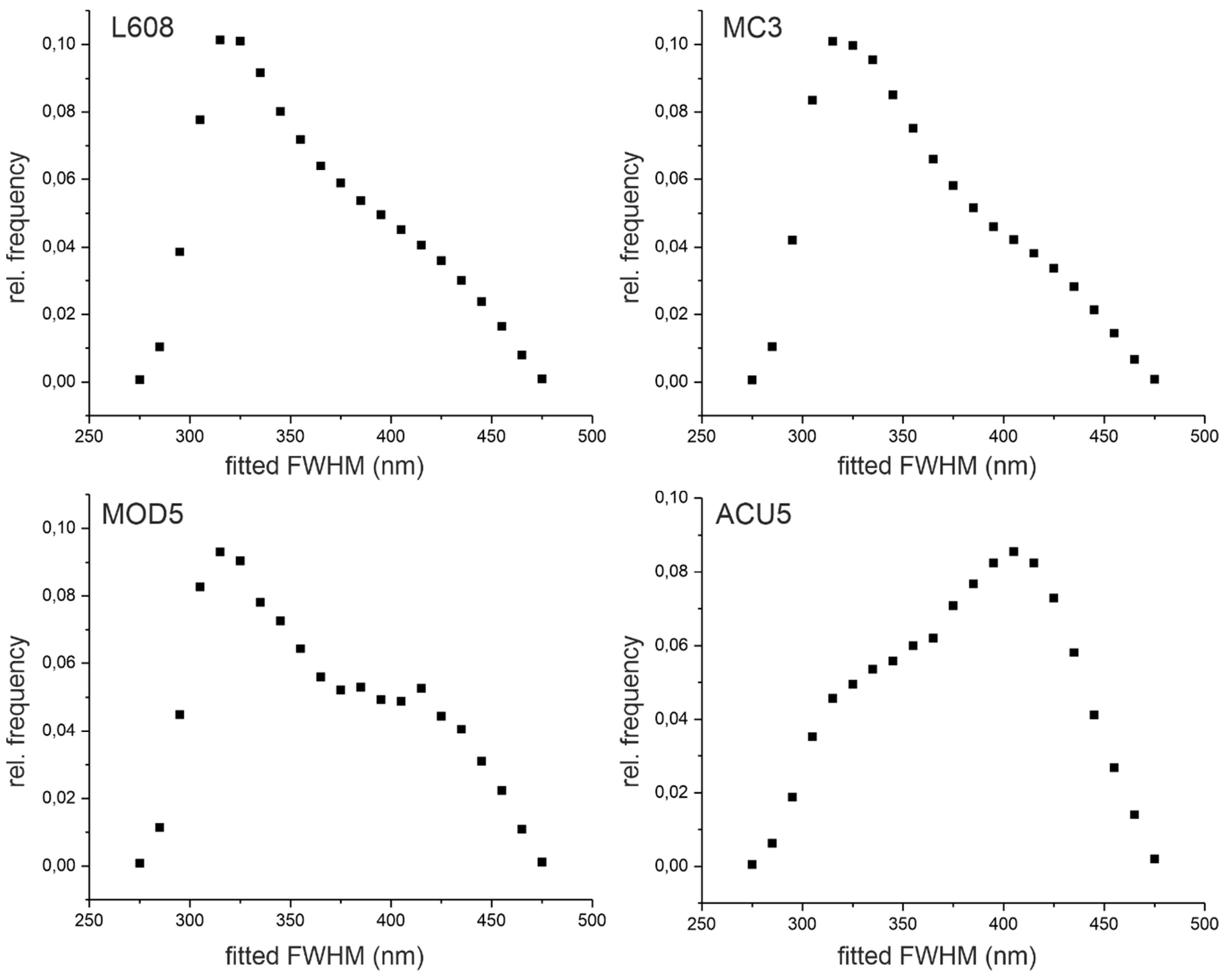

Figure S30. Distribution of fitted FWHM of mRNA-Cy5 localizations. The main portion of all localizations reside in a window between 325 nm and 425 nm FWHM, which indicates an axial distribution in an approximately 500 nm.

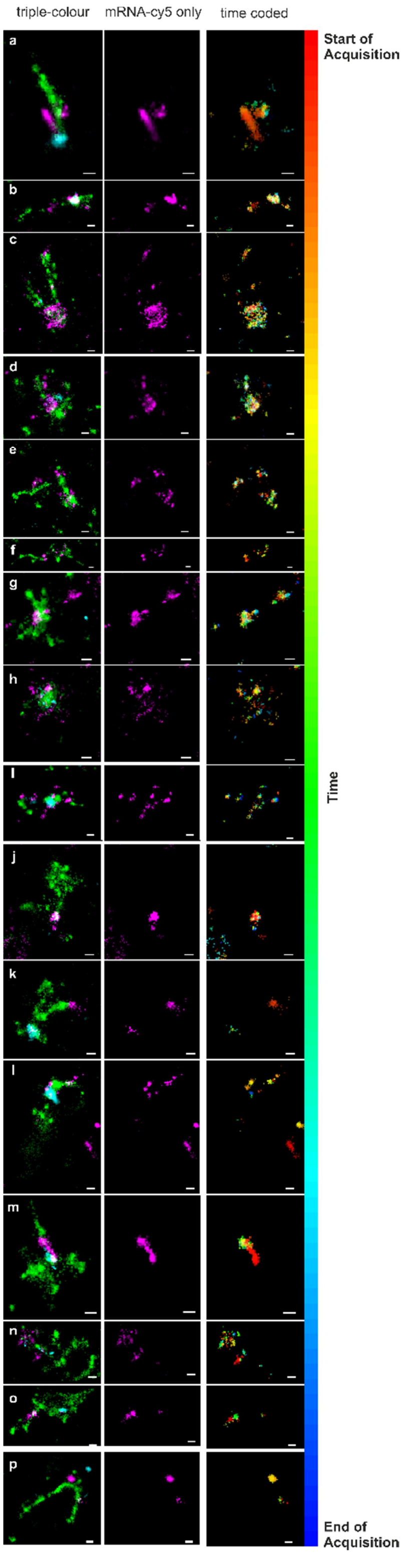

Figure S31. The temporally color-coded images of mRNA escape events (right coloumn) show no sign of directed spatio-temporal displacements (drift or diffusion artefacts). Therefore, the potential escape events are genuine mRNA distributions in the sample. Scale bars 100 nm.

L608 AZ14245376 EN11819-93-001

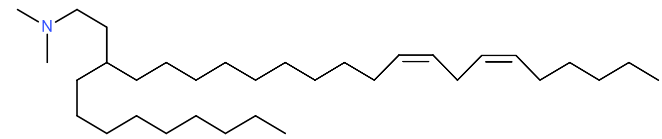

^{1}H NMR (500 MHz, $CDCl_3$) δ 5.36 (tdd, 4H), 2.77 (t, 2H), 2.39 (d, 8H), 2.05 (q, 4H), 1.50 (q, 2H), 1.19 – 1.38 (m, 39H), 0.89 (td, 6H).

LC-MS (ESI) : 476.6 $[M + H]^+$

MC3 AZ13759693 EN08699-26-001

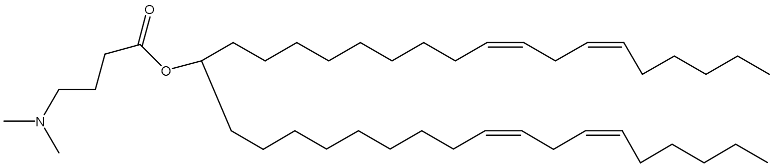

^{1}H NMR (500 MHz, $CDCl_3$) δ 5.28 – 5.43 (m, 8H), 4.74 – 4.9 (m, 1H), 2.77 (t, 4H), 2.3 – 2.37 (m, 4H), 2.26 (s, 6H), 2.04 (qd, 8H), 1.81 (p, 2H), 1.50 (d, 4H), 1.24 – 1.4 (m, 36H), 0.89 (t, 6H).

LC-MS (ESI) : 642.8 $[M + H]^+$

ACU5 AZ13851985 EN09575-30-001

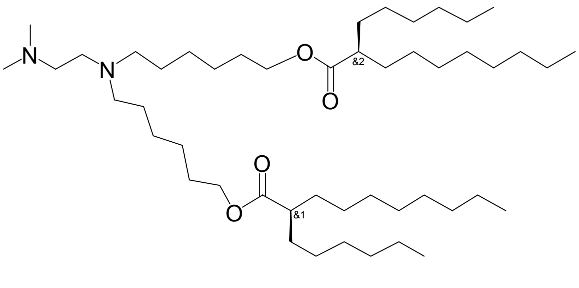

^{1}H NMR (500 MHz, $CDCl_3$) δ 4.05 (t, 4H), 2.52 – 2.58 (m, 2H), 2.40 (dt, 5H), 2.30 (tt, 2H), 2.25 (s, 6H), 1.52 – 1.66 (m, 8H), 1.22 – 1.47 (m, 57H), 0.87 (td, 12H).

LC-MS (ESI) : 765.5 $[M + H]^+$

ACU22 AZ14028367 EN08257-22-001

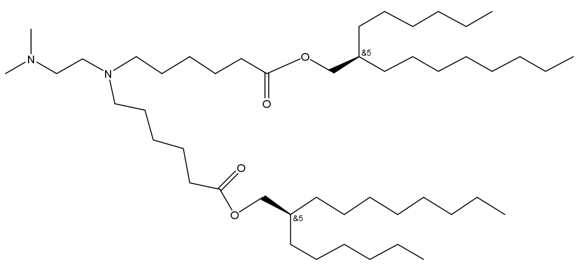

^{1}H NMR (500 MHz, $CDCl_3$) δ 3.96 (d, 4H), 2.49 – 2.58 (m, 2H), 2.26 – 2.44 (m, 10H), 2.23 (s, 6H), 1.6 – 1.68 (m, 6H), 1.4 – 1.49 (m, 4H), 1.24 – 1.35 (m, 52H), 0.88 (t, 12H).

LC-MS (ESI) : 765.8 $[M + H]^+$

Mod5 (SM86) AZ14118365 EN08699-25-001

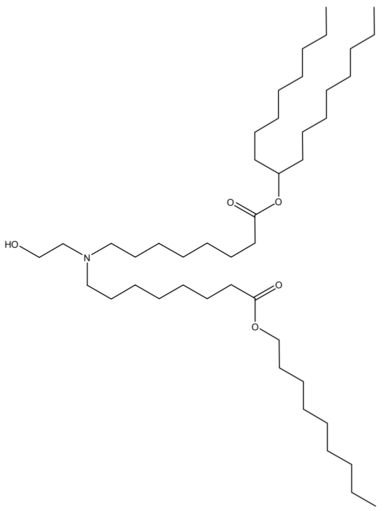

^{1}H NMR (500 MHz, $CDCl_3$) δ 4.86 (p, 1H), 4.05 (t, 2H), 3.57 (t, 2H), 2.63 (s, 2H), 2.50 (s, 4H), 2.28 (td, 4H), 1.62 (q, 6H), 1.49 (dq, 8H), 1.18 – 1.34 (m, 49H), 0.88 (td, 9H).

LC-MS (ESI) : 710.8 $[M + H]^+$

L319 AZ13897989 CRO-TI-CVO-370-1-012-01

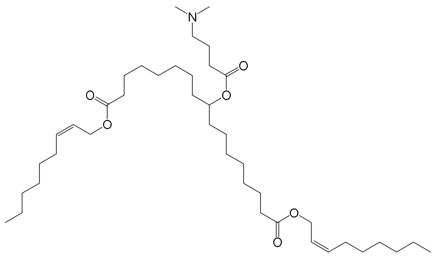

^{1}H NMR (400 MHz, DMSO-d6) δ 0.86 (t, 6H), 1.05 – 1.38 (m, 31H), 1.40 – 1.58 (m, 7H), 1.66 (p, 2H), 2.07 (q, 4H), 2.16 (s, 5H), 2.28 (q, 7H), 4.56 (d, 4H), 4.73 – 4.83 (m, 1H), 5.44 – 5.57 (m, 2H), 5.57 – 5.70 (m, 2H), 8.18 (s, 1H)

LC-MS (ESI) : 678.6 $[M + H]^{+}$

Figure S32: NMR characterization data of cationic lipids. (Page 33-35).

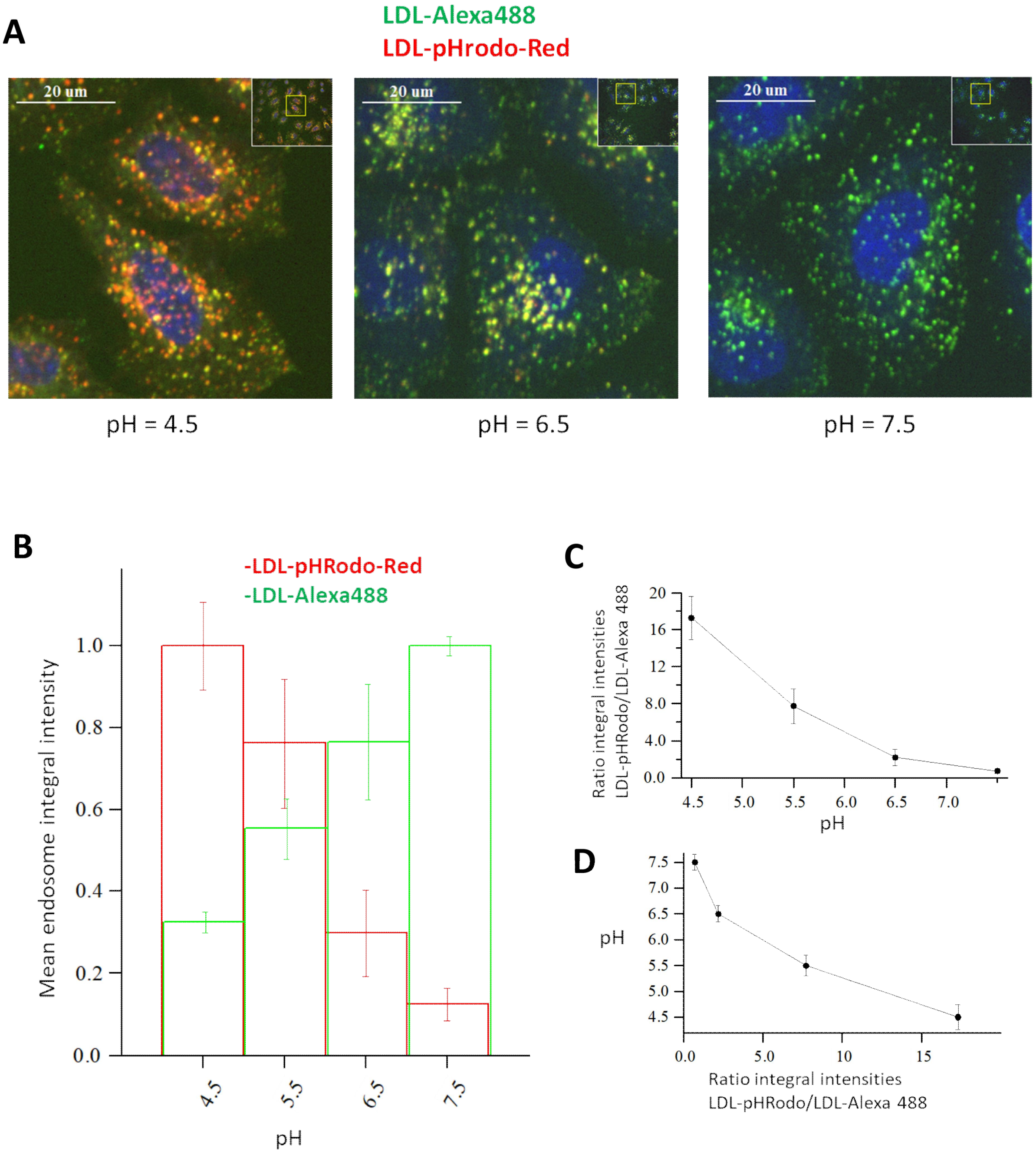

Figure S33. **(A)** Representative images of co-internalized mixture of LDL-pHRodo-Red/LDL-Alexa-488. Cells were fixed, washed, incubated in the calibration buffers of pH 4.5, 6.5 and 7.5 and imaged **(B)** Mean integral intensity of the endosomes positive for LDL-Alexa488 (green) and LDL-pHRodo (red) normalized on maximum value. Presented data were acquired in 3 independent experiments with 3 replicates per experiment.The mean number of endosomes per replicates ~74000±27000. **(C)** The ratio of integral intensities LDL-PHRodo per endosome to integral intensities LDL-Alexa488 per endosome. The LDL-pHRodo to LDL-Alexa-488 ratio for each endosome was calculated and then averaged first within each replicates, then between replicates, and finally between independent experiments. **(D)** Ratiometric calibration curve. Error bar denotes SEM. n = 3 independent experiments

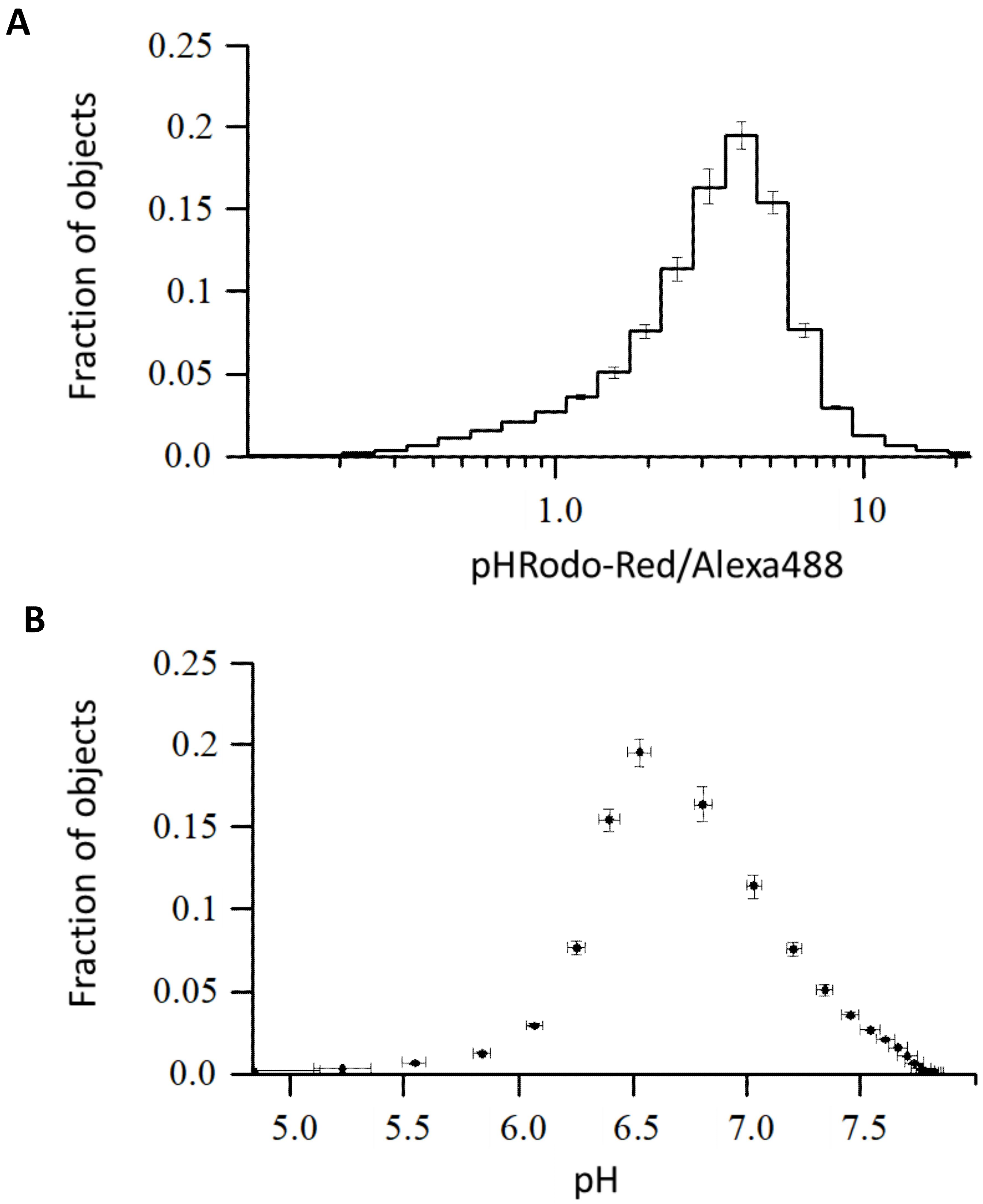

Figure S34. (A) Distribution of pH-Rodo-Red to Alexa 488 ratios that was measured for calibration (buffer pH=6.5). **(B)** Distribution of pH derived from the calibration curve presented in the Supplementary Fig. 33 D and the ratio distributions from Supplementary Fig. 34 A.

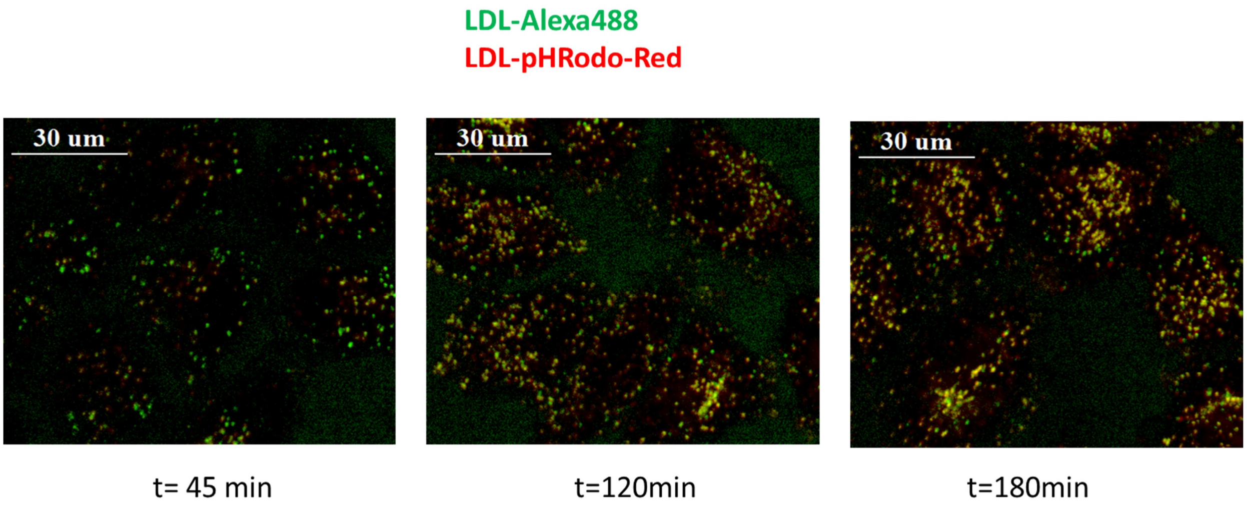

Figure S35: Representative images of co-internalized LDL-pHRodo-Red/LDL-Alexa-488. Cells were imaged live at 45, 120 and 180 min after cargo incubation.

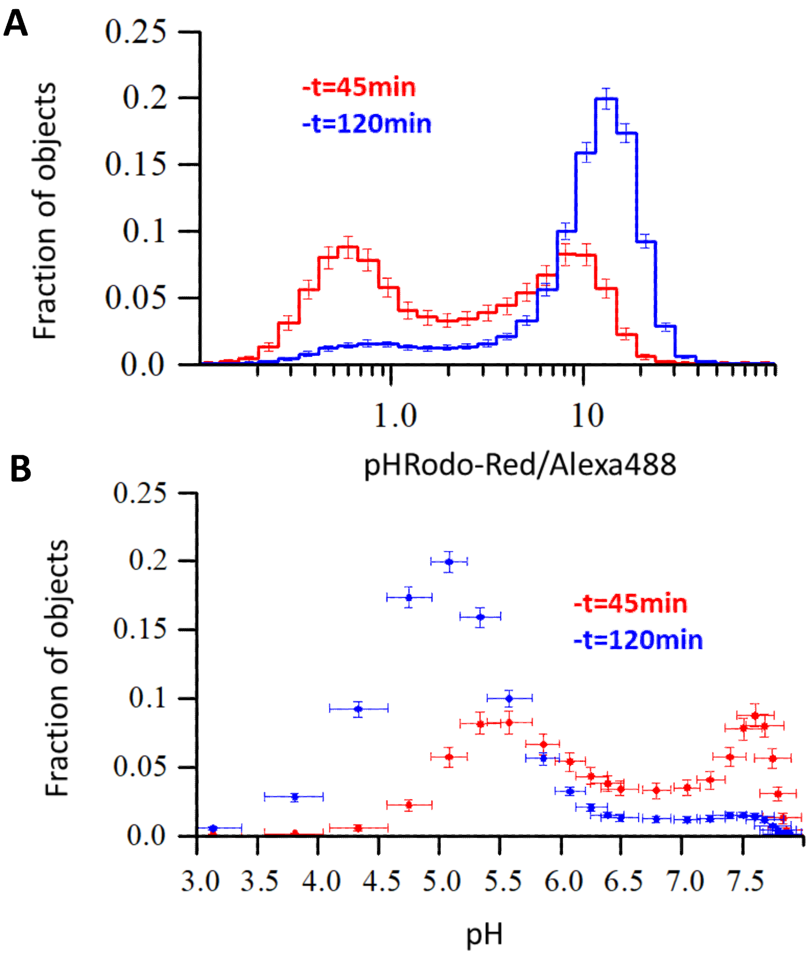

Figure S36: (A) Distribution of ratios that was measured in the kinetics experiment at t=45 min(red) and t=120min(blue) **(B)** Distribution of pH that was derived from calibration curve presented in Supplementary 33 D and distribution ratios in Supplementary Fig 36 A for t=45min(red) and t=120min

(blue). (**A**) Error bar denotes SEM between biological replicates (n = 3). (**B**) Vertical error bar denotes the SEM between biological replicates. Horizontal error bar calculated by error propagation as described in Methods (section **Ratiometric pH measurement**).

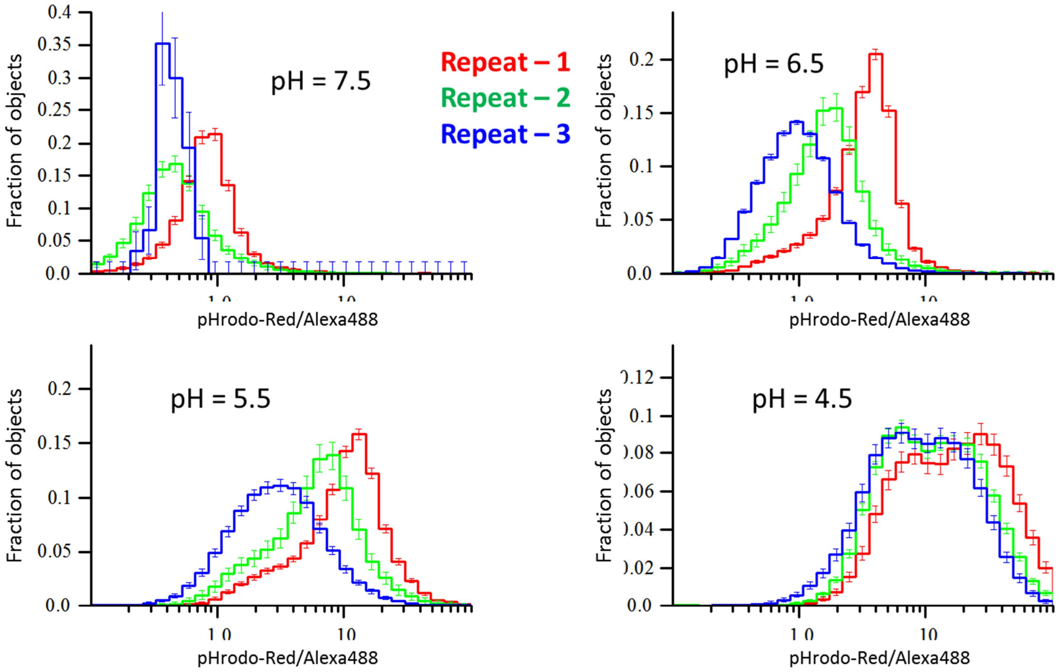

Figure S37. Distribution ratios of integral intensities of pHRodo-Red/Alexa-488 in calibration measurements in three independent repeats of experiment. The pH of calibration buffer is denoted on respective panels. Colors of curves denote experiment repeats. Error bar denotes SEM between biological replicates (n = 3).

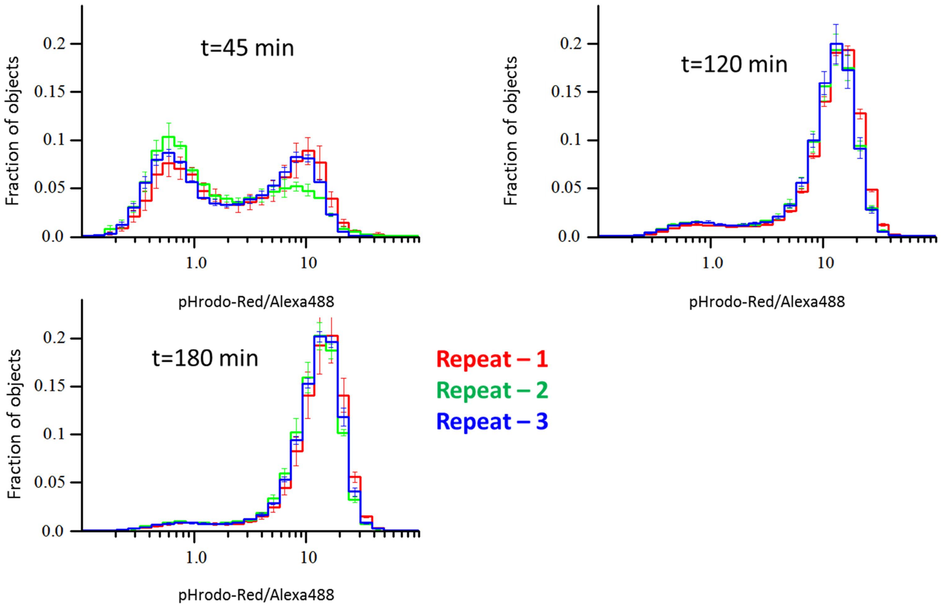

Figure S38. Distribution of objects ratios of integral intensities of pHRodo-Red/Alexa-488 in live HeLa cells internalized with LDL-pHRodo-Red and LDL-Alexa-488 for the time period denoted on respective panels. Colors of curves denote repeat number of experiments. Error bar denotes SEM between biological replicates (n = 3).

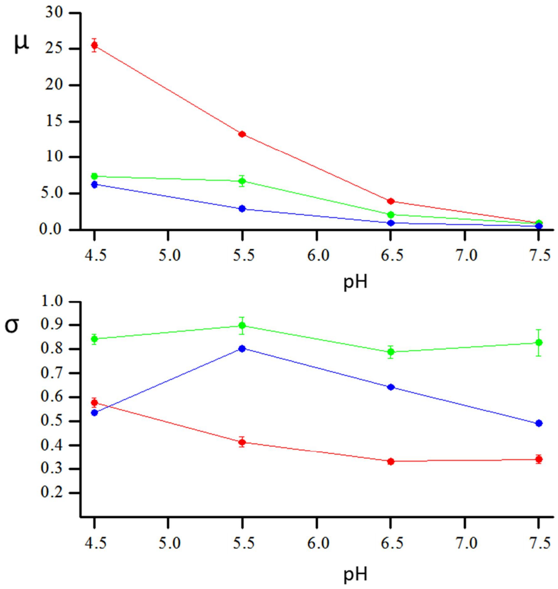

Figure S39: pH dependency of parameters μ and σ of log-normal components. Red, green and blue colors denote components with largest, middle and smallest ratios μ. The global fit was performed by global histogram fit procedure in GraphView application of MotionTracking software. The error bar denotes parameter estimation uncertainty estimation by inverse Hessian of log-likelihood.

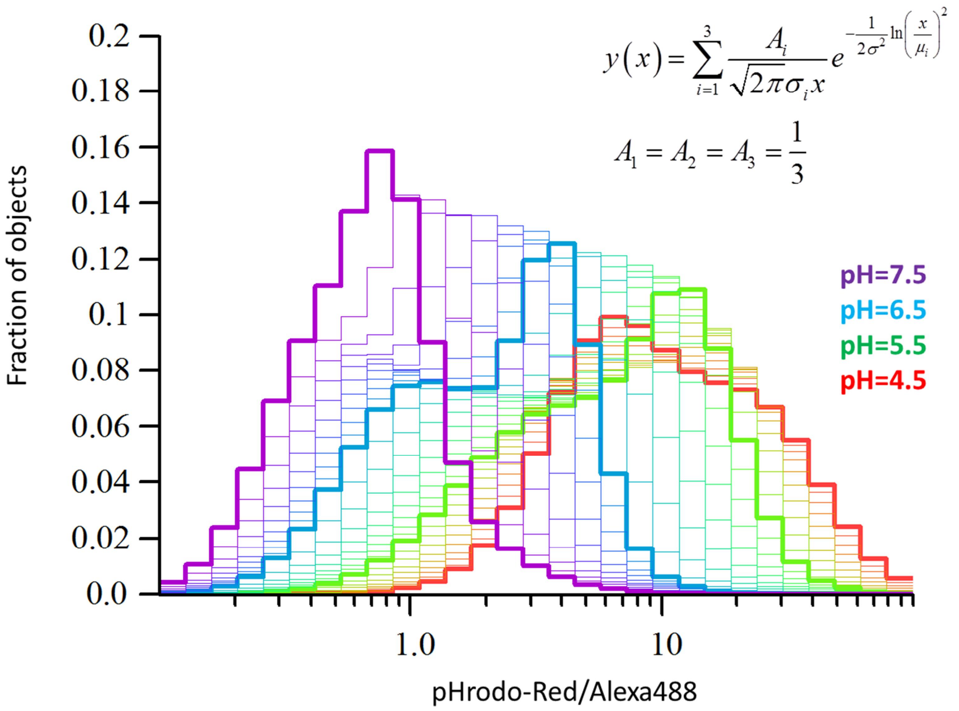

Figure S40: Predicted distribution of ratios with equal contributions components at pH in range from 4.5 to 7.5.

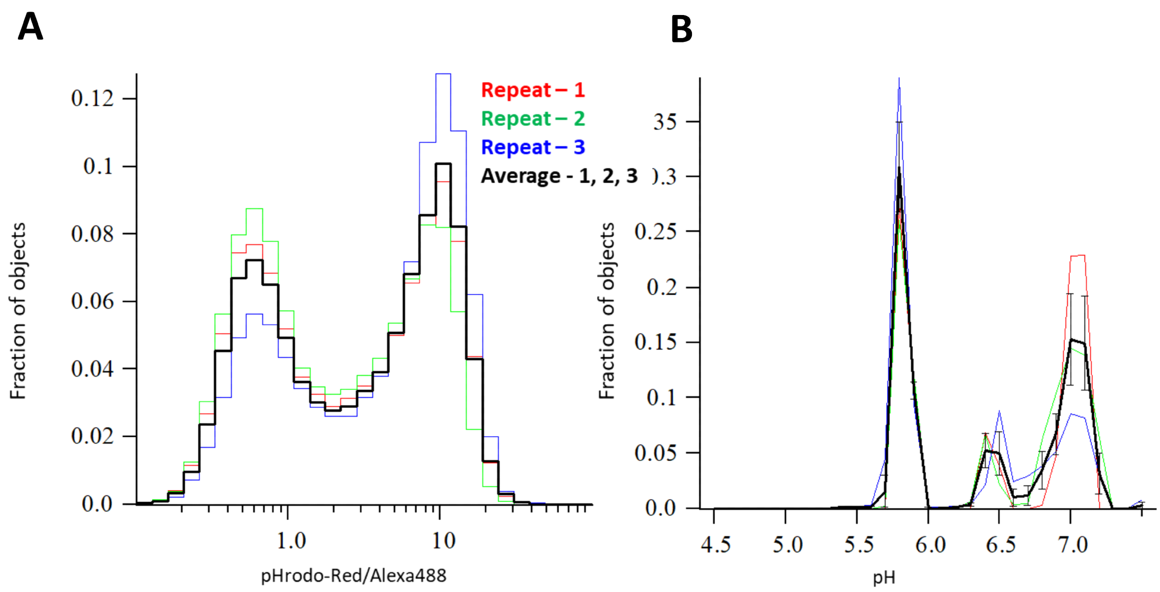

Figure S41. **A**. Experimental distribution of intensities ratios (t=45min). **B.** Fitted distribution of pH. Colors encode experimental repeats. Black line is average of repeats. Error bars on average curve are standard error of means (SEM).

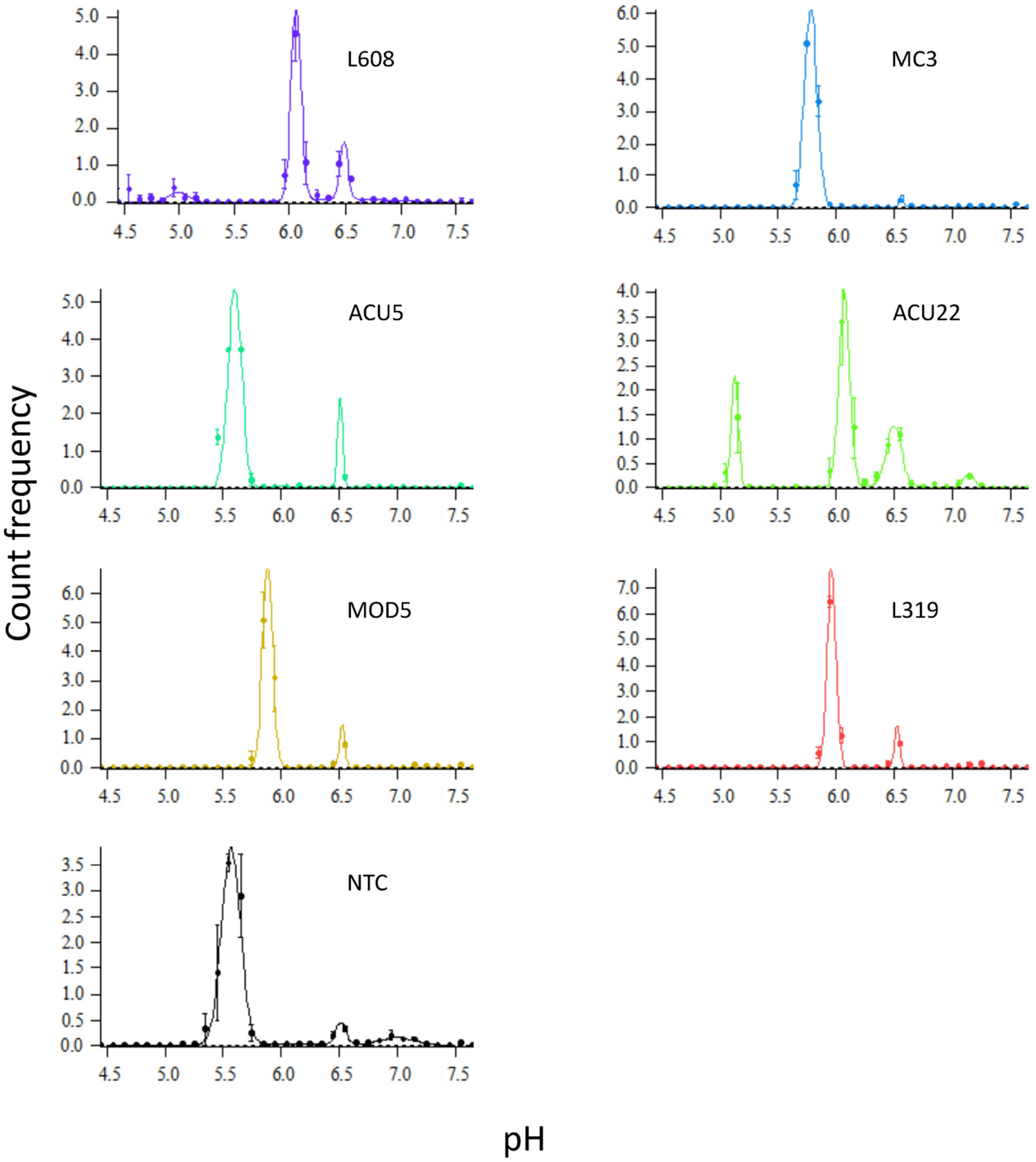

Figure S42. Example figure of pH estimation by Gaussian fitting for data presented in Figure 3 A. The widths of fitted Gaussian are used for pH uncertainty estimation in Supplementary Table 2
